# Remote C–H bond cooperation strategy enabled silver catalyzed borrowing hydrogen reactions†

**DOI:** 10.1039/d4sc05486e

**Published:** 2024-11-22

**Authors:** Zhe Chen, Laofeng Ouyang, Ning Wang, Weikang Li, Zhuofeng Ke

**Affiliations:** a School of Materials Science and Engineering, PCFM Lab, the Key Laboratory of Low-carbon Chemistry & Energy Conservation of Guangdong Province, Sun Yat-sen University Guangzhou 510006 P. R. China kezhf3@mail.sysu.edu.cn; b School of Chemistry, Sun Yat-sen University Guangzhou 510275 P. R. China

## Abstract

Metal–ligand cooperation (MLC) is an essential strategy in transition metal catalysis. Traditional NH-based and OH-based MLC catalysts, as well as the later developed (de)aromatization strategy, have been widely applied in atom-economic borrowing hydrogen/hydrogen auto-transfer (BH/HA) reactions. However, these conventional MLC approaches are challenging for low-coordination and low-activity coinage metal complexes, arising from the instability during (de)protonation on the coordination atom, the constraint in linear coordination, and possible poisoning due to extra functional sites. Herein, we demonstrate a remote C–H bond cooperation strategy that enables the unprecedented homogeneous Ag(i)-catalyzed BH/HA reaction. The covalent C–H bifunctional site well facilitates (de)hydrogenation, especially under the low coordination circumstances of d^10^ Ag(i). The strong electron-donating bis-*N*-heterocyclic carbene (NHC) ligand stabilizes the silver–hydride and stimulates the hydride activity on the *trans*-position of ligands. Mechanistic studies implicate the plausible remote assistance of the dissociative NHC arm in facilitating BH/HA reactions. Our findings emphasize the potential of the remote C–H bond cooperation strategy for low coordination metals in catalyzing BH/HA reactions and broadening MLC catalysts to d^10^ coinage metals.

## Abstract 2

Metal–ligand cooperation (MLC) is an essential strategy in transition metal catalysis. Herein, we demonstrate a remote C–H bond cooperation strategy that enables the unprecedented homogeneous Ag(i)-catalyzed BH/HA reaction.

## Graphical abstract

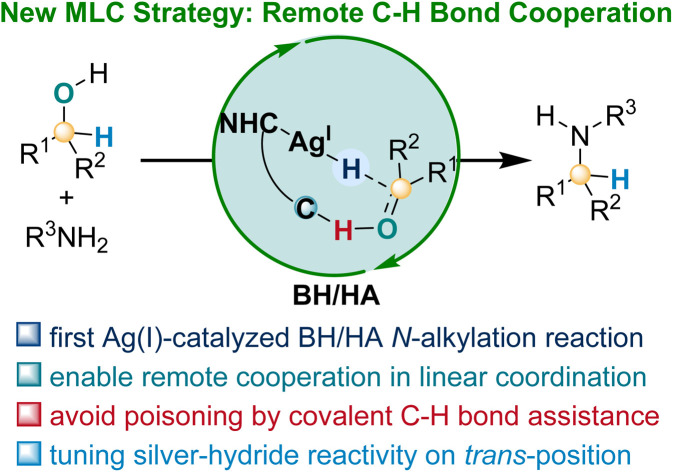

## Introduction

**Fig. 1:**
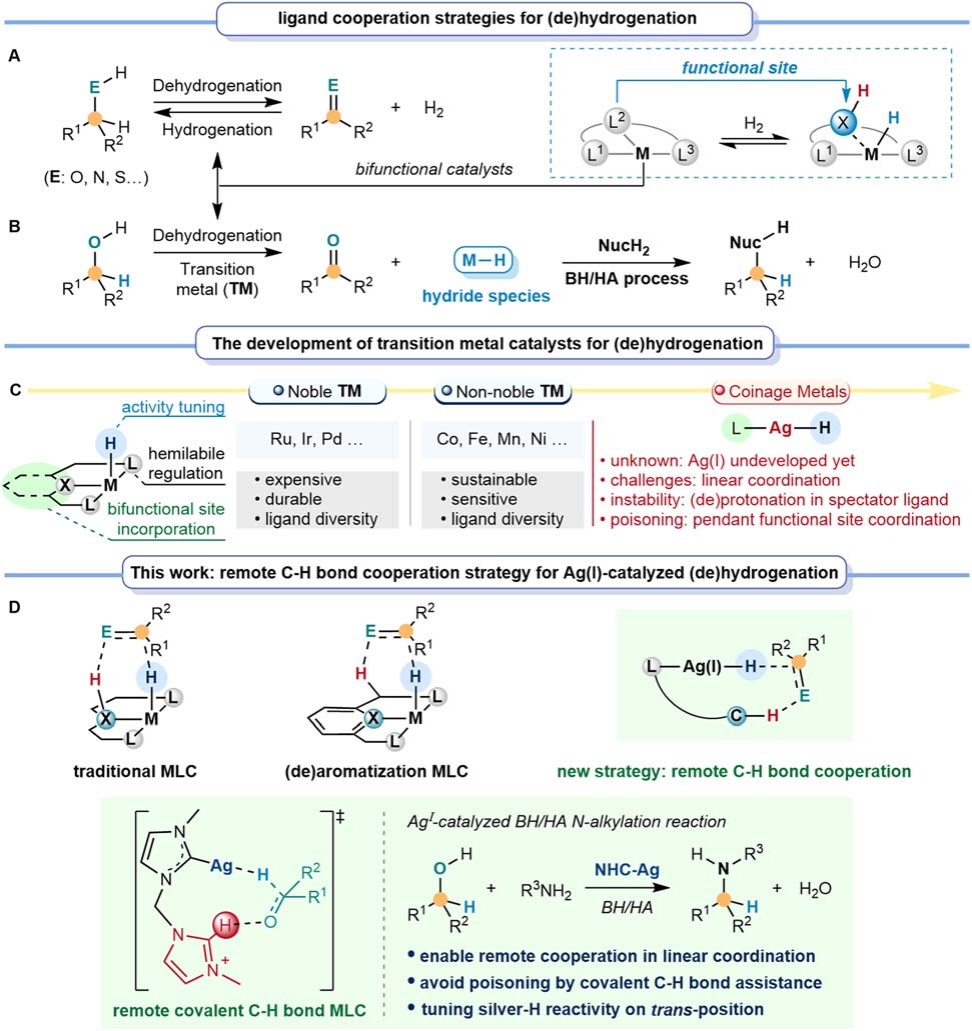
Remote C–H bond cooperation enables silver-catalyzed BH/HA reactions. (A and B) Ligand cooperation strategies for dehydrogenation and hydrogenation. (C) The development of transition metal catalysts for dehydrogenation and hydrogenation. (D) Remote covalent C–H bond cooperation strategy for silver-catalyzed dehydrogenation and hydrogenation.

Catalytic dehydrogenation and hydrogenation of sustainable feedstock offer efficient synthesis methods to selectively synthesize more valuable compounds, thus circumventing extensive pre-activation and functionalization steps.^1–3^ Different from classical metal catalysis, bifunctional catalysts exhibit unique catalytic potential in dehydrogenation and hydrogenation, benefiting significantly from metal–ligand cooperation (MLC, Fig. 1A).^4–7^ Over the past few decades, utilizing the significant potential of the MLC strategy for the dehydrogenation and hydrogenation of alcohols, many efforts have been made in tandem reactions with nucleophiles (NucH_2_) through the borrowing hydrogen/hydrogen auto-transfer (BH/HA) strategy.^8–10^ The BH/HA process is a highly atom-economic and environmentally benign process using inactivated alcohol as both a hydrogen source and a sustainable reactant, with water as the only by-product.

Achieving an efficient BH/HA reaction requires a metal-catalyzed system with an appropriately active metal-hydride species, which could be meticulously regulated by the electronic effect and the cooperation feature of the ligands (Fig. 1B).^11–13^ Noyori-type metal–ligand cooperation (M/NH) catalysts take full advantage of the nitrogen site for bifunctional catalysis.^14^ Since then, nitrogen-site-based MLC catalysts have been developed as a classical type of catalyst for BH/HA reactions, using precious transition metals, such as Rh, Ir, Pd, and others, as metal centers.^15–18^ Later, interest has increasingly shifted toward developing non-noble transition metal catalysts, mainly including Mn, Fe, Co, Ni, and so on, due to the demand for low-cost, sustainable, and environmental benefits.^19–23^ These precious and non-noble transition metal catalysts are usually characterized by high coordination numbers, facilitating catalyst design due to the ligand diversity. They can bear spectator ligand(s) to stabilize the active species and tune the activity. Meanwhile, it can also introduce the hemilabile ligand to improve the substrate interaction and promote the reaction. More importantly, it enables the incorporation of different types of bifunctional sites in the ligand to realize MLC catalysis (Fig. 1C).^24,25^ Besides the traditional coordinating nitrogen-based MLC strategy in BH/HA catalysis^26–30^ (Fig. 1D), a non-coordinating oxygen atom in the cyclopentadienone ligand was also developed for MLC catalysis in Shvo's system.^31,32^ Himeda, Yamaguchi, Fujita *et al.* utilized the –OH group in bipyridine and related systems to achieve MLC (de)hydrogenations.^33–35^ Differently, another type of MLC catalyst (Fig. 1D) was developed *via* (de)aromatization, in which the tautomerization of the pyridine-based pincer ligand provides the functional site for (de)hydrogenations.^36–39^ Other developments of pincer ligands by replacing the CH_2_ arm with NH groups were also introduced.^40,41^

When it comes to d^10^ coinage metals, there are still only a few examples of successful BH/HA catalysts,^42^ especially for silver metal, which offers a balanced choice between the expensive precious transition metal complexes and the air-moisture sensitive non-noble transition metal complexes. However, the design of a bifunctional ligand or hemilabile ligand for silver catalysts becomes extremely challenging, due to the inherent linear and low-coordination nature of Ag(i) complexes (except for some weak interactions and metal–metal interactions in cluster species).^43,44^ Firstly, conventional MLC is limited because the (de)protonation of the spectator ligand to sever as the functional site would result in destabilization for the low-coordinating coinage metal complexes. Secondly, the (de)aromatization MLC within the pincer framework would be impractical due to the constraint in linear coordination. Furthermore, the introduction of conventional pendant functional sites for MLC typically exhibits certain toxicity towards coinage metals, further impeding the exposure of active sites. These challenges stimulate us to seek a new strategy to enable efficient MLC catalysis for silver metal. With this new strategy, the designed ligand(s) should also tackle the delicate task of tuning the key species, the silver–hydride's stability and reactivity towards BH/HA.

In the present work, we introduce the concept of a remote C–H bond cooperation strategy for MLC catalysis (Fig. 1D). We envision that this remote C–H bond cooperation in catalytic (de)hydrogenation not only overcomes the constraint in linear coordination, but also avoids the possible poisoning of the catalytic active species by employing covalent C–H bonds as assisting sites for d^10^ metal complexes. Herein, we demonstrate that the new remote C–H bond cooperation strategy enables unprecedented homogeneous BH/HA reactions for low-coordinated d^10^ coinage metal catalysts. The strong electron-donating bis-*N*-heterocyclic carbene (NHC) ligand is crucial for stabilizing the silver–hydride and stimulating the hydride activity at the *trans*-position of ligands. Mechanistic studies imply that the covalent C–H bond from the dissociative NHC arm remotely assists in facilitating dehydrogenation and hydrogenation, especially under the low coordination circumstances of the d^10^ metal centers. Our findings emphasize the potential of the remote C–H bond cooperation strategy for low coordination metals in catalyzing (de)hydrogenation and broaden the generality of catalysts to d^10^ coinage metals.

## Results and discussion

### Activation of d^10^ silver metal

**Fig. 2:**
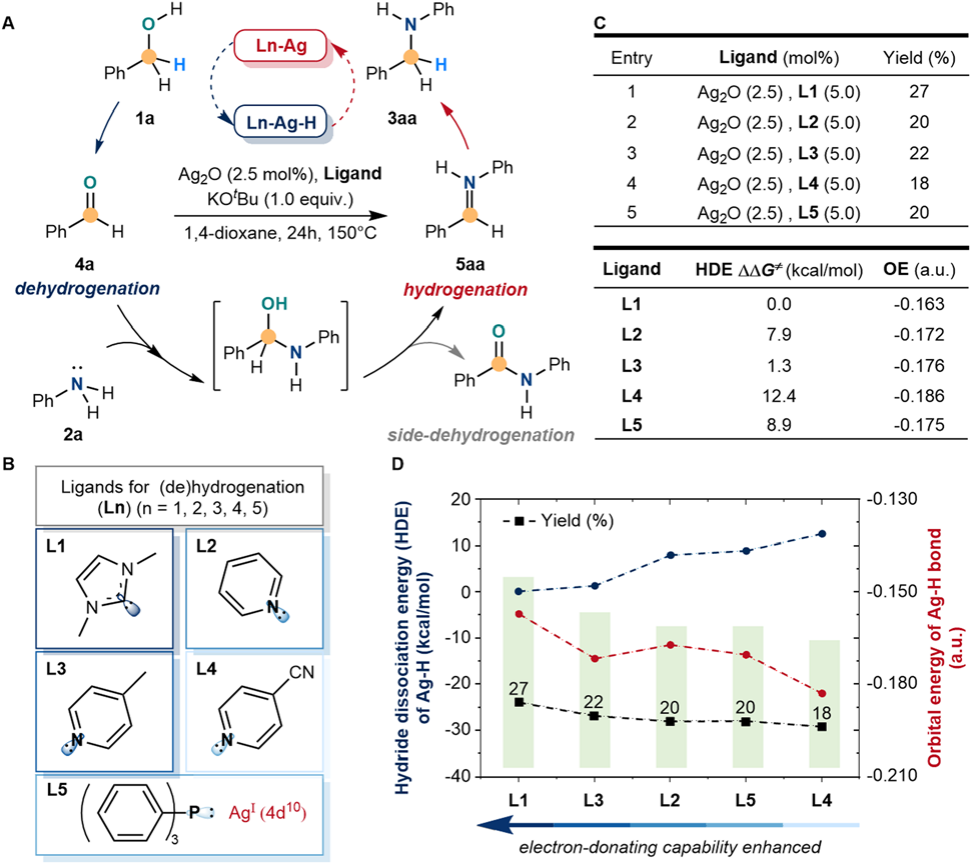
Activation of the d^10^ silver-catalyzed BH/HA reaction by ligand stimulation. (A) Model reaction for silver-catalyzed (de)hydrogenation. (B) Screening of electron-donating ligands. (C) Reaction yield of *N*-alkylation, hydride dissociation energy (HDE) of silver–hydride, and orbital energy (OE) of the silver–hydride bond. (D) Comparison of ligand catalyzed silver *N*-alkylation reactions. Model reaction conditions: benzyl alcohol (1a, 1.0 mmol), aniline (2a, 0.5 mmol), Ag_2_O (2.5 mol%), ligands (5.0 mol%), KO^*t*^Bu (1.0 equiv.), 1.4-dioxane (1 mL), 150 °C, and 24 h.

We initially sought to identify a simple model system that would enable us to explore ligand cooperation strategies for silver-catalyzed (de)hydrogenation. We initiated the exploration with a common model reaction by the *N*-alkylation of aniline (2a) with benzyl alcohol (1a) to *N*-benzyl aniline (3aa), which involved the dehydrogenation of benzyl alcohol. However, when the amino alcohol intermediate was formed, the (de)hydrogenation of silver could be achieved only selectively by further dehydration and hydrogenation of 5aa rather than further side-dehydrogenation (Fig. 2A). Inspired by the developed Mn(i), Mo(0), Fe(ii), Cr(0), and other catalytic systems, the electron-donating ability of ligands could stimulate the activity of metal-hydride by strong σ donation and π back donation.^45–48^ Hence, the commonly accessible ligands were screened (Fig. 2B), including C-, N-, and P-coordinated. Under strong basic and high-temperature conditions, 1,3-dimethyl imidazolium hexafluoro-phosphate (L1), pyridine (L2), 4-methylpyridine (L3), 4-cyanopyridine (L4) and triphenylphosphine (L5) were used as simple model ligands to catalyze *N*-alkylation, which represented the most common electron-donating ligands in the literature. Meanwhile, the low reactivity of the *N*-alkylation product (27% yield) was detected with L1, which was higher than others (Fig. 2C). Despite the above results showing low reactivity, the side-dehydrogenation products were not detected and tended to hydrogenate under these conditions. Meanwhile, the C-ligands were more active in stimulating silver than the N- and P-ligands.

According to the results, a preliminary theoretical study of silver–hydride with different ligands was carried out by comparing the hydride dissociation energy (HDE) and orbital energy of the silver–hydride bond to reveal the regulation (Fig. 2D). The C-ligand (L1) had a lower hydride dissociation energy and higher orbital energy, which suggested that the electron-donating ability of ligands influenced the activity of silver. When the electron-donating ability enhanced, the activity of silver–hydride was more active and more favorable for the alkylation reaction. The electron-donating ability of N-ligands (L2–L4) and the P-ligand (L5) was lower than that of the C-ligand (L1), finally, showing lower activity and alkylation yields than L1.

### Establishment of the silver catalytic system

**Fig. 3:**
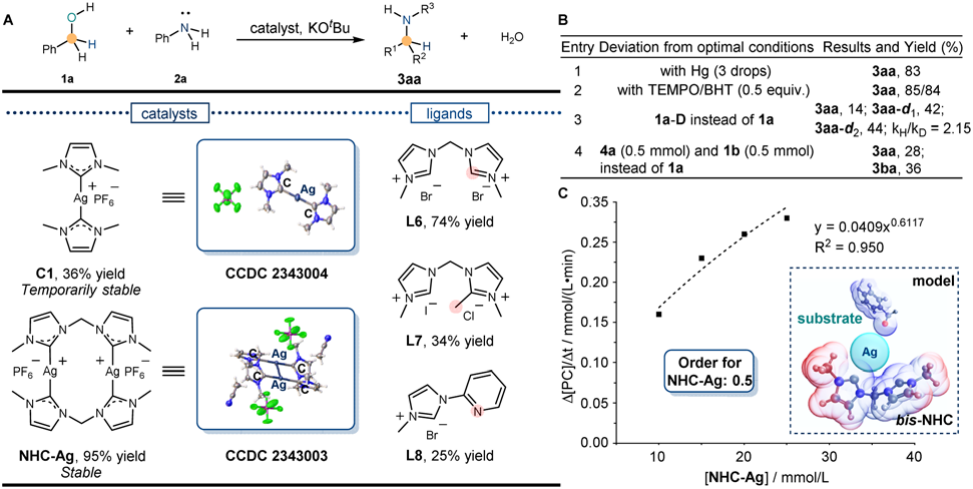
Establishment of the silver catalytic system by preliminary control experiments. (A) Reaction yield of *N*-alkylation. Model conditions: benzyl alcohol (1a, 1.0 mmol), aniline (2a, 0.5 mmol), ligands or catalysts (5.0 mol%), KO^*t*^Bu (1.0 equiv.), 1.4-dioxane (1 mL), 150 °C, and 24 h. (B) Preliminary control experiments. Optimal conditions: benzyl alcohol (1a, 1.0 mmol), aniline (2a, 0.5 mmol), NHC-Ag (2.5 mol%), KO^*t*^Bu (1.0 equiv.), 1.4-dioxane (1 mL), 150 °C, and 24 h. (C) Reaction order for NHC-Ag and the model for the silver catalytic system.

Following the above discovery and relevant literature, the C-coordinated silver complexes were synthesized. Due to the stabilization of the *N*-heterocyclic carbene (NHC) ligand at the silver center, the silver catalyst C1 (CCDC 2343004) was successfully synthesized by using L1. The still low reactivity of the *N*-alkylation product (36% yield) was detected in the reaction (Fig. 3A). Considering the influence of the temporary stability of C1, meanwhile, a more stable NHC-Ag (CCDC 2343003) catalyst was synthesized and the potential functional part was introduced to catalyze the reaction. Unexpectedly, the desired alkylation product was obtained in 74% yield with the bis-NHC ligand (L6) and 95% yield with NHC-Ag (Table S1† entry 1). When the activity was stimulated, subsequently, an investigation was conducted to explore the reactivity influenced by the amount of NHC ligand. Despite using the same conditions, the yield remained at a low level (30%, Table S1† entry 2), indicating the bis-NHC ligand L6 underwent a unique function to eventually achieve silver-catalyzed *N*-alkylation. To further validate the results, ligands L7 and L8 were synthesized to catalyze the reaction, which inhibited the reactions. Yields of 34% and 25% were observed, respectively, indicating that it was the bis-NHC ligand L6 that promoted the reaction. Thereafter, *N*-alkylation yields were further optimized using the NHC-Ag catalyst to catalyze the reaction (Table S1† entry 14). Finally, the optimal reaction conditions were determined: 1a (1.0 mmol), 2a (0.5 mmol), NHC-Ag (2.5 mol%), KO^*t*^Bu (0.7 equiv.), 1,4-dioxane (1 mL), 150 °C, and 24 h. Under the optimal conditions, the desired alkylation product was obtained in a 95% isolated yield.

### Origin of silver-catalyzed alkylation activity

**Fig. 4:**
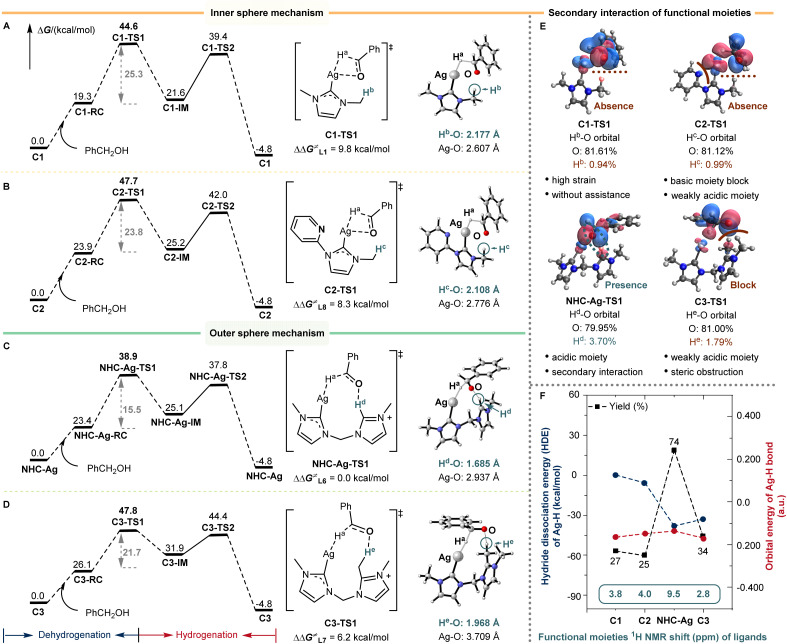
Origin of silver-catalyzed alkylation activity. (A) L1 and C1 without the assistance of a covalent C–H bond catalyzed silver *N*-alkylation *via* the inner sphere mechanism. (B) L8 and C2 with the assistance of a covalent C–H bond catalyzed silver *N*-alkylation *via* the inner sphere mechanism. The remote functional site is a weakly acidic C–H bond. (C) L6 and NHC-Ag with the assistance of a covalent C–H bond catalyzed silver *N*-alkylation *via* the outer sphere mechanism. The remote functional site is an acidic C–H bond. Secondary sphere interaction assists the (de)hydrogenation. (D) L7 and C3 with the assistance of a covalent C–H bond catalyzed silver *N*-alkylation *via* the outer sphere mechanism. The remote functional site is a weakly acidic C–H bond. Steric obstruction blocks the secondary sphere interaction. (E) Component analysis of O–H orbitals. (F) Hydride dissociation energy (HDE) of Ag–H and orbital energy of the Ag–H bond of ligands (L1, L6, L7, and L8). Functional moiety ^1^H NMR shifts (*δ* ppm) of ligands (L1, L6, L7, and L8).

Based on the understanding of the reaction mechanism and the establishment of the reaction model, the bis-NHC silver catalyst successfully facilitates (de)hydrogenation and enables the BH/HA *N*-alkylation reaction. We are keenly interested in the role of the active species that involves one silver center and one bis-NHC ligand. The DFT calculations of the RDS were performed concerning the role of the bis-NHC ligand. The differences in the barrier of dehydrogenation (ΔΔ*G*^≠^) for L1, L7, and L8 silver species were calculated to be 9.8, 6.2, and 8.3 kcal mol^−1^, respectively (Fig. 4A–D). For the low coordinated Ag(i) center, with mono-NHC L1 as the auxiliary ligand, the dehydrogenation of alcohol proceeded through an inner-sphere transition state C1-TS1, which resulted in a high distortion energy due to the four-membered ring transition state structure (Fig. 4A).

Similarly, L8 also proceeded through an inner-sphere transition state C2-TS1, and the four-membered ring transition state structure was disfavored for the dehydrogenation (Fig. 4B). Meanwhile, the side pyridine of L8 may also poison the silver, leading to the lower yields observed previously (25% for L8). Notably, the bis-NHC ligand L6 could well release the ring strain with the assistance of the dissociative NHC arm, forming an outer-sphere transition state NHC-Ag-TS1 (Fig. 4C). As can be seen in NHC-Ag-TS1, the covalent C–H bond of the dissociative NHC arm could form a significant H-bond with the benzyl alcohol anion. The distance of H^d^–O in NHC-Ag-TS1 was 1.685 Å, indicating strong H-bonding assistance with respect to ligands L1 and L8 (2.177 Å and 2.108 Å, respectively). After blocking this C–H moiety in ligand L7, the H-bond assistance was absent (C3-TS1 and Fig. 4D), resulting in a 6.2 kcal mol^−1^ reaction barrier difference. Meanwhile, the distance of H^e^–O in C3-TS1 was 1.968 Å, and the distance of H^e^–O was shorter than that of H^d^–O (1.685 Å), indicating that the lack of the secondary sphere interaction from covalent C–H bond cooperation inhibited the dehydrogenation.

The component analysis of O–H orbitals well illustrated the importance of the H-bonding interaction of L6 during dehydrogenation (H^d^ 3.70% in NHC-Ag-TS1, H^b^ 0.94% in C1-TS1, H^c^ 0.99% in C2-TS1, and H^e^ 1.79% in C3-TS1, Fig. 4E). According to the comparison of the above ligands, the bis-NHC ligand not only stabilized and activated the silver–hydride species, but also promoted the reaction in a bifunctional manner due to the covalent C–H bond of the dissociative NHC arm. The high strain, weakly acidic remote C–H moiety, and steric obstruction inhibited the (de)hydrogenation. Besides, the hydride dissociation energy (HDE) of silver–hydride and the orbital energy of the Ag–H bond for ligands were shown (Fig. 4F). The bis-NHC well stimulated the activity of silver–hydride, leading to lower HDE (ΔΔ*G*^≠^ = −38.2 kcal mol^−1^) and higher orbital energy of Ag–H (−0.136 a.u., Fig. 4F and Table S2†), resulting in higher activity than the others. Besides, the functional moiety ^1^H NMR shifts of ligands were correlated with the strength of the secondary sphere interaction, caused by the relative remote distance from catalytic centers. The ^1^H NMR shift of C–H^d^ in L6 was in a lower field (*δ* 9.5 ppm) than that of the others (C–H^b^*δ* 3.8 ppm in L1, C–H^c^*δ* 4.0 ppm in L8, and C–H^e^*δ* 2.8 ppm in L7, respectively), which suggested that the acidic nature of C–H had influenced the secondary sphere interaction. We further detected the dissociative functional arm by ^1^H NMR and found the appearance of a new signal (*δ* 9.78 ppm) when introducing 1a into the NHC-Ag (0.06 mmol) and KO^*t*^Bu (0.125 mmol) solution (1 mL 1,4-dioxane) (Fig. S9-4†). These results unveiled the unique cooperative assistance of bis-NHC for the reaction. The strategy of remote C–H bond cooperation successfully enabled silver-catalyzed (de)hydrogenation and was applied to the BH/HA reaction.

### Applications of silver-catalyzed *N*-alkylation

**Fig. 5:**
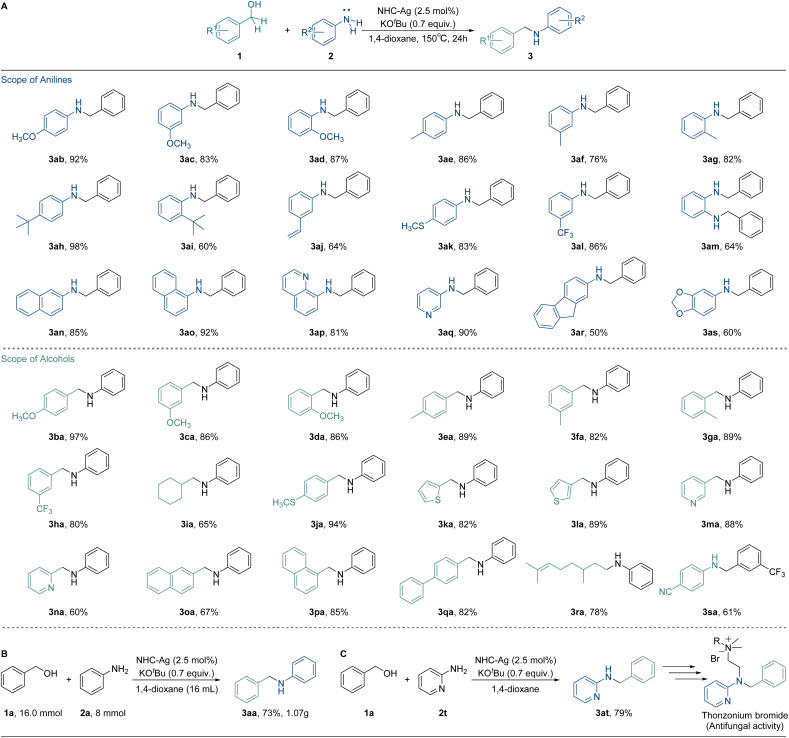
Substrate scope of silver catalyzed *N*-alkylation and applications. (A) Scope of substrates. Optimal conditions: anilines (2, 0.5 mmol), alcohols (1, 1.0 mmol), NHC-Ag (2.5 mol%), KO^*t*^Bu (0.7 equiv.), 1.4-dioxane (1 mL), 150 °C, and 24 h. (B) Gram-scale reaction. Conditions: aniline (2a, 8.0 mmol), benzyl alcohol (1a, 16.0 mmol), NHC-Ag (2.5 mol%), KO^*t*^Bu (0.7 equiv.), 1.4-dioxane (16 mL), 150 °C, and 24 h. (C) Synthetic application.

As the mechanism was revealed clearly, a variety of substrates were tested to probe the versatility of our catalytic system using the optimal conditions (Fig. 5A). To our satisfaction, various anilines were found to be suitable substrates and afforded the desired *N*-alkylation products smoothly. Specifically, benzyl anilines bearing electron-donating substituents yielded the desired *N*-benzyl aniline products 3ab to 3ak in good yields (60 to 98%). On the other hand, the yield for the substrate with the electron-withdrawing group (3al) provided a yield of 86%. Substrates with possessed extended aromatic systems also produced the desired products in attractive yields (85% for 3an, 92% for 3ao, and 81% for 3ap). Furthermore, heteroatom-containing substrates displayed good yields (3aq, 90%), although the yield decreased for heteroatom-containing substrates with electron-withdrawing groups (3as, 60%).

Subsequently, the compatibility of alcohols was also investigated. The benzyl alcohols bearing electron-donating groups (3ba to 3ga) exhibited excellent tolerance (82 to 97%). The yield for substrates with an electron-withdrawing group (3ha) reached up to 80%. Heteroatom-containing substrates efficiently converted to the corresponding products with attractive yields (82% for 3ka, 89% for 3la, and 88% for 3ma). Both 1-naphthyl alcohol and 4-benzyl benzyl alcohol displayed good yields (3pa, 85% and 3qa, 82%). Additionally, cyclohexyl methanol was also well converted to the corresponding product with a 65% yield for 3ia. Citronellol also exhibited a good yield (3ra, 78%). Notably, the substrates with electron-withdrawing groups in *para*- and *ortho*-positions did not convert to the corresponding products. In the case of halo-substituents, relatively harsh reaction conditions were required and dehalogenation occurred at high temperatures. The weakening in the nucleophilicity of the amines makes the generated benzaldehyde more susceptible to hydrogenation, preventing the further hydrogenation of imines. The nucleophilicity of silver–hydride and amine, as well as the competing hydrogenation reactions of benzaldehyde and imine, jointly determine the process of the *N*-alkylation reaction. A gram-scale reaction and synthetic application were carried out to further demonstrate the synthetic potentials of this methodology. 1.07 g of 3aa with 73% yield was delivered by coupling 1a with 2a (Fig. 5B). Furthermore, the aminopyridine derivative 3at could be efficiently synthesized in 79% yield by this silver system. 3at can be further used to synthesize the thonzonium bromide, which exhibits antifungal activity (Fig. 5C).

**Fig. 6:**
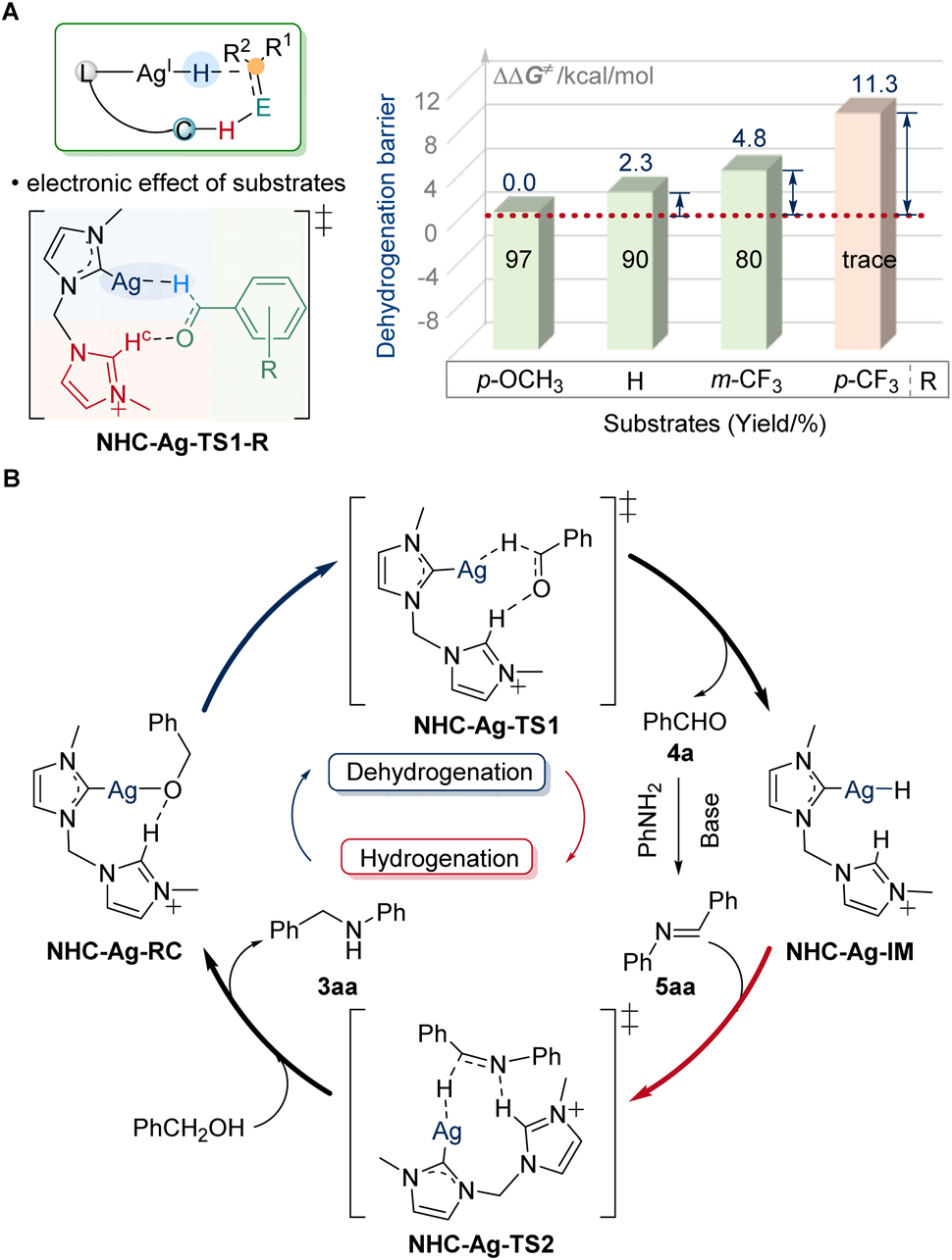
Electronic effect and plausible reaction pathway. (A) Electronic effect of substrates. (B) Catalytic cycle of the silver-catalyzed borrowing hydrogen reaction.

With the discovery of substrate scope, the crucial electronic influence on cooperative assistance during dehydrogenation was further highlighted. When changing the substituents to electron-withdrawing groups, the reaction will be suppressed. The difference in the barrier of dehydrogenation (ΔΔ*G*^≠^) for NHC-Ag-TS1-*p*-CF_3_ was 11.3 kcal mol^−1^, which was much higher than that of the others (2.3 kcal mol^−1^ for NHC-Ag-TS1, 4.8 kcal mol^−1^ for NHC-Ag-TS1-*m*-CF_3_, Fig. 6A). The low reactivity for NHC-Ag-TS1-*p*-CF_3_ was in good agreement with our study of the substrate scope. When the electron-donating ligands were used to regulate the low-activity metals, the prominence of the electronic effect from substrates influenced the dehydrogenation, significantly. Based on the experimental and computational studies, a plausible mechanism was depicted (Fig. 6B). The alkoxy-silver NHC-Ag-RC was generated by the deprotonation of alcohol substrates to start the catalytic cycle. Then the dehydrogenation occurred *via* the key transition state NHC-Ag-TS1, in which the hydride transferred to the silver center with the assistance of the dissociative NHC arm. The barrier of dehydrogenation was 38.9 kcal mol^−1^. The highly active silver–hydride NHC-Ag-IM then reduced the imine to form an amido complex, which was further protonated to release the *N*-alkylated product 3aa, regenerating the alkoxy complex NHC-Ag-RC for the next catalytic cycle. The barrier of hydrogenation was 37.8 kcal mol^−1^. The higher energy of the dehydrogenation than that of the hydrogenation suggested that the alcohol dehydrogenation was the RDS, which was in line with the results of the KIE observation.

## Conclusions

We demonstrate that a remote C–H bond cooperation strategy enables an unprecedented homogeneous silver-catalyzed BH/HA reaction, offering a new degree of freedom to metal–ligand cooperation. Improving the electron density of ligands could stimulate the activity of silver–hydride species. The bis-N-heterocyclic carbene ligand, while ensuring the stability and reactivity of the silver catalyst, facilitates dehydrogenation and hydrogenation by the possible remote covalent C–H bond assistance of the dissociative NHC arm. This strategy avoids the toxic and destabilizing effects of traditional MLC strategies for d^10^ low coordination metals. The outer sphere mechanism caused by covalent C–H bonds with certain acidity promotes the (de)hydrogenation of silver well. Our findings demonstrate the potential of the remote C–H bond cooperation strategy for low coordination metals in catalyzing BH/HA reactions and expand the horizons of bifunctional d^10^ coinage metal catalysis.

## Data availability

The data supporting this article have been included as part of the ESI.†

## Author contributions

Z. C. contributed to the acquisition, analysis, and interpretation of data and the preparation of the manuscript; L. O. contributed to the acquisition of data; N. W. contributed to the acquisition of data; W. L. contributed to the acquisition of data; Z. K. contributed to the conception of the work, supervision, and preparation of the manuscript.

## Conflicts of interest

The authors declare no competing financial interest.

## Supplementary Material

SC-OLF-D4SC05486E-s001

SC-OLF-D4SC05486E-s002
