# Supplementary material for: Remote C–H bond cooperation strategy enabled silver catalyzed borrowing hydrogen reactions
Source: Chem Sci. 2024 Nov 22;16(1):163–70. doi: 10.1039/d4sc05486e (PMC11603383; doi:10.1039/d4sc05486e)
Supplement: SC-016-D4SC05486E-s001 [file SC-016-D4SC05486E-s001.pdf]

Supplementary Information (SI) for Chemical Science.
This journal is © The Royal Society of Chemistry 2024

Electronic Supplementary Information

Remote C-H Bond Cooperation Strategy Enabled Silver Catalyzed Borrowing Hydrogen Reactions

Zhe Chen, Laofeng Ouyang, Ning Wang, Weikang Li, and Zhuofeng Ke*

*School of Materials Science and Engineering, PCFM Lab, the Key Laboratory of Low-carbon Chemistry & Energy Conservation of Guangdong Province, Sun Yat-sen University, Guangzhou, 510006, P. R. China.
*E-mail: kezhf3@mail.sysu.edu.cn

Table of Contents

I. General Information S3
II. Preparation of the Ligands and the Silver Complex S3
III. *N*-Alkylation of Amines with Alcohols S8
IV. Mechanism Details.. S66
V. Computational Details S68
VI. References S82

I. General Information

Unless otherwise stated, all manipulations were carried out under dry argon and dry nitrogen using conventional Schlenk or glove box techniques. All experiments involving catalysts were carried out under dark circumstances. Non-halogenated solvents were dried over sodium benzophenone ketyl and halogenated solvents over CaH_2. All other reagents were purchased from commercial sources and used without further purification. **TLC** was performed on silica gel GF254 (layer thickness 0.20-0.25 mm) and components were located by observation under UV light. Column chromatography was performed on silica gel (300-400 mesh) using 5% ethyl acetate/hexane (petroleum ether) as eluent. NMR spectra were recorded using a Bruker 400 MHz spectrometer, and chemical shifts were reported relative to TMS. GC analyses were recorded in a Shimadzu GC-2014C device equipped with a Wondacap 1 column.

II. Preparation of Ligands and Silver Complexes

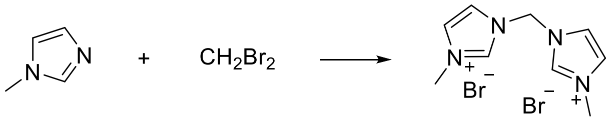

L6

Synthesis of 3, 3'-methylenebis(1-methyl-1H-imidazol-3-ium) bromide (L6)[1]

Following the general method using 1-methylimidazole (1.59 mL, 20 mmol) and dibromomethane (0.7 mL, 10 mmol) in a sealed tube, gave **L6** as a white solid. Yield: 3.04 g (90%). ^{1}H NMR (400 MHz, DMSO-d_6) δ 9.46 (s, -CH, 1H), 8.02 (s, =CH, 1H), 7.81 (s, =CH, 1H), 6.71 (s, $-CH_2$, 1H), 3.91 (s, $-CH_3$, 3H) ppm.

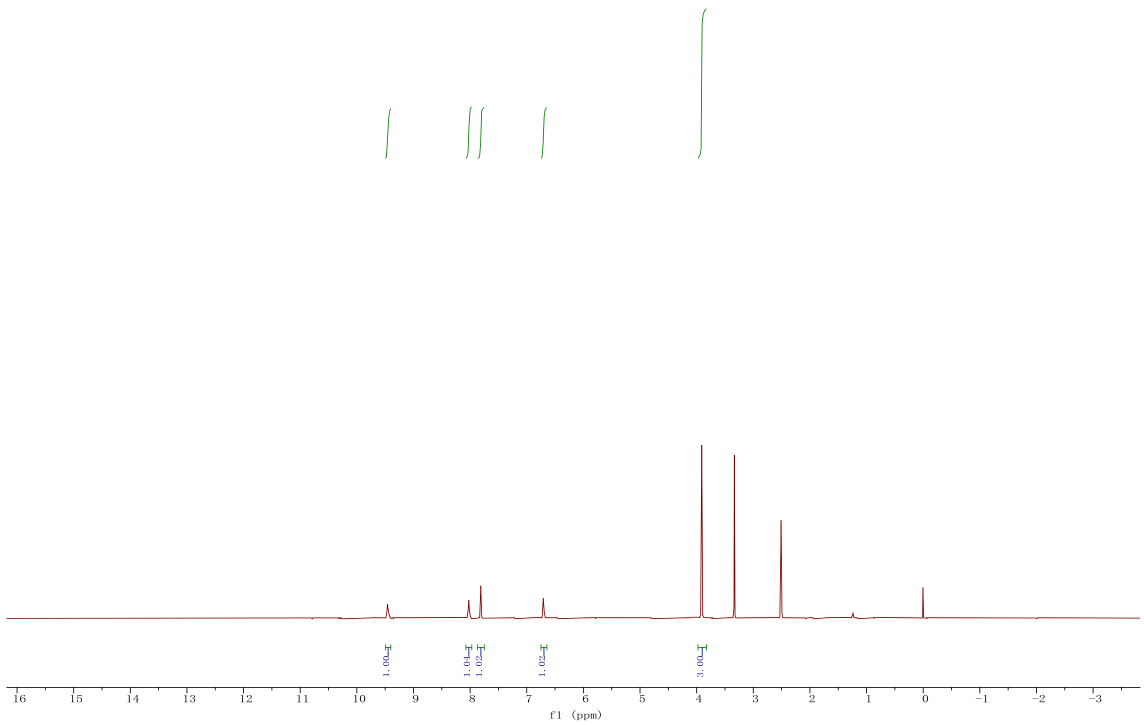

Figure S1. The ^{1}H NMR spectra of **L6** in DMSO-d_6.

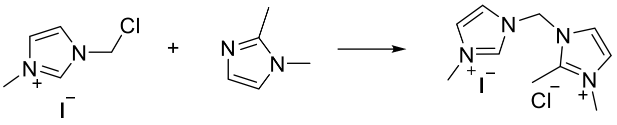

L7

Synthesis of 2,3-dimethyl-1-[(3-methyl-1H-imidazol-3-ium-1-yl)methyl]-1H-imidazol-3-ium iodide chloride (L7)

The 1-(chloromethyl)-3-methyl-1H-imidazol-3-ium iodide was synthesized following the general methods[2]. Then, 1-(chloromethyl)-3-methyl-1H-imidazol-3-ium iodide (207.2 mg, 0.8 mmol) and 1,2-dimethyl-1H imidazole (76.7 mg, 0.8 mmol) in THF (15 mL) were added in a sealed tube. The reaction was allowed to stir at 110 °C overnight. The white product (**L7**) was filtered and washed with ethyl acetate. The product was dried in vacuum. Yield: 200 mg (71%). ^{1}H NMR (400 MHz, DMSO-d_6) δ 9.67 (s, -CH, 1H), 8.16 – 8.07 (m, =CH, 2H), 7.81 (m, =CH, 1H), 7.75 (m, =CH, 1H), 6.76 (s, -CH_2, 2H), 3.90 (s, -CH_3, 3H), 3.78 (s, -CH_3, 3H), 2.79 (s, -CH_3, 3H) ppm. ^{13}C NMR (101 MHz, DMSO-d_6) δ = 10.57, 35.55, 36.66, 57.42, 121.36, 122.55, 123.69, 124.68, 138.47, 147.18 ppm. HRMS m/z: Calcd. for $C_{10}H_{16}N_4^{2+}$: 96.0682, Found: 96.0678.

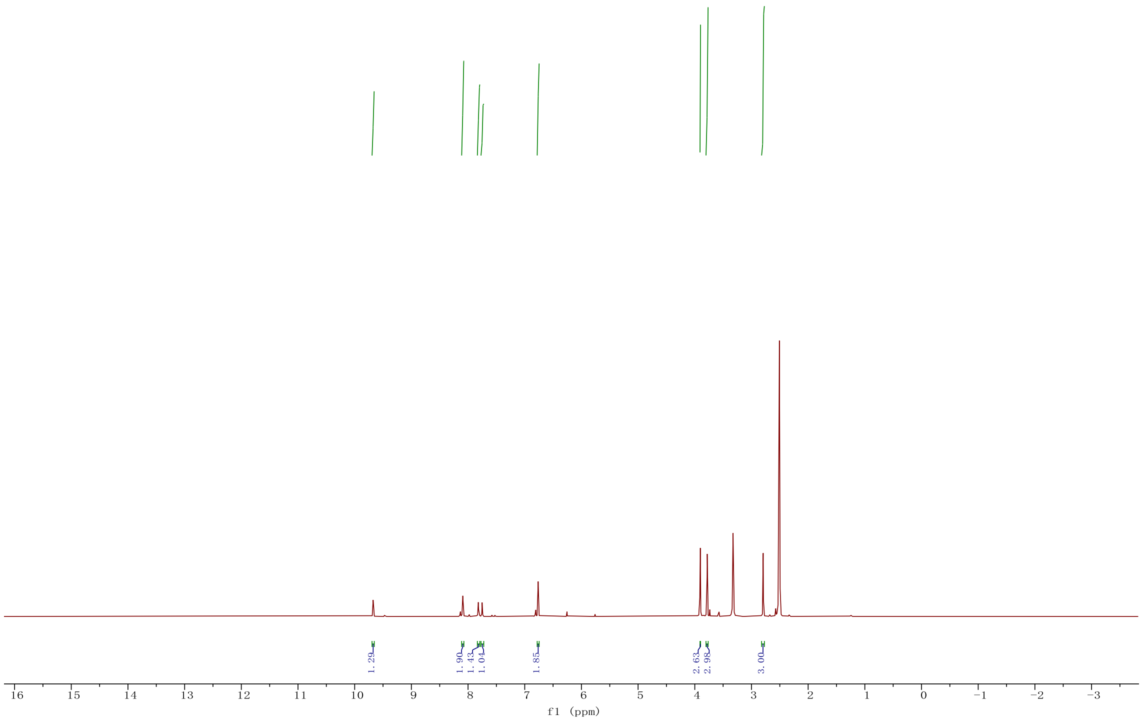

Figure S2a. The ^{1}H NMR spectra of **L7** in DMSO-d_6.

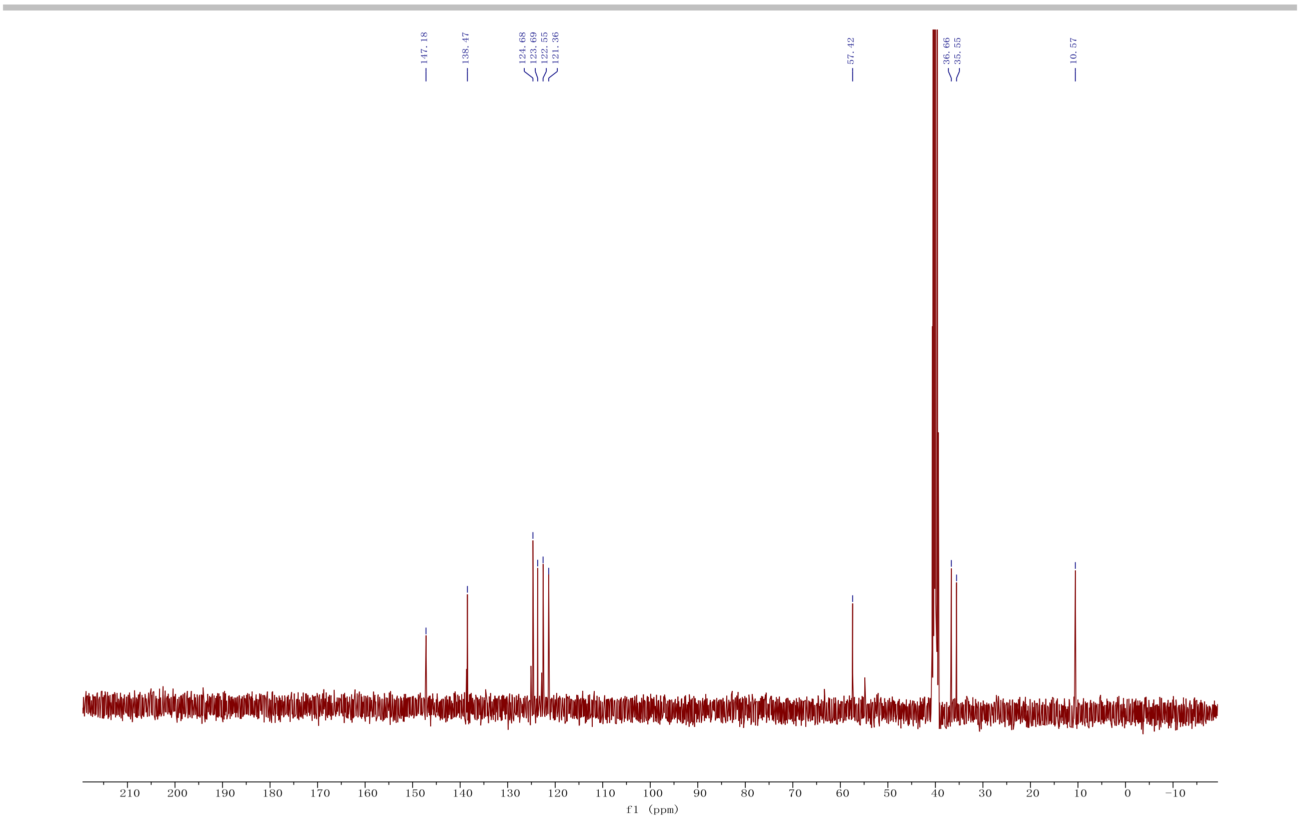

Figure S2b. The ^{13}C NMR spectra of **L7** in DMSO-d_6.

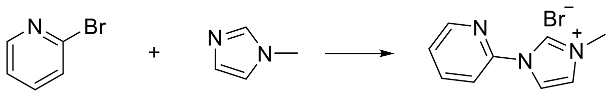

L8

Synthesis of 3-methyl-1-(pyridin-2-yl)-1H-imidazol-3-ium bromide (L8)[1]

2-bromopyridine (0.82 g, 10 mmol) and 1-methyl-1H-imidazole (1.58 g 10 mmol) were added in a sealed tube. The reaction was allowed to stir at 160 °C for 2 days. The solvent was evaporated to dryness and the corresponding crude was purified by column chromatography with silica gel (dichloromethane/methanol). The volatile solvent was removed to obtain the brown product. The product was dried under vacuum. Yield: 1.25 g (52 %). ^1H NMR (400 MHz, DMSO-d_6) δ 10.10 (s, -CH, 1H), 8.66 (s, =CH, 1H), 8.53 (s, =CH, 1H), 8.23 (s, =CH, 1H), 8.05 (d, J = 8.4 Hz, =CH, 1H), 7.99 (s, =CH, 1H), 7.65 (s, =CH, 1H), 4.00 (s, -CH_3, 3H).

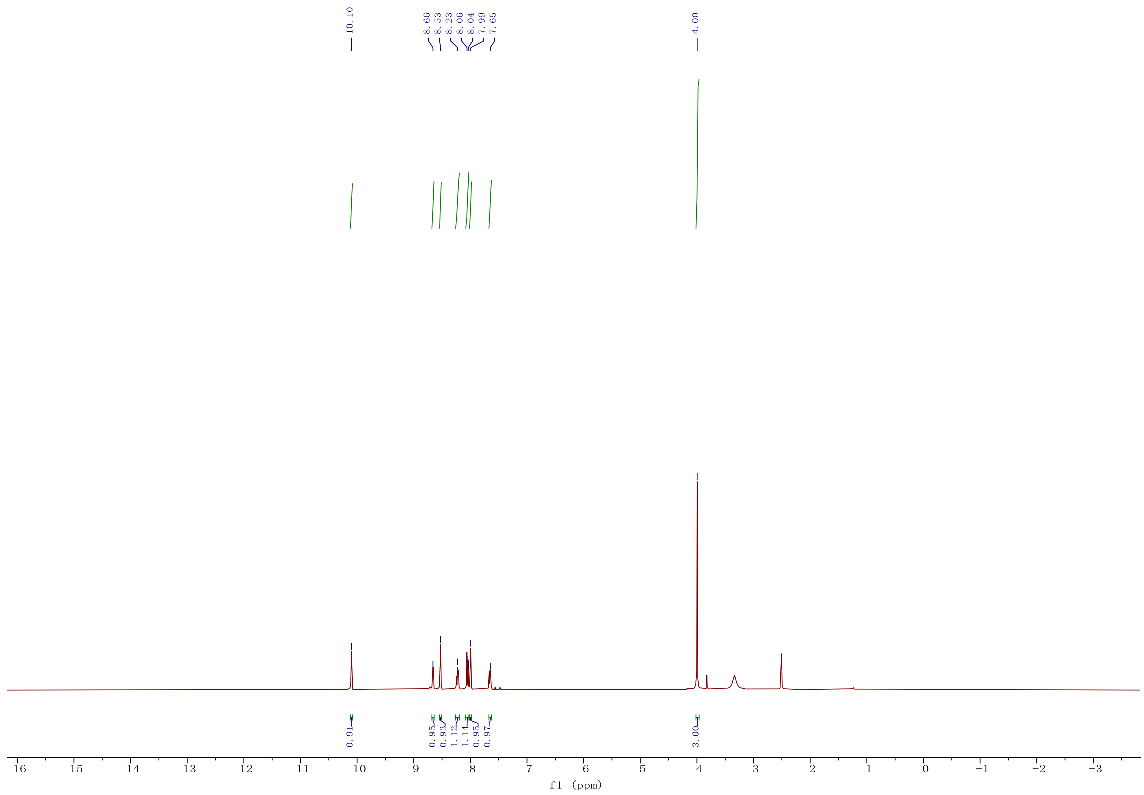

Figure S3. The ^{1}H NMR spectra of **L8** in DMSO-d_6.

Synthesis of C1[3]

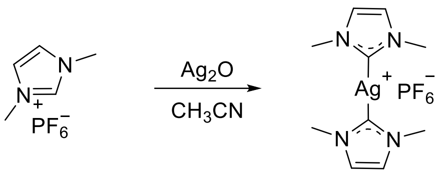

L1 **C1**

In a flask (50 mL), imidazolium salts **L1** (0.099 g, 0.75 mmol), Ag_2O (0.23 g, 1.0 mmol) and 10 mL CH_3CN were added in order. The mixture was stirred at R.T. for 2 days under dark circumstances. The slurry was filtered through a Celite pad. The volatile solvent was removed to obtain the brown product. The product was dried under vacuum. Yield: 108 mg (65%). ^{1}H NMR (400 MHz, DMSO-d_6) δ 7.42 (s, =CH, 1H), 3.83 (s, -CH_3, 3H). The single crystal suitable for X-ray diffraction analysis was obtained from slow diffusion of diethyl ether into an acetonitrile solution of **C1** at room temperature.

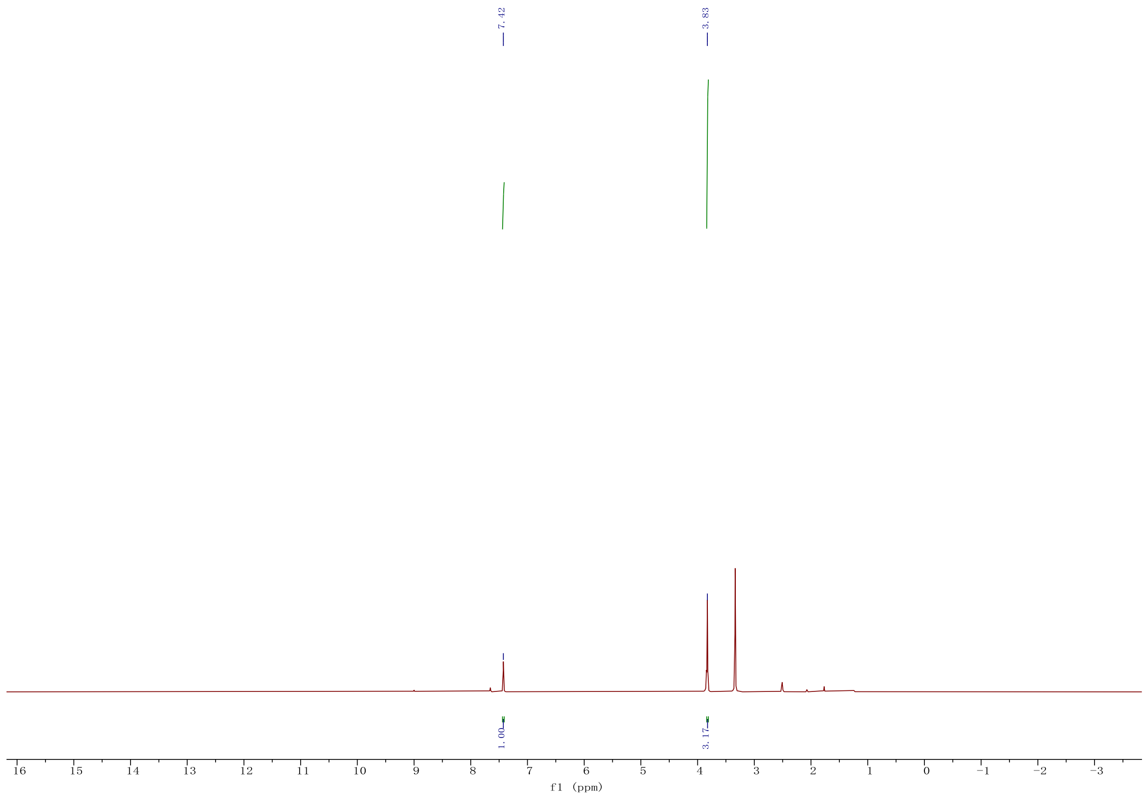

Figure S4-a. The ^{1}H NMR spectra of **C1** in DMSO-d_6.

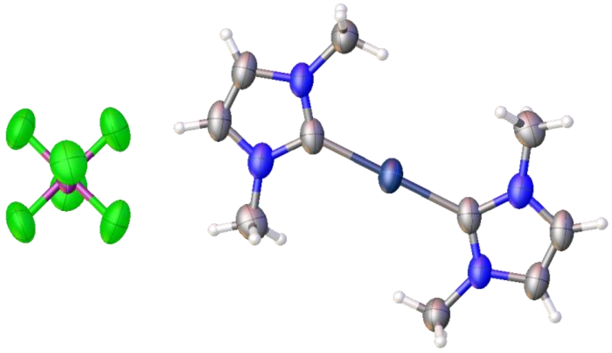

Figure S4-b. Crystal structure of **C1**. (**CCDC 2343004**)

Synthesis of NHC-Ag[4,5]

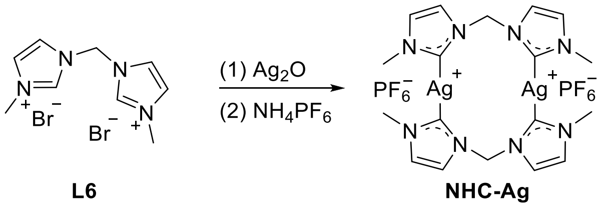

In a flask (50 mL), imidazolium salts **L6** (0.606 g, 2 mmol), Ag_2O (1.16 g, 5 mmol) and 10 mL H_2O were added in order. The mixture was stirred at R.T. for 15 minutes under dark circumstances. The slurry was filtered through a Celite pad. NH_4PF_6 (326mg, 2mmol) was added into the filtrate to precipitate **NHC-Ag** as white solid that was collected by filtration. The product was dried under vacuum. Yield: 836 mg (97%). ^{1}H NMR (400 MHz, DMSO-d_6)

δ 7.89 (d, J = 1.8 Hz, =CH, 2H), 7.55 (d, J = 1.8 Hz, =CH, 2H), 6.69 (d, =CH, 2H), 3.87 (s, -CH_3, 6H) ppm. The single crystal suitable for X-ray diffraction analysis was obtained from slow diffusion of diethyl ether into an acetonitrile solution of **NHC-Ag** at room temperature.

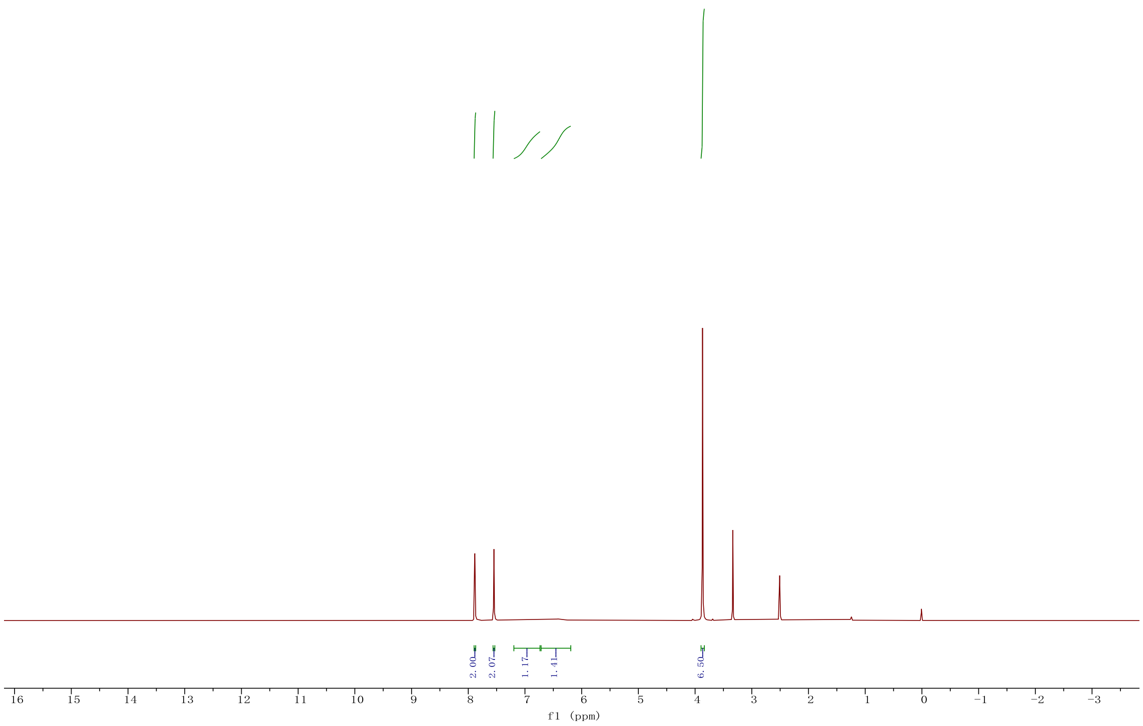

Figure S5-a. The ^{1}H NMR spectra of **NHC-Ag** in DMSO-d_6.

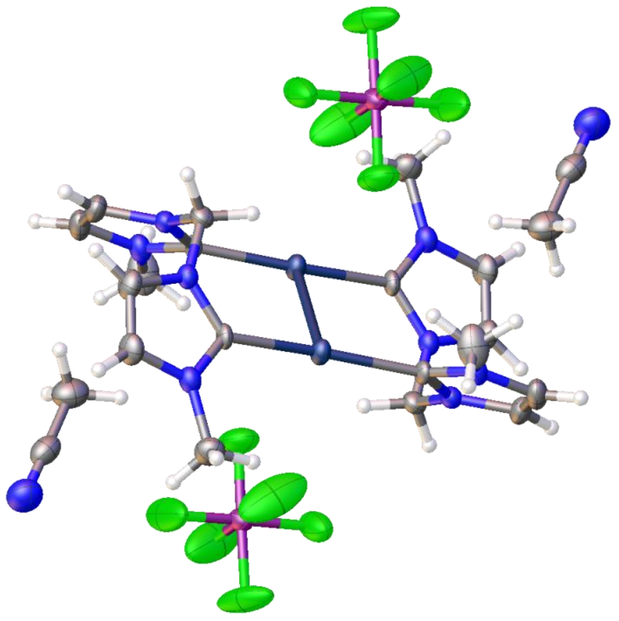

Figure S5-b. Crystal structure of **NHC-Ag**. (**CCDC 2343003**)

III. *N*-alkylation of Amines with Alcohols

1. GC analysis method for the condition optimization

1.1 GC analysis method

Injector: Mode: Split, Temp.: 330 °C, Gas: N_2 Pressure: 1.34 bar, Split ratio: 39:1, Split flow: 67.6 mL/min.

Column: Wondacap 1 column Capillary column (30 m × 0.25 mm), Nominal film thickness: 0.250 μm, Temperature program: Initial temperature 100 °C, heat to 120 °C with 5 °C/min, then heat to 200 °C with 50 °C/min, hold for 5 min.

Initial Flow: 1.62 mL/min, Average velocity: 39.4 cm/sec, Pressure: 1.34 bar. Detector (FID): Temp.: 330 °C, Hydrogen flow: 40.0 mL/min, Air flow: 400.0 mL/min. Preparation of GC sample:

Dilute the crude reaction mixture with 2 mL CH_3OH, 5 mL EtOAc, and 113 uL dodecane in order, filtered through the syringe filter, and collected in GC vials for analysis.

Retention times: Benzaldehyde: 2.79 min, Aniline: 2.87 min, Benzyl alcohol: 3.33 min, *N*-1-diphenylmethanimine: 8.66 min, *N, N*-benzyl phenylamine: 9.05 min.

2. The *N*-alkylation of amines with alcohols by silver complex

2.1 The general method for screening the reaction conditions

To a 10 mL seal tube charged with a stirring bar in a glovebox, was added aniline (**2a**), benzyl alcohol (**1a**), base, Ag catalyst, and 1 mL solvent. Then the tube was sealed with a Teflon plug and removed from the glovebox. The reaction mixture was stirred for 24 h at 150 ºC. After cooling to R.T., the crude reaction mixture was diluted with 2 mL CH_3OH, 5 mL EtOAc, and 113 uL dodecane in order, filtered through a syringe filter, and collected in GC vials for analysis.

Table S1. Optimization of reaction conditions

Entry	**1a**/**2a**	**Cat.** (mol%)	T (°C)	Base (eq.)	Solvent (1 mL)	Time (h)	**3a** Yield (%)
1	2.0	5.0	150	KO^{t}Bu (0.7)	1,4-dioxane	24	95
2	2.0	Ag_2O (2.5) + **L1** (10.0)	150	KO^{t}Bu (0.7)	1,4-dioxane	24	30
3	1.5	5.0	150	KO^{t}Bu (0.5)	1,4-dioxane	24	78
4	1.5	5.0	150	NaO^{t}Bu (0.5)	1,4-dioxane	24	72
5	1.5	5.0	150	Cs_2CO_3 (0.5)	1,4-dioxane	24	trace
6	1.5	5.0	150	KOH (0.5)	1,4-dioxane	24	trace
7		5.0	150	KO^{t}Bu (0.5)	neat	24	16
8	1.5	5.0	150	KO^{t}Bu (0.5)	toluene	24	68
9	1.5	5.0	150	KO^{t}Bu (0.5)	xylene	24	65
10	2.0	5.0	150	KO^{t}Bu (0.5)	1,4-dioxane	24	89
11	3.0	5.0	150	KO^{t}Bu (0.5)	1,4-dioxane	24	85
12	1.5	5.0	150	KO^{t}Bu (0.7)	1,4-dioxane	24	89
13	1.5	5.0	150	KO^{t}Bu (0.9)	1,4-dioxane	24	47
14	2.0	2.5	150	KO^{t}Bu (0.7)	1,4-dioxane	24	94(90)
15	2.0	1.0	150	KO^{t}Bu (0.7)	1,4-dioxane	24	88
16	2.0	2.5	140	KO^{t}Bu (0.7)	1,4-dioxane	24	66
17	2.0	2.5	120	KO^{t}Bu (0.7)	1,4-dioxane	24	36
18	2.0	2.5	100	KO^{t}Bu (0.7)	1,4-dioxane	24	14
19	2.0		150	KO^{t}Bu (0.7)	1,4-dioxane	24	trace
20	2.0	Ag_2O (2.5)	150	KO^{t}Bu (0.7)	1,4-dioxane	24	trace
21	2.0	**L6** (5.0)	150	KO^{t}Bu (0.7)	1,4-dioxane	24	trace

22	2.0	Ag_2O (2.5) + **L6** (5.0)	150	KO^{t}Bu (0.7)	1,4-dioxane	24	74

2.2 The general method for the substrate screenings

To a 10 mL seal tube in a glovebox, added amines (**2**, 0.5 mmol), alcohols (**1**, 1.0 mmol), KO^{t}Bu (0.7 equiv.), **NHC-Ag** (2.5 mol%) and 1,4-dioxane (1 mL). Then the tube was sealed with a Teflon plug and removed from the glove box. The reaction mixture was stirred for 24 h at 150 ºC. After cooling to R.T., the crude reaction mixture was diluted with 2 mL CH_3OH and 5 mL EtOAc in order. The solvent was evaporated to dryness and the corresponding amine was purified by column chromatography with silica gel. The yields were calculated based on isolated products.

Characterization data for products

N-benzyl-4-methoxyaniline (**3aa**).[6]

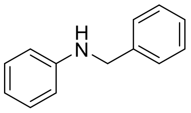

The compound was prepared as described in the general method (oil, 95 % isolated yield, 87 mg). ^{1}H NMR (400 MHz, Chloroform-*d*) δ 7.49 – 7.41 (m, -C_6H_5, 4H), 7.40 – 7.34 (m, -C_6H_5, 1H), 7.29 – 7.23 (t, -C_6H_5, 2H), 6.86 – 6.78 (t, -C_6H_5, 1H), 6.76 – 6.69 (d, -C_6H_5, 2H), 4.41 (s, -CH_2, 2H), 4.09 (s, -NH, 1H) ppm. ^{13}C NMR (101 MHz, Chloroform-*d*) δ 48.47, 112.98, 117.70, 127.37, 127.65, 128.77, 129.40, 139.58, 148.30 ppm.

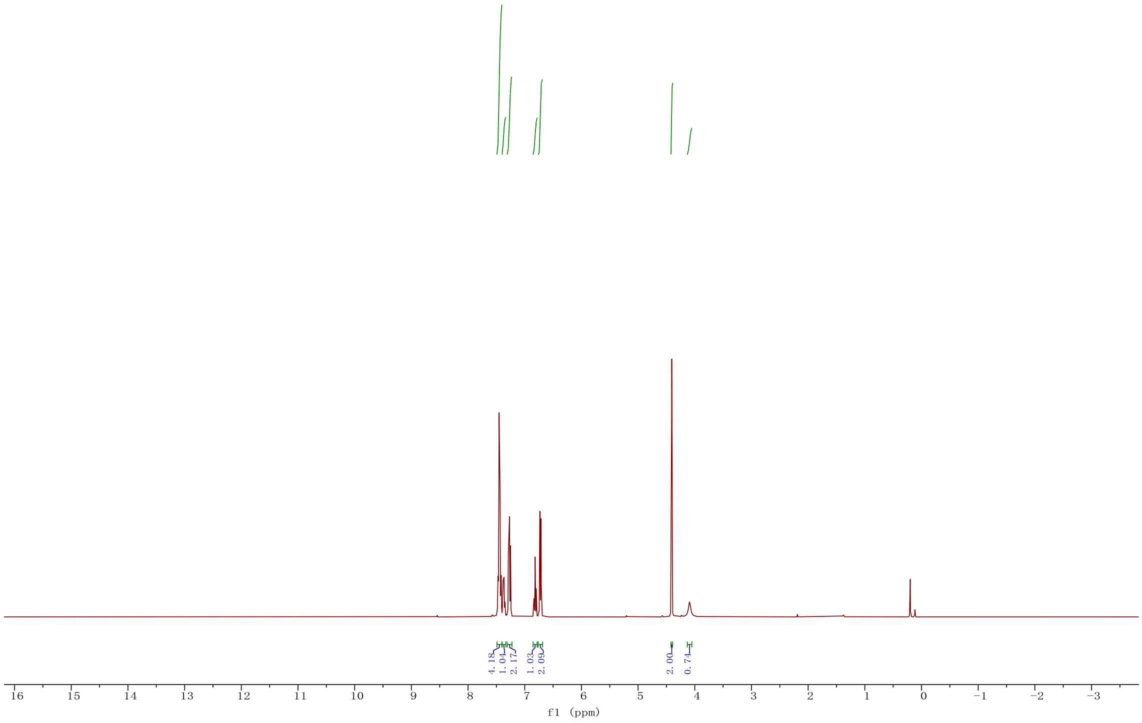

Figure S6-1a. The ^{1}H NMR spectra of **3aa** in Chloroform-*d*.

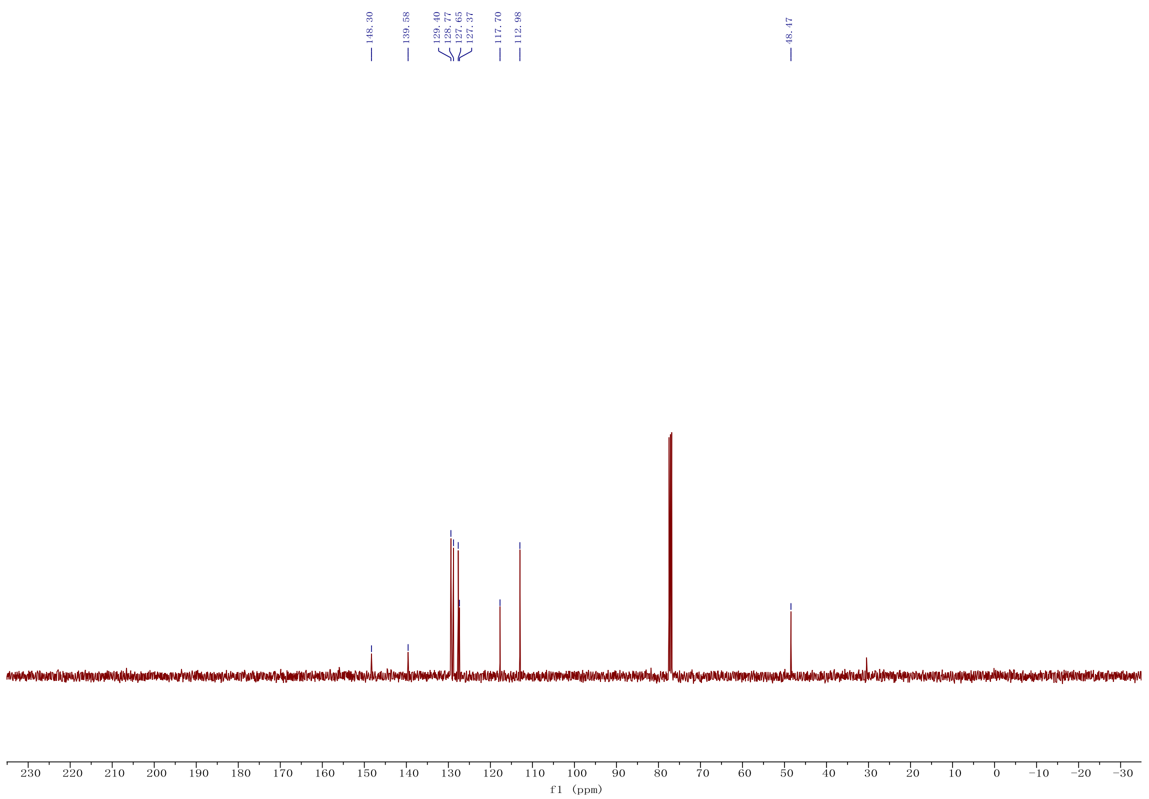

Figure S6-1b. The ^{13}C NMR spectra of **3aa** in Chloroform-*d*.

N-benzyl-4-methoxyaniline (**3ab**).[6]

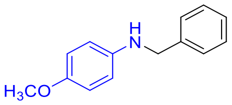

The compound was prepared as described in the general method (colorless oil, 92 % isolated yield, 98 mg). ^{1}H NMR (400 MHz, Chloroform-*d*) δ 7.45 – 7.33 (m, -C_6H_5, 4H), 7.33 – 7.26 (t, -C_6H_5, 1H), 6.85 – 6.77 (m, -C_6H_5, 2H), 6.68 – 6.60 (m, -C_6H_5, 2H), 4.32 (s, -CH_2, 2H), 3.77 (s, -OCH_3, 3H) ppm. ^{13}C NMR (101 MHz, Chloroform-*d*) δ = 49.26, 55.83, 114.13, 114.90, 127.21, 127.59, 128.63, 139.67, 142.43, 152.19 ppm.

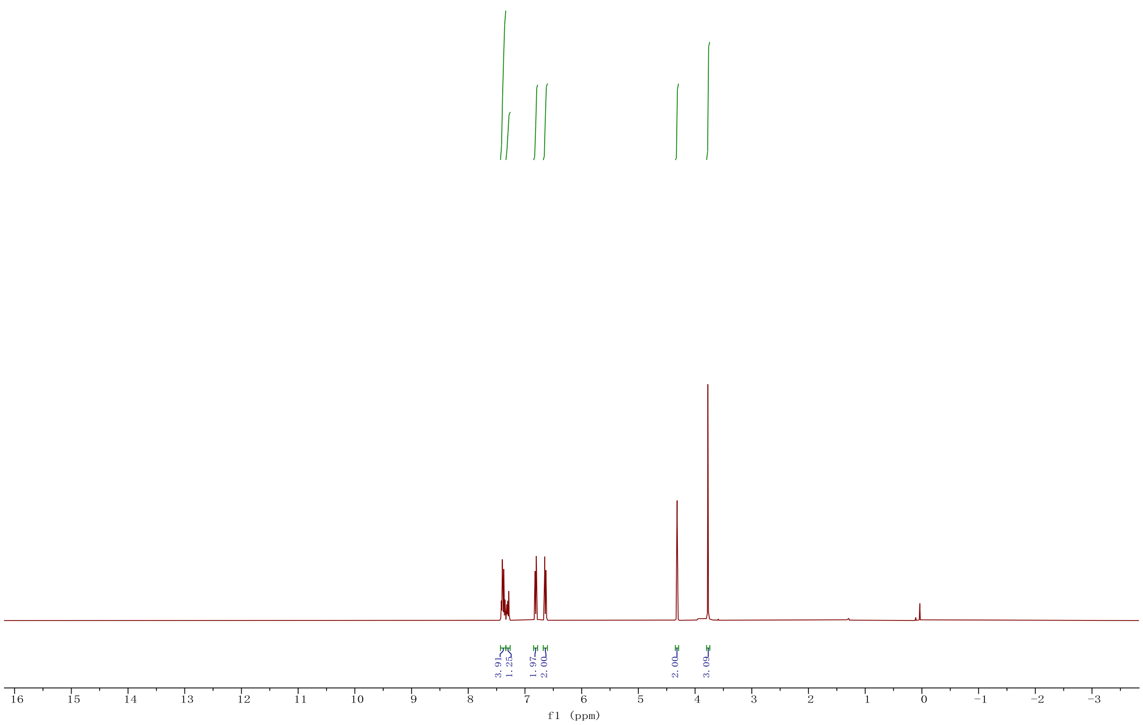

Figure S6-2a. The ^{1}H NMR spectra of **3ab** in Chloroform-*d*.

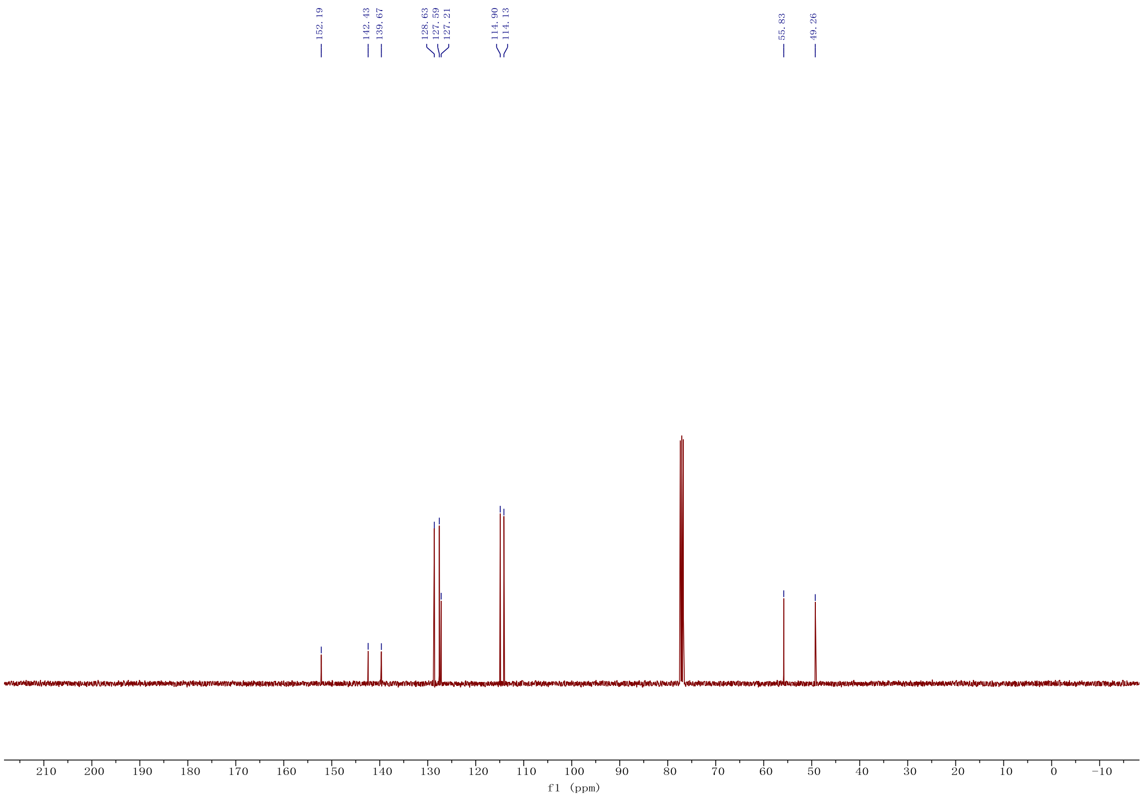

Figure S6-2b. The ^{13}C NMR spectra of **3ab** in Chloroform-*d*.

N-benzyl-3-methoxyaniline (**3ac**).[7]

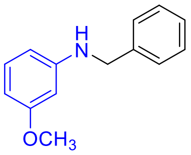

The compound was prepared as described in the general method (colorless oil, 83 % isolated yield, 88 mg). ^{1}H NMR (400 MHz, Chloroform-*d*) δ 7.47 – 7.38 (m, -C_6H_5, 4H), 7.38 – 7.30 (m, -C_6H_5, 1H), 7.15 (t, *J* = 8.1 Hz, -C_6H_5, 1H), 6.35 (m, -C_6H_5, 2H), 6.27 (t, *J* = 2.3 Hz, -C_6H_5, 1H), 4.37 (s, -CH_2, 2H), 4.10 (s, -NH, 1H), 3.81 (s, -OCH_3, 3H) ppm. ^{13}C NMR (101 MHz, Chloroform-*d*) δ = 48.38, 55.11, 98.99, 102.79, 106.07, 127.29, 127.58, 128.69, 130.06, 139.45, 149.66, 160.93 ppm.

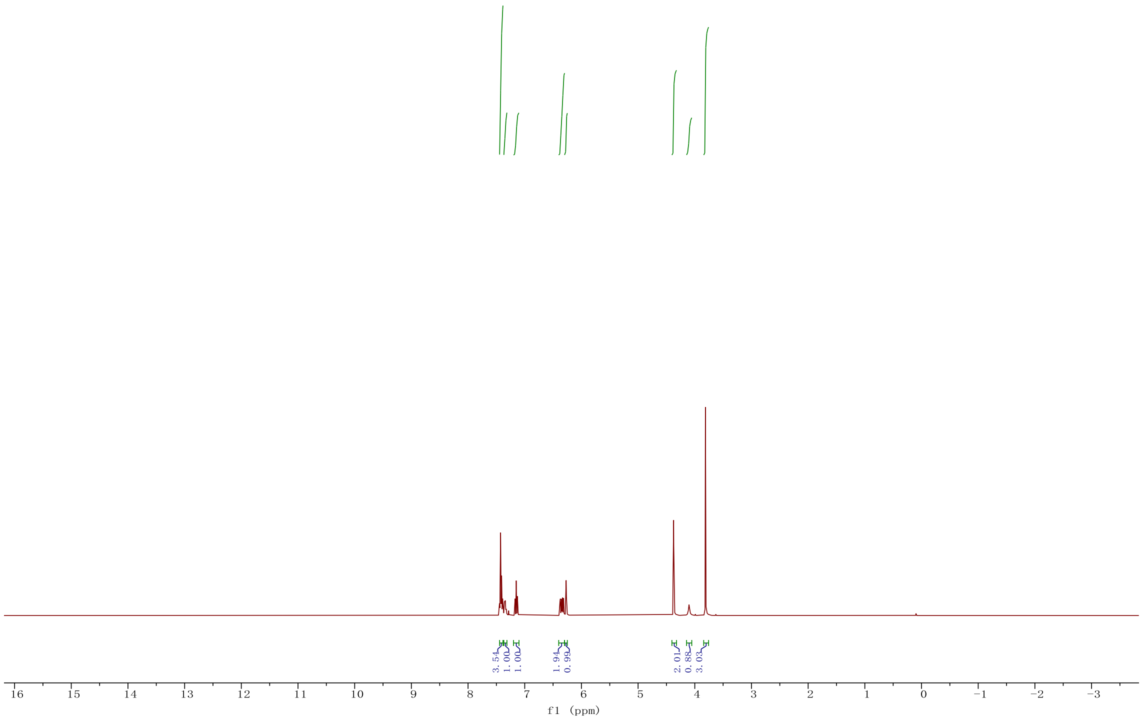

Figure S6-3a. The ^{1}H NMR spectra of **3ac** in Chloroform-*d*.

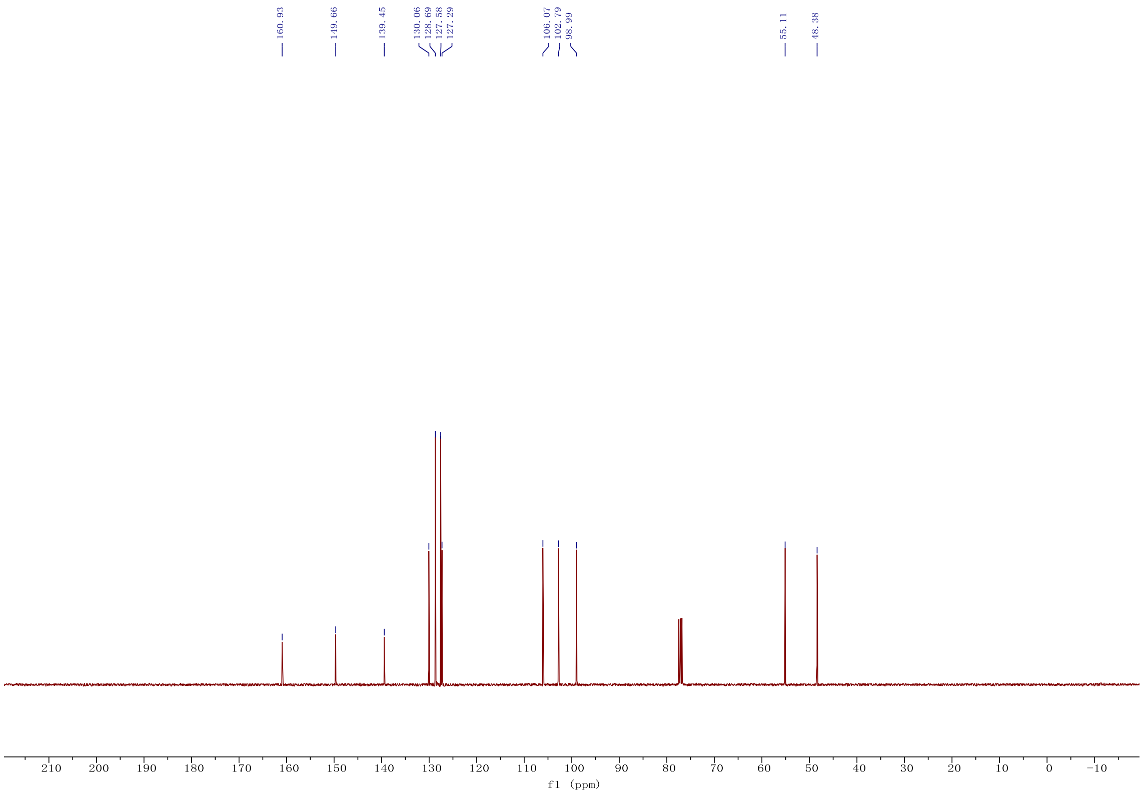

Figure S6-3b. The ^{13}C NMR spectra of **3ac** in Chloroform-*d*.

N-benzyl-2-methoxyaniline (**3ad**).[8]

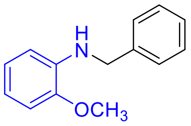

The compound was prepared as described in the general method (oil, 87 % isolated yield, 93 mg). ^{1}H NMR (400 MHz, Chloroform-*d*) δ 7.49 – 7.37 (m, -C_6H_5, 4H), 7.37 – 7.30 (m, -C_6H_5, 1H), 6.91 – 6.83 (m, -C_6H_5, 2H), 6.75 (m, -C_6H_5, 1H), 6.67 (m, -C_6H_5, 1H), 4.70 (s, -NH, 1H), 4.42 (s, -CH_2, 2H), 3.91 (s, -OCH_3, 3H) ppm. ^{13}C NMR (101 MHz, Chloroform-*d*) δ = 48.06, 55.45, 109.41, 110.12, 116.69, 121.33, 127.19, 127.58, 128.65, 138.17, 139.64, 146.83 ppm.

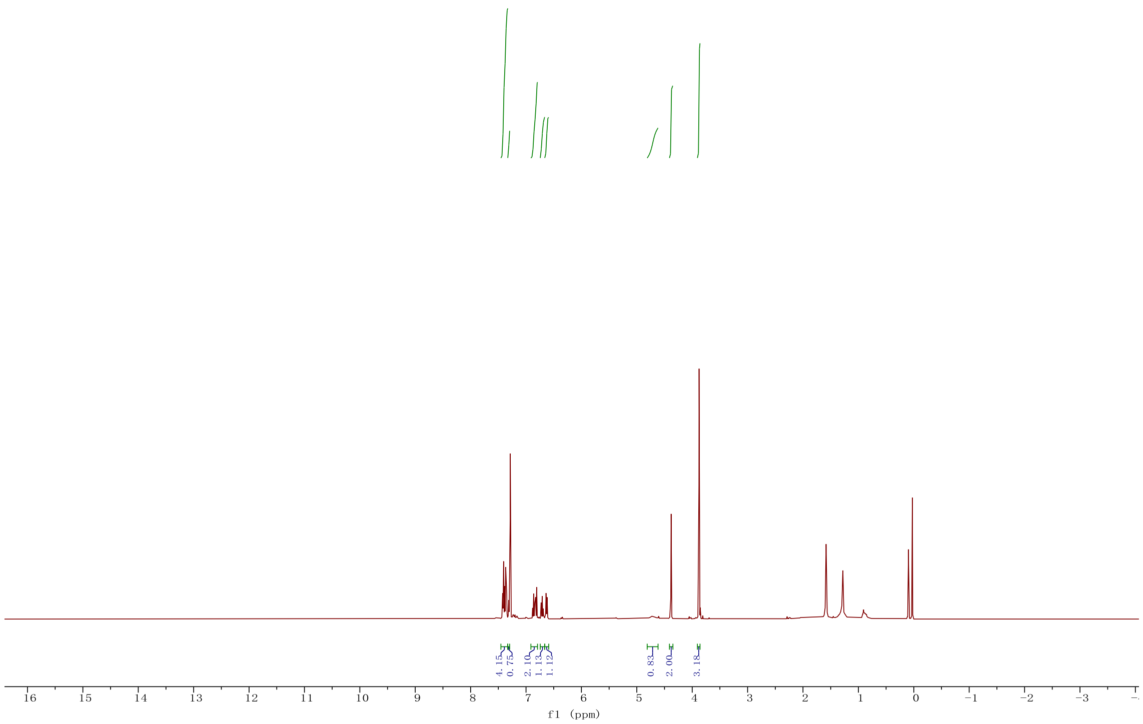

Figure S6-4a. The ^{1}H NMR spectra of **3ad** in Chloroform-*d*.

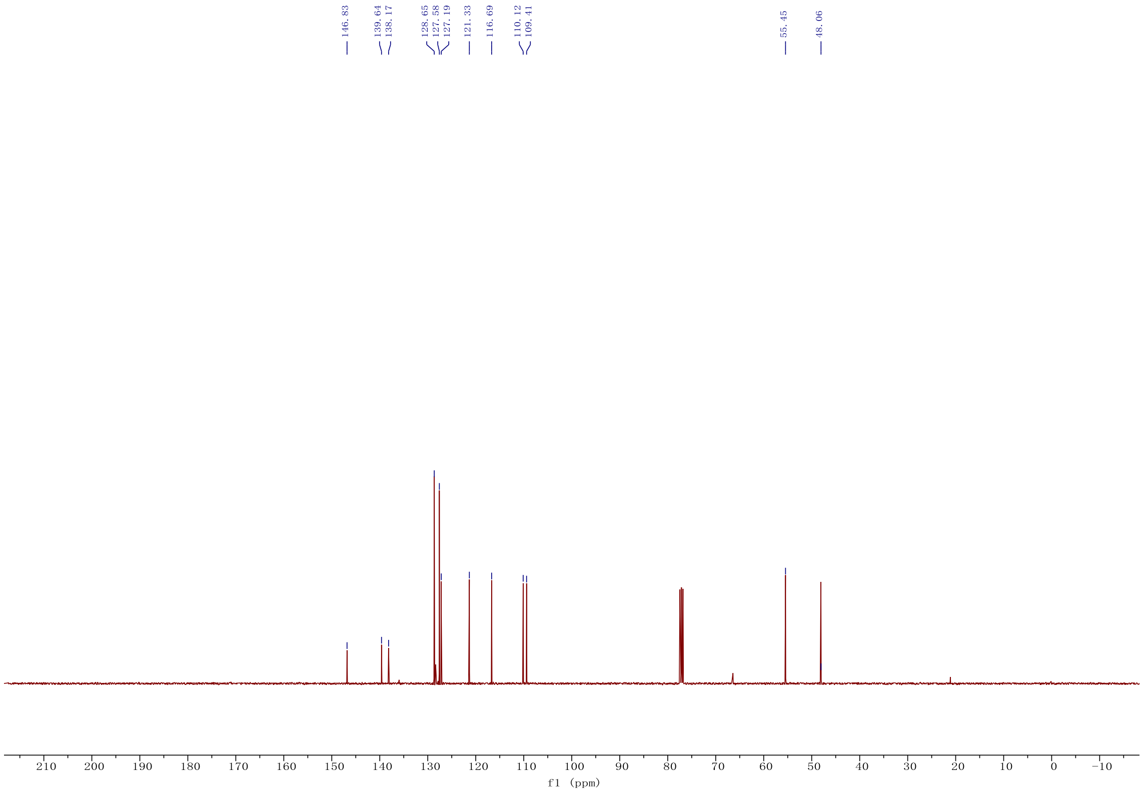

Figure S6-4b. The ^{13}C NMR spectra of **3ad** in Chloroform-*d*.

N-benzyl-4-methylaniline (**3ae**).[6]

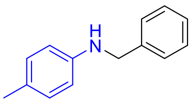

The compound was prepared as described in the general method (oil, 86 % isolated yield, 85 mg). ^{1}H NMR (400 MHz, Chloroform-*d*) δ 7.46 – 7.38 (m, -C_6H_5, 4H), 7.36 – 7.29 (t, -C_6H_5, 1H), 7.05 (d, *J* = 8.3 Hz, -C_6H_5, 2H), 6.66 – 6.59 (d, -C_6H_5, 2H), 4.37 (s, -CH_2, 2H), 3.96 (s, -NH, 1H), 2.30 (s, -CH_3, 3H) ppm. ^{13}C NMR (101 MHz, Chloroform-*d*) δ = 20.48, 48.67, 113.09, 126.79, 127.22, 127.56, 128.66, 129.81, 139.71, 145.97 ppm.

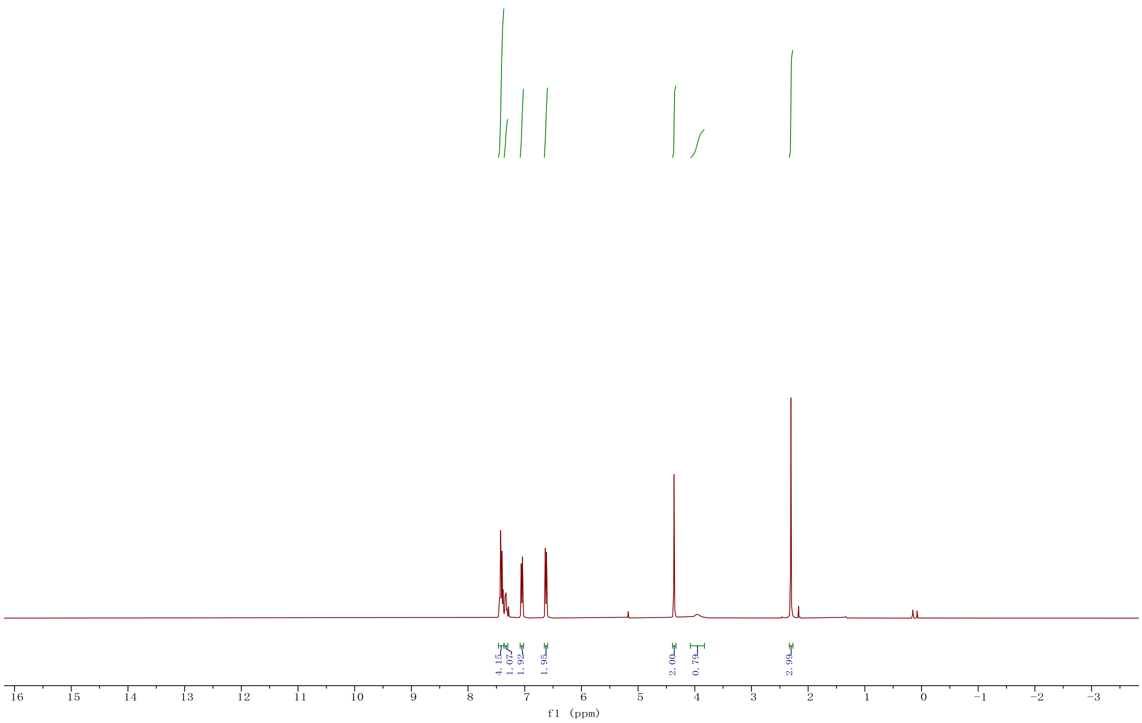

Figure S6-5a. The ^{1}H NMR spectra of **3ae** in Chloroform-*d*.

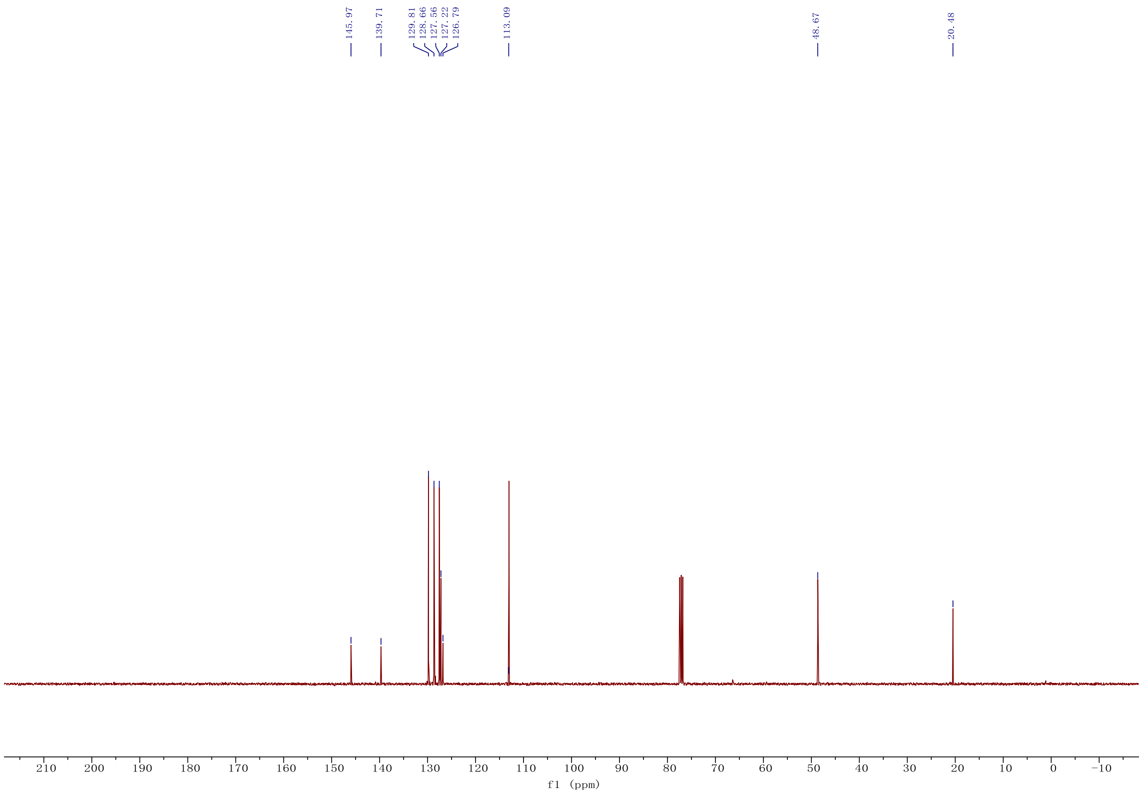

Figure S6-5b. The ^{13}C NMR spectra of **3ae** in Chloroform-*d*.

N-benzyl-3-methylaniline (**3af**).[8]

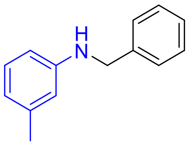

The compound was prepared as described in the general method (oil, 76 % isolated yield, 75 mg). ^{1}H NMR (400 MHz, Chloroform-*d*) δ 7.43 – 7.34 (m, -C_6H_5, 4H), 7.33 – 7.29 (m, -C_6H_5, 1H), 7.10 (t, J = 7.7 Hz, -C_6H_5, 1H), 6.58 (d, J = 7.5 Hz, -C_6H_5, 1H), 6.53 – 6.46 (m, -C_6H_5, 2H), 4.35 (s, -CH_2, 2H), 4.00 (s, -NH, 1H), 2.30 (s, -CH_3, 3H) ppm. ^{13}C NMR (101 MHz, Chloroform-*d*) δ = 21.67, 48.36, 109.95, 113.62, 118.54, 127.23, 127.56, 128.65, 129.18, 139.09, 139.55, 148.23 ppm.

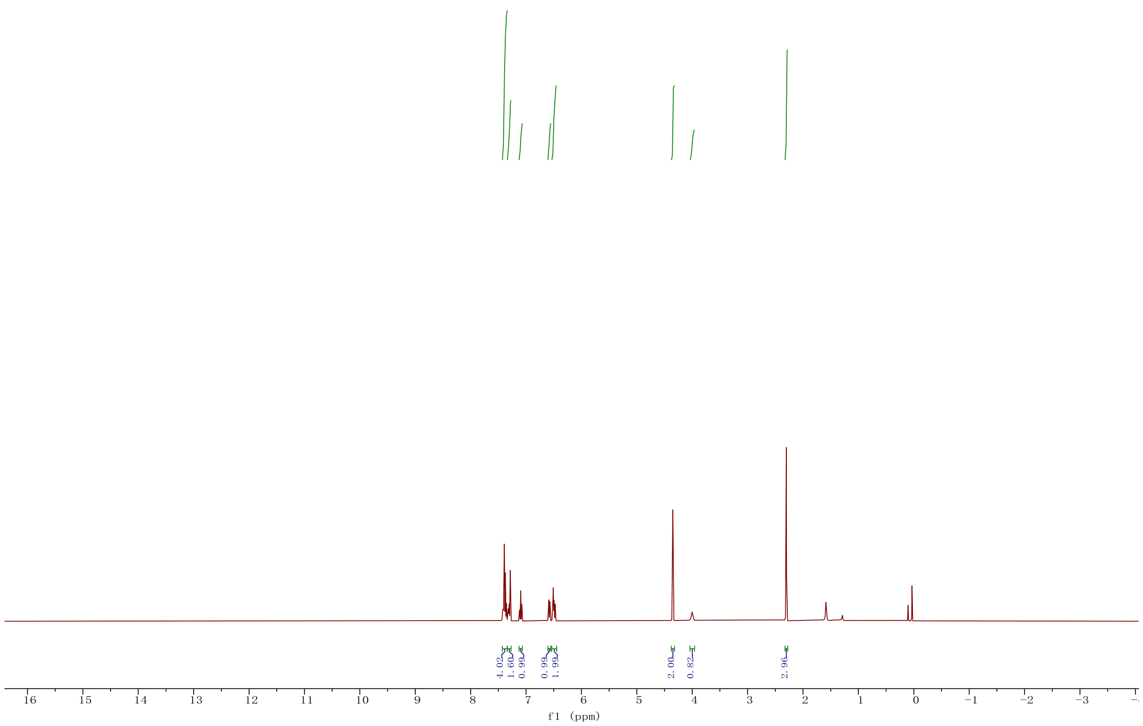

Figure S6-6a. The ^{1}H NMR spectra of **3af** in Chloroform-*d*.

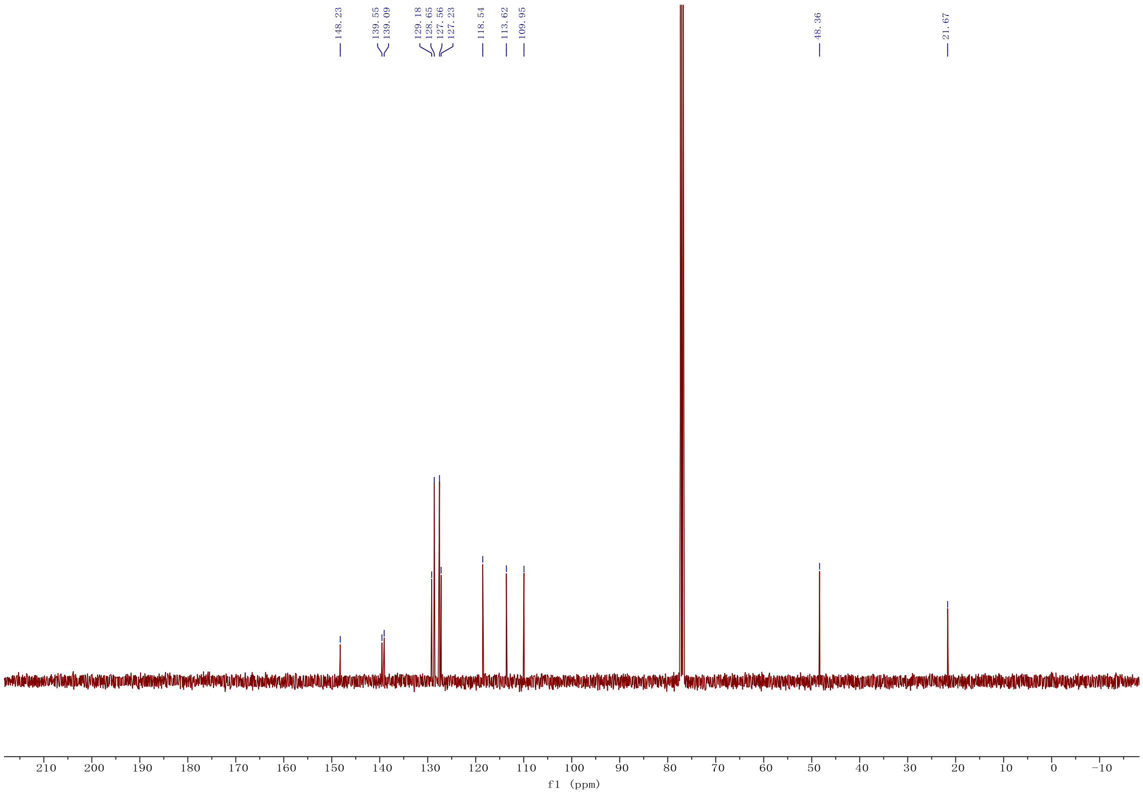

Figure S6-6b. The ^{13}C NMR spectra of **3af** in Chloroform-*d*.

N-benzyl-2-methylaniline (**3ag**).[6]

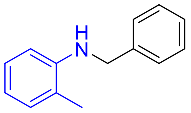

The compound was prepared as described in the general method (oil, 82 % isolated yield, 81 mg). ^{1}H NMR (400 MHz, Chloroform-*d*) δ 7.46 – 7.37 (m, -C_6H_5, 4H), 7.36 – 7.30 (m, -C_6H_5, 1H), 7.19 – 7.09 (m, -C_6H_5, 2H), 6.72 (m, -C_6H_5, 1H), 6.66 (d, J = 8.0 Hz, -C_6H_5, 1H), 4.42 (s, -CH_2, 2H), 3.91 (s, -NH, 1H), 2.21 (s, -CH_3, 3H) ppm. ^{13}C NMR (101 MHz, Chloroform-*d*) δ = 17.63, 48.33, 109.98, 117.20, 121.96, 127.20, 127.30, 127.59, 128.71, 130.10, 139.51, 146.08 ppm.

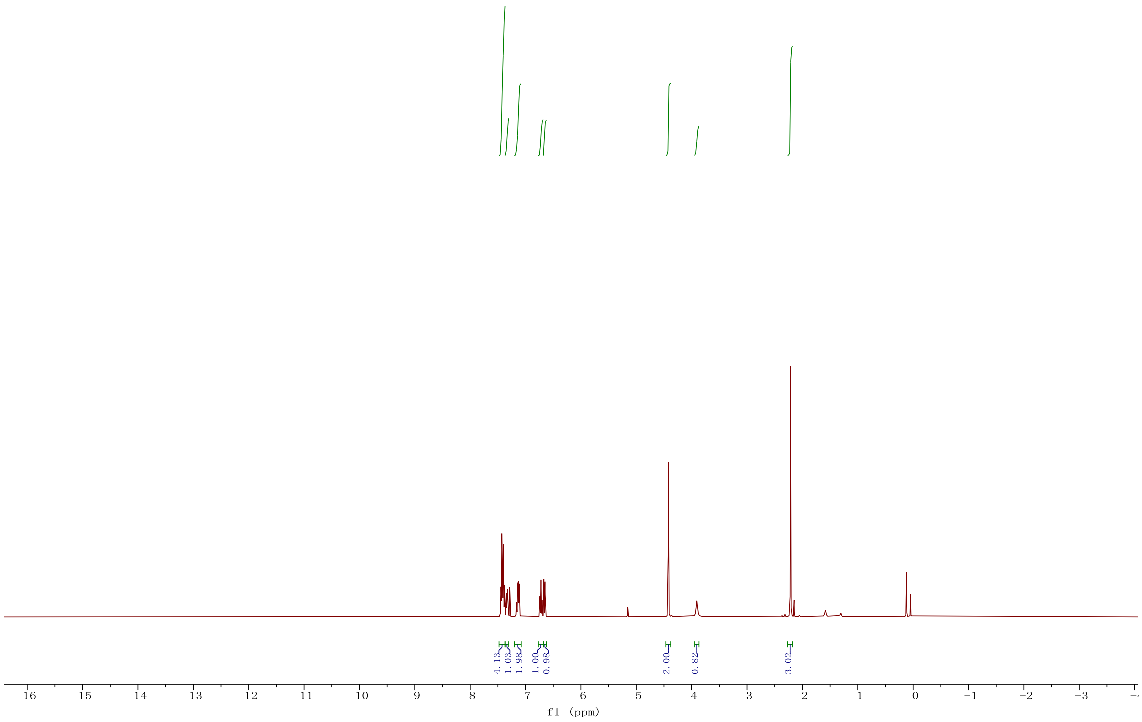

Figure S6-7a. The ^{1}H NMR spectra of **3ag** in Chloroform-*d*.

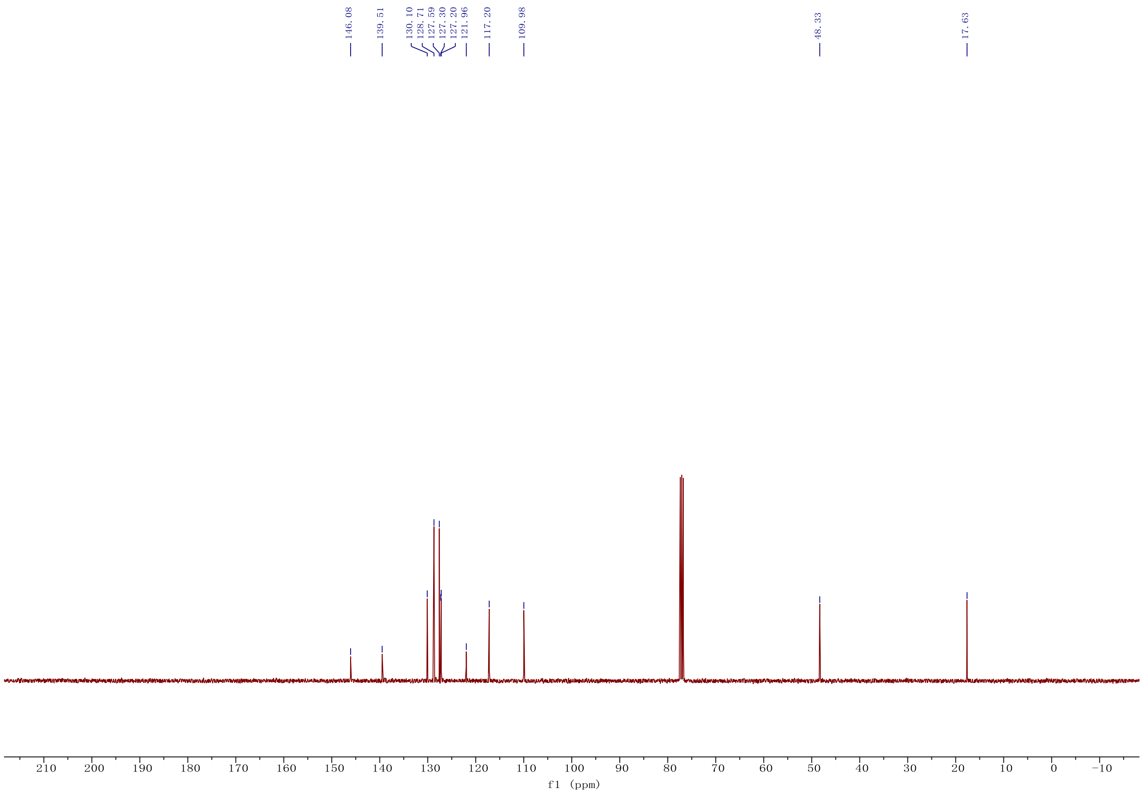

Figure S6-7b. The ^{13}C NMR spectra of **3ag** in Chloroform-*d*.

N-benzyl-4-(tert-butyl)aniline (**3ah**).[9]

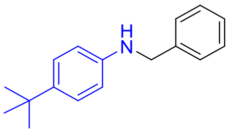

The compound was prepared as described in the general method (oil, 98 % isolated yield, 117 mg). ^{1}H NMR (400 MHz, Chloroform-*d*) δ 7.49 – 7.38 (m, -C_6H_5, 4H), 7.38 – 7.34 (t, -C_6H_5, 1H), 7.32 – 7.26 (m, -C_6H_5, 2H), 6.71 – 6.65 (m, -C_6H_5, 2H), 4.38 (s, -CH_2, 2H), 4.00 (s, -NH, 1H), 1.36 (s, -$C(CH_3)_3$, 9H) ppm. ^{13}C NMR (101 MHz, Chloroform-*d*) δ = 31.60, 33.88, 48.70, 112.64, 126.07, 127.22, 127.61, 128.64, 139.79, 140.40, 145.94 ppm.

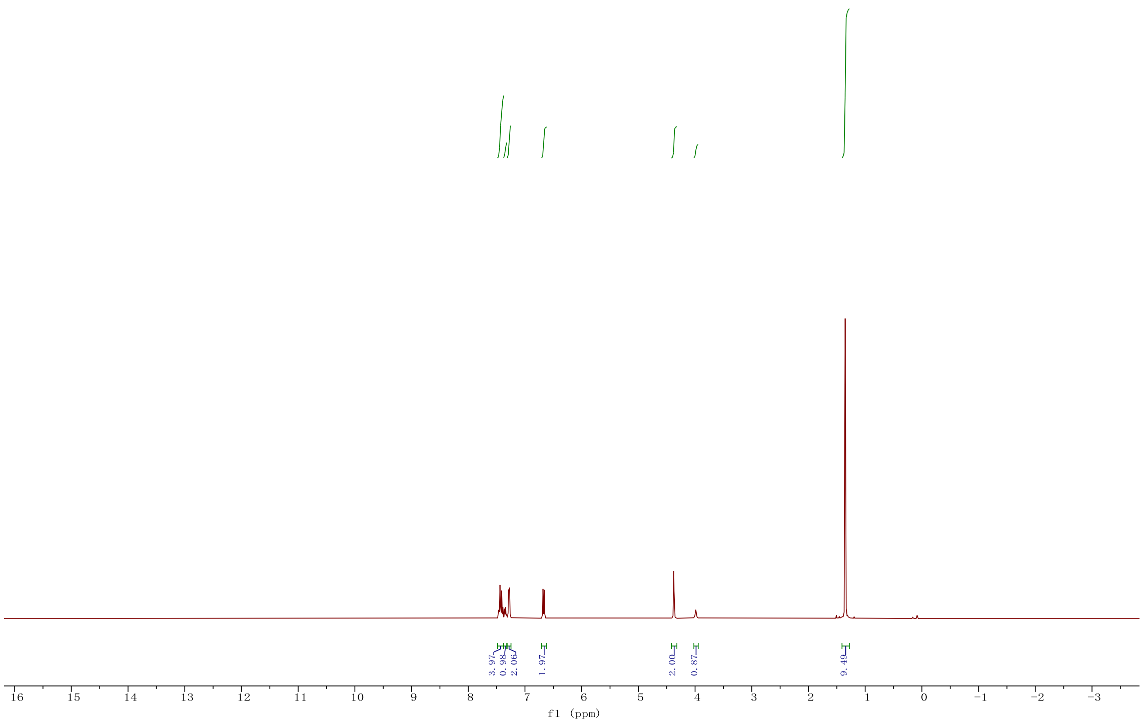

Figure S6-8a. The ^{1}H NMR spectra of **3ah** in Chloroform-*d*.

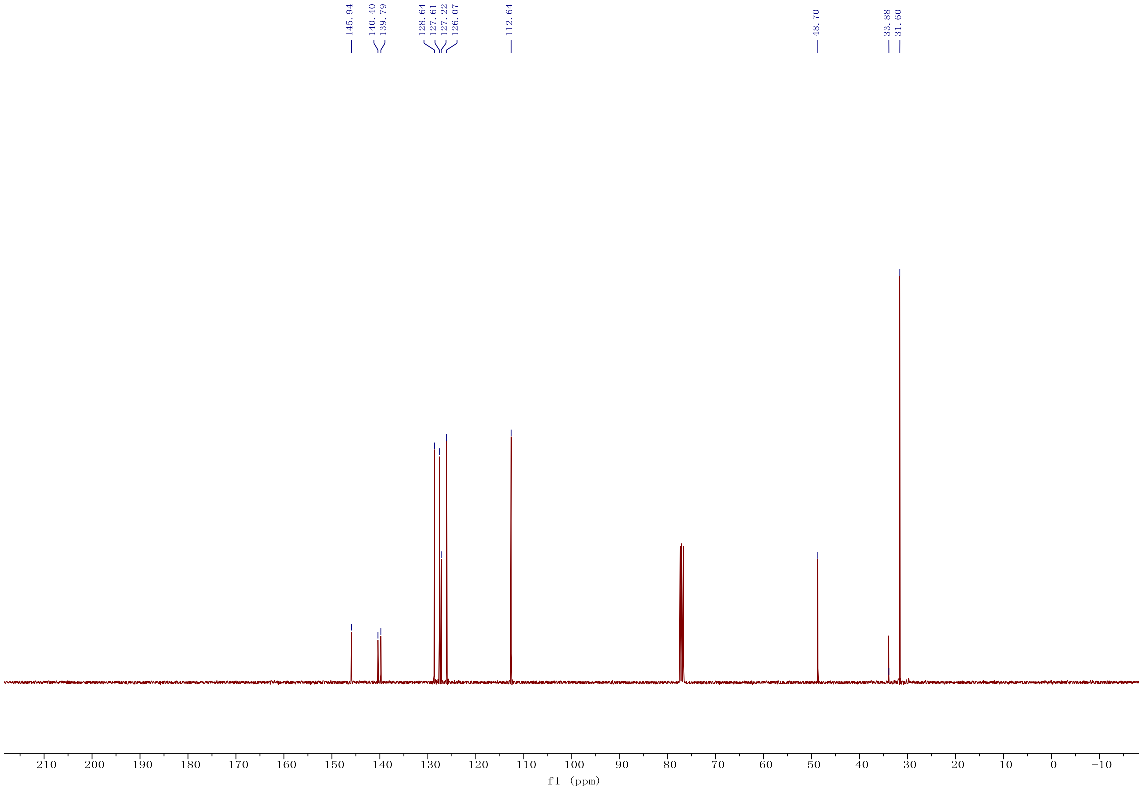

Figure S6-8b. The ^{13}C NMR spectra of **3ah** in Chloroform-*d*.

N-benzyl-2-(tert-butyl)aniline (**3ai**).[6]

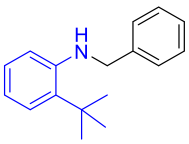

The compound was prepared as described in the general method (oil, 60 % isolated yield, 72 mg). ^{1}H NMR (400 MHz, Chloroform-*d*) δ 7.42 – 7.27 (m, -C_6H_5, 6H), 7.10 (t, J = 7.6 Hz, -C_6H_5, 1H), 6.75 – 6.63 (m, -C_6H_5, 2H), 4.41 (s, -CH_2, 2H), 4.28 (s, -NH, 1H), 1.44 (s, -$C(CH_3)_3$, 9H) ppm. ^{13}C NMR (101 MHz, Chloroform-*d*) δ 29.98, 34.22, 48.88, 111.96, 117.26, 126.23, 127.22, 127.52, 128.74, 133.29, 139.68, 146.17 ppm.

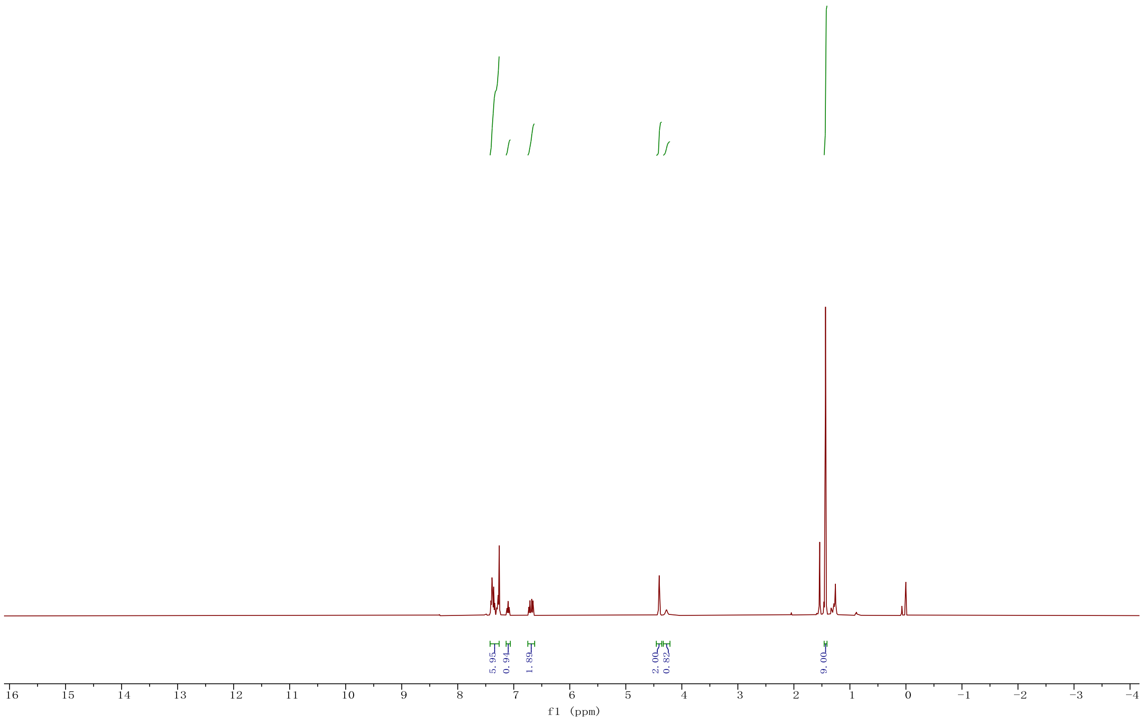

Figure S6-9a. The ^{1}H NMR spectra of **3ai** in Chloroform-*d*.

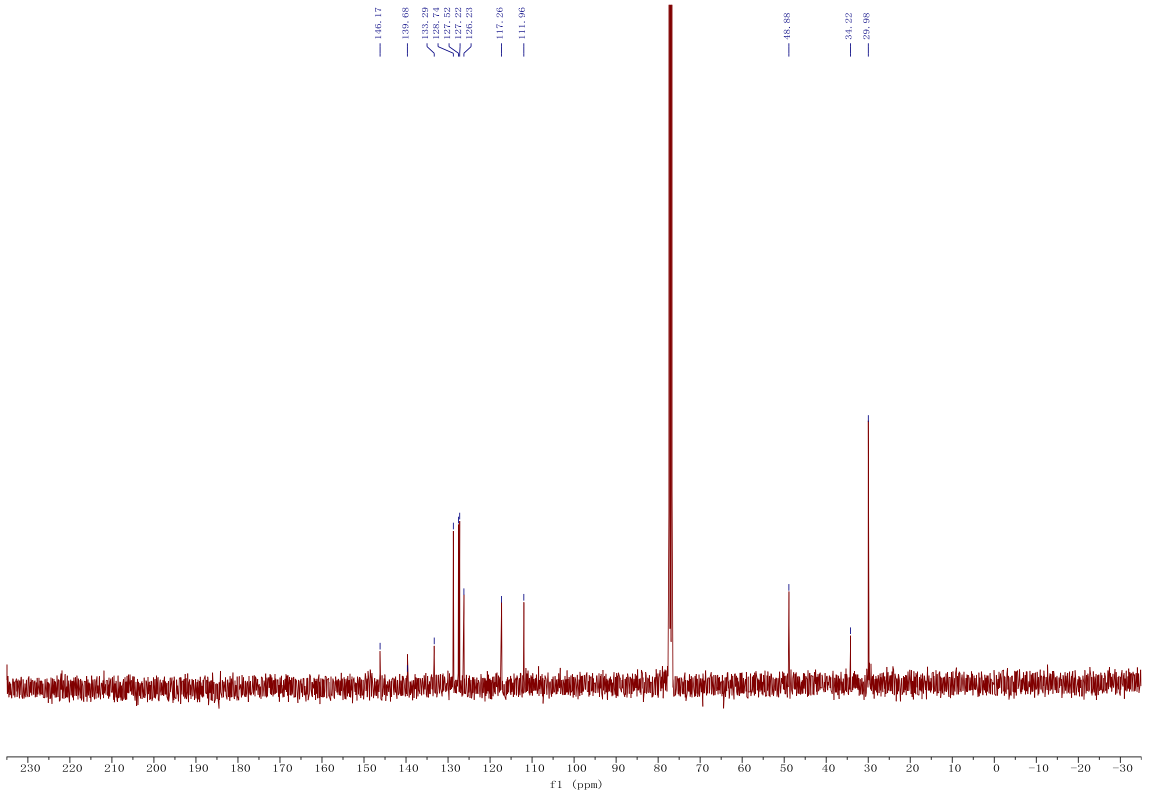

Figure S6-9b. The ^{13}C NMR spectra of **3ai** in Chloroform-*d*.

N-benzyl-3-vinylaniline (**3aj**).[6]

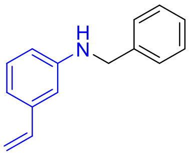

The compound was prepared as described in the general method (oil, 64 % isolated yield, 67 mg). ^{1}H NMR (400 MHz, Chloroform-*d*) δ 7.41 – 7.27 (m, -C_6H_5, 5H), 7.12 (t, -C_6H_5, 1H), 6.79 (d, J = 7.5 Hz, =CH, 1H), 6.71 – 6.50 (m, -C_6H_5, 3H), 5.67 (d, J = 17.5 Hz, =CH_2, 1H), 5.19 (d, J = 10.9 Hz, =CH_2, 1H), 4.34 (s, -CH_2, 2H), 4.03 (s, -NH, 1H). ^{13}C NMR (101 MHz, Chloroform-*d*) δ 48.42, 110.21, 110.65, 112.59, 113.55, 115.94, 117.40, 127.33, 127.59, 128.71, 129.45, 137.33, 138.64, 139.43, 148.40 ppm.

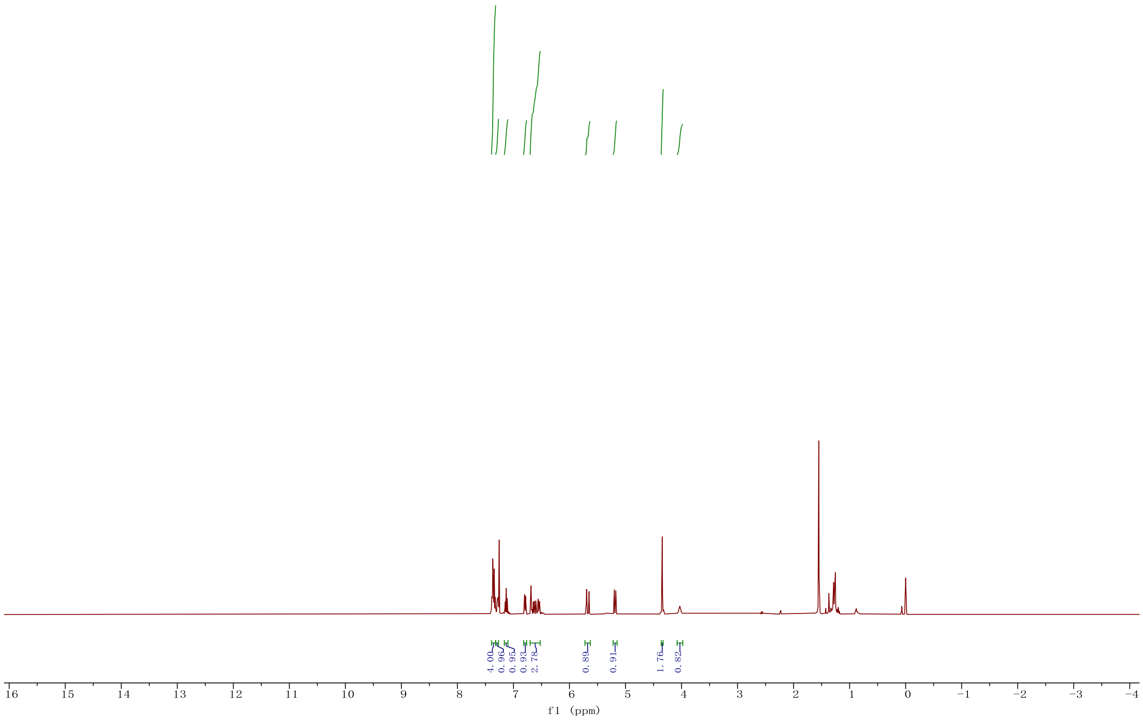

Figure S6-10a. The ^{1}H NMR spectra of **3aj** in Chloroform-*d*.

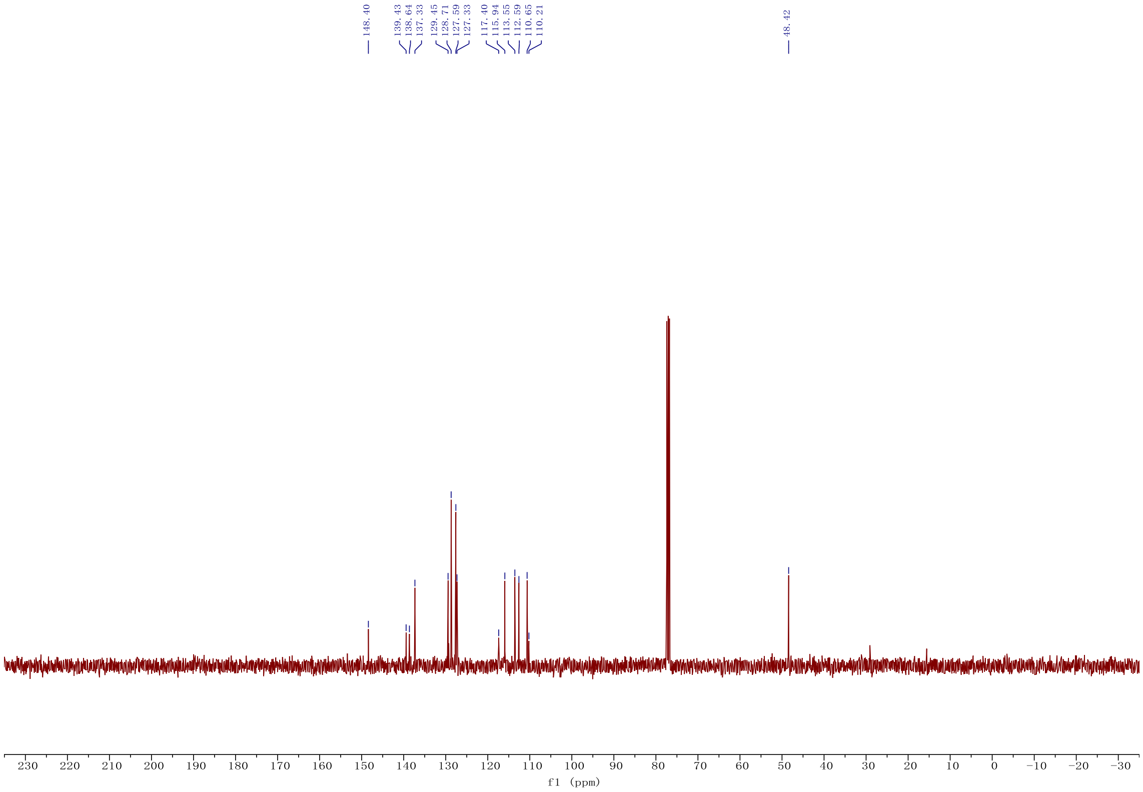

Figure S6-10b. The ^{13}C NMR spectra of **3aj** in Chloroform-*d*.

N-benzyl-4-(methylthio)aniline (**3ak**).[6]

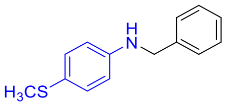

The compound was prepared as described in the general method (oil, 83 % isolated yield, 96 mg). ^{1}H NMR (400 MHz, Chloroform-*d*) δ 7.42 – 7.28 (m, $-C_6H_5$, 5H), 7.28 – 7.20 (m, $-C_6H_5$, 2H), 6.65 – 6.58 (m, $-C_6H_5$, 2H), 4.34 (s, $-CH_2$, 2H), 4.17 (s, -NH, 1H), 2.43 (s, $-SCH_3$, 3H) ppm. ^{13}C NMR (101 MHz, Chloroform-*d*) δ = 21.91, 48.33, 110.84, 113.51, 127.35, 127.49, 128.70, 131.47, 139.07, 147.87 ppm.

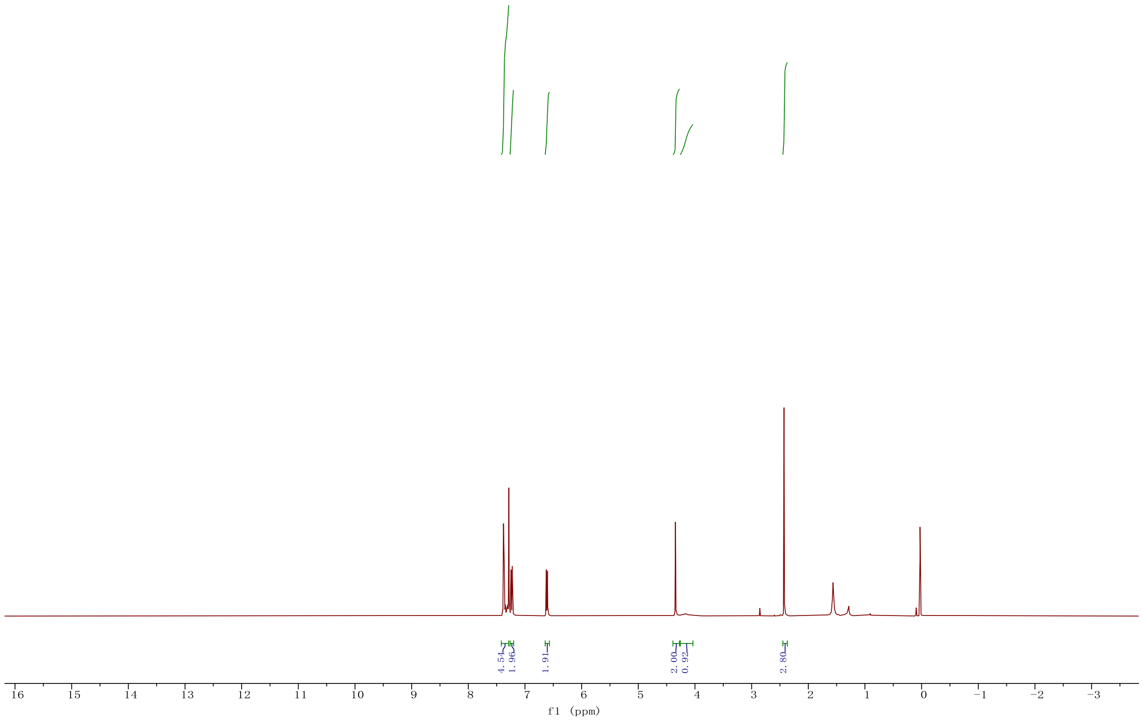

Figure S6-11a. The ^{1}H NMR spectra of **3ak** in Chloroform-*d*.

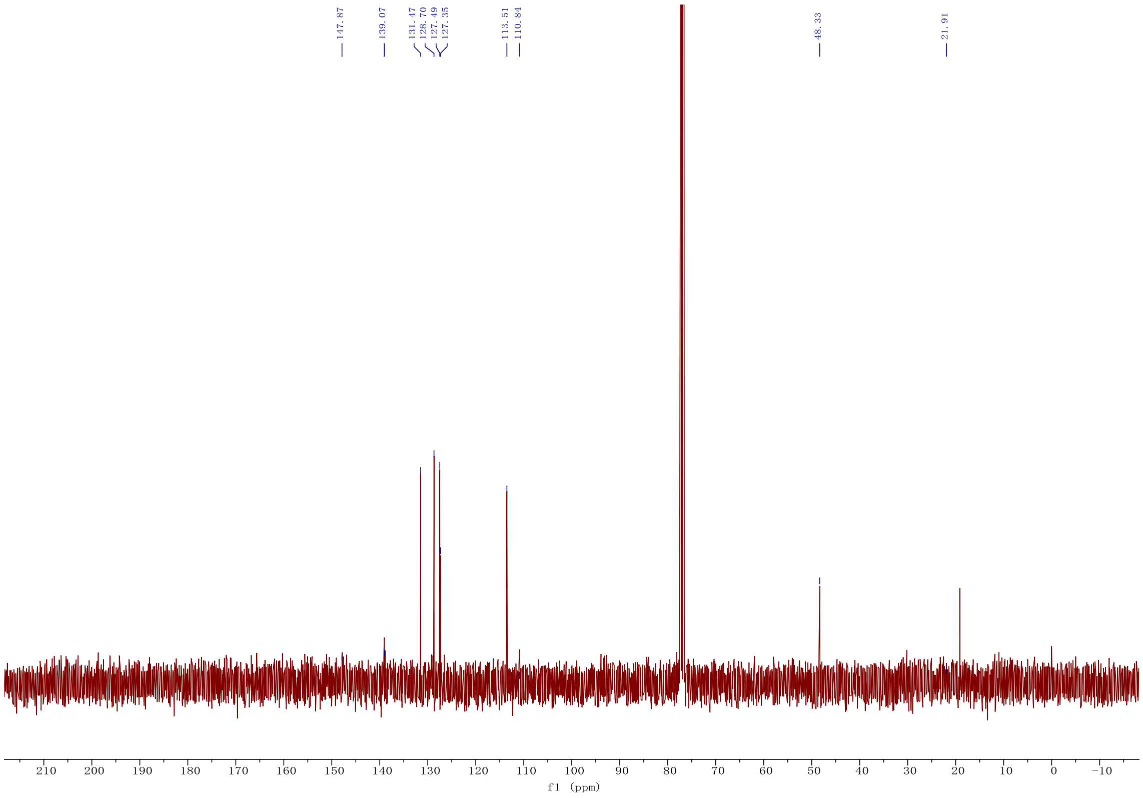

Figure S6-11b. The ^{13}C NMR spectra of **3ak** in Chloroform-*d*.

N-benzyl-3-(trifluoromethyl)aniline (**3al**).[8]

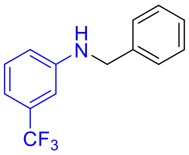

The compound was prepared as described in the general method (oil, 86 % isolated yield, 108 mg). ^{1}H NMR (400 MHz, Chloroform-*d*) δ 7.42 – 7.23 (m, -C_6H_5, 6H), 6.98 (d, J = 7.6 Hz, -C_6H_5, 1H), 6.88 (s, -C_6H_5, 1H), 6.78 (m, -C_6H_5, 1H), 4.38 (s, -CH_2, 2H), 4.24 (s, -NH, 1H) ppm. ^{13}C NMR (101 MHz, Chloroform-*d*) δ = 48.16, 109.08, 113.95, 115.72, 127.52, 128.78, 129.66, 138.59, 148.24 ppm.

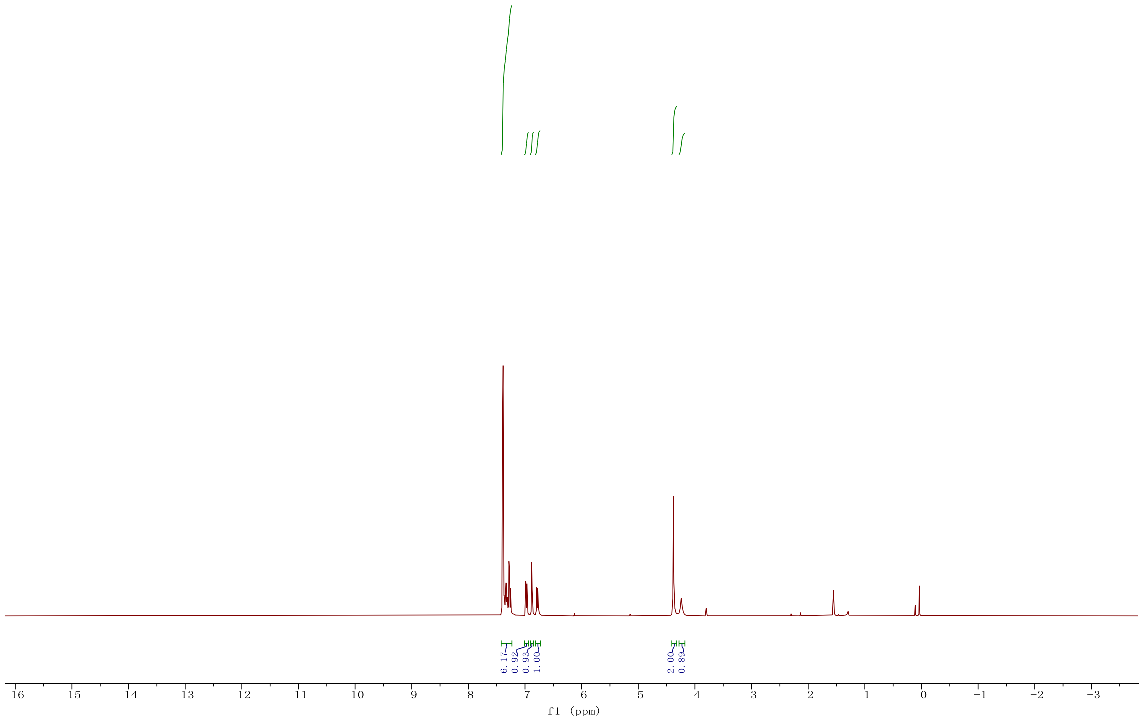

Figure S6-12a. The ^{1}H NMR spectra of **3al** in Chloroform-*d*.

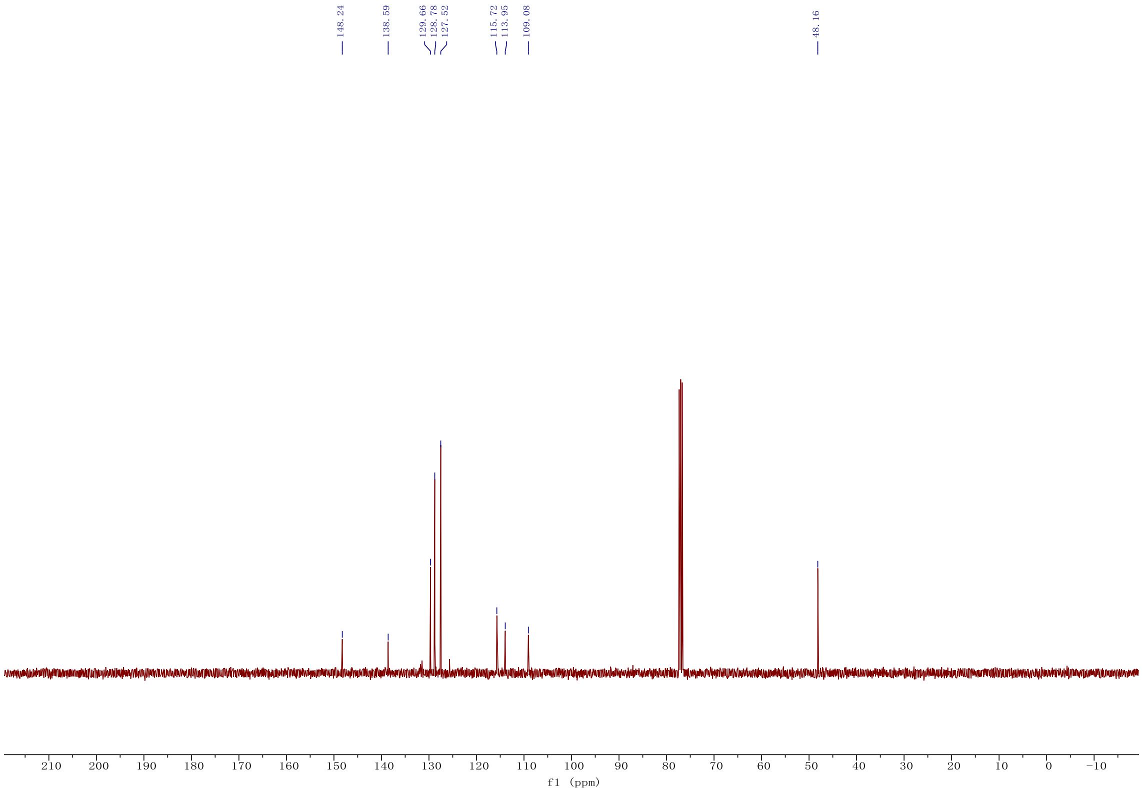

Figure S6-12b. The ^{13}C NMR spectra of **3al** in Chloroform-*d*.

N^1, N^2-dibenzylbenzene-1,2-diamine (**3am**).[10]

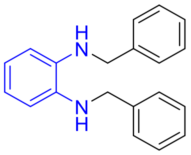

The compound was prepared as described in the general method (oil, 64 % isolated yield, 92 mg). ^{1}H NMR (400 MHz, Chloroform-*d*) δ 7.48 – 7.30 (m, -C_6H_5, 10H), 6.90 – 6.74 (m, -C_6H_5, 4H), 4.38 (s, -CH_2, 4H), 3.74 (s, -NH, 2H) ppm. ^{13}C NMR (101 MHz, Chloroform-*d*) δ = 48.87, 112.89, 119.52, 127.27, 127.84, 128.62, 137.97 ppm.

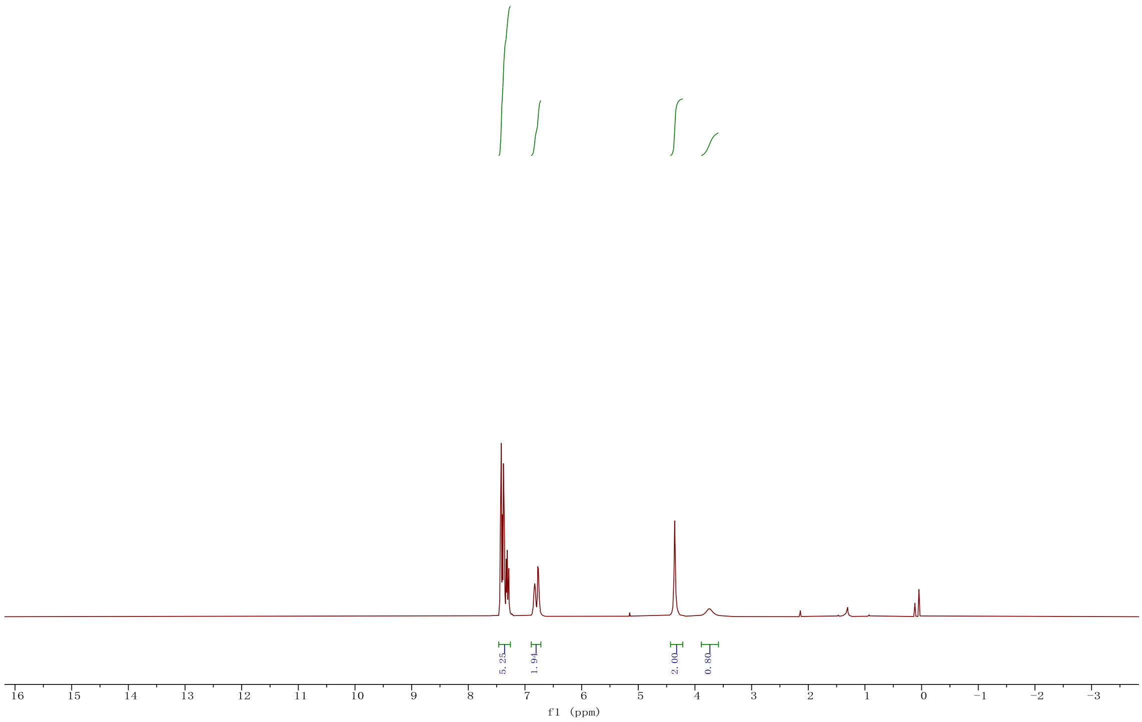

Figure S6-13a. The ^{1}H NMR spectra of **3am** in Chloroform-*d*.

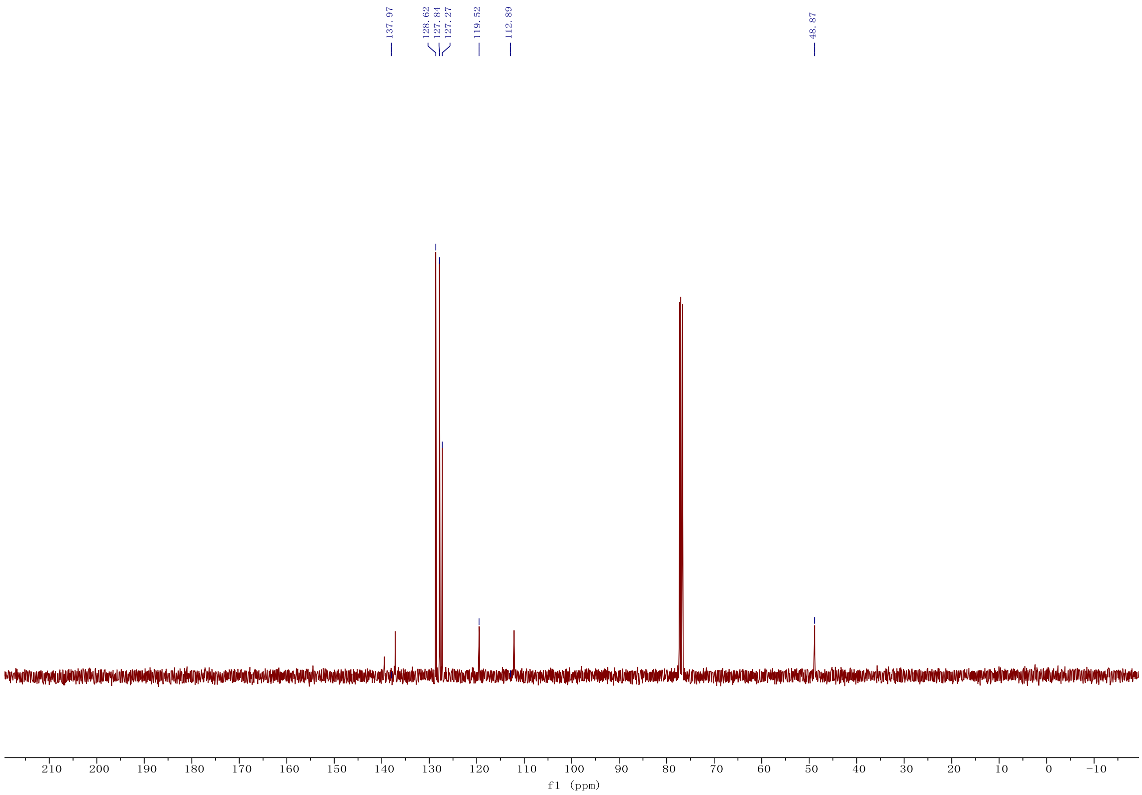

Figure S6-13b. The ^{13}C NMR spectra of **3am** in Chloroform-*d*.

N-benzylnaphthalen-2-amine (**3an**).[6]

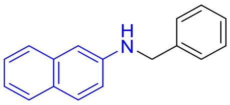

The compound was prepared as described in the general method (oil, 85 % isolated yield, 100 mg). ^{1}H NMR (400 MHz, Chloroform-*d*) δ 7.75 – 7.59 (m, $-C_6H_5$, 3H), 7.46 (d, *J* = 7.0 Hz, $-C_6H_5$, 2H), 7.44 – 7.37 (m, $-C_6H_5$, 3H), 7.37 – 7.31 (m, $-C_6H_5$, 1H), 7.29 – 7.20 (m, $-C_6H_5$, 1H), 6.96 (m, $-C_6H_5$, 1H), 6.89 (d, *J* = 2.4 Hz, $-C_6H_5$, 1H), 4.48 (s, $-CH_2$, 2H), 4.22 (s, -NH, 1H) ppm. ^{13}C NMR (101 MHz, Chloroform-*d*) δ = 48.41, 104.73, 117.87, 122.09, 126.03, 126.35, 127.36, 127.65, 128.72, 128.99, 135.22, 139.22, 145.81 ppm.

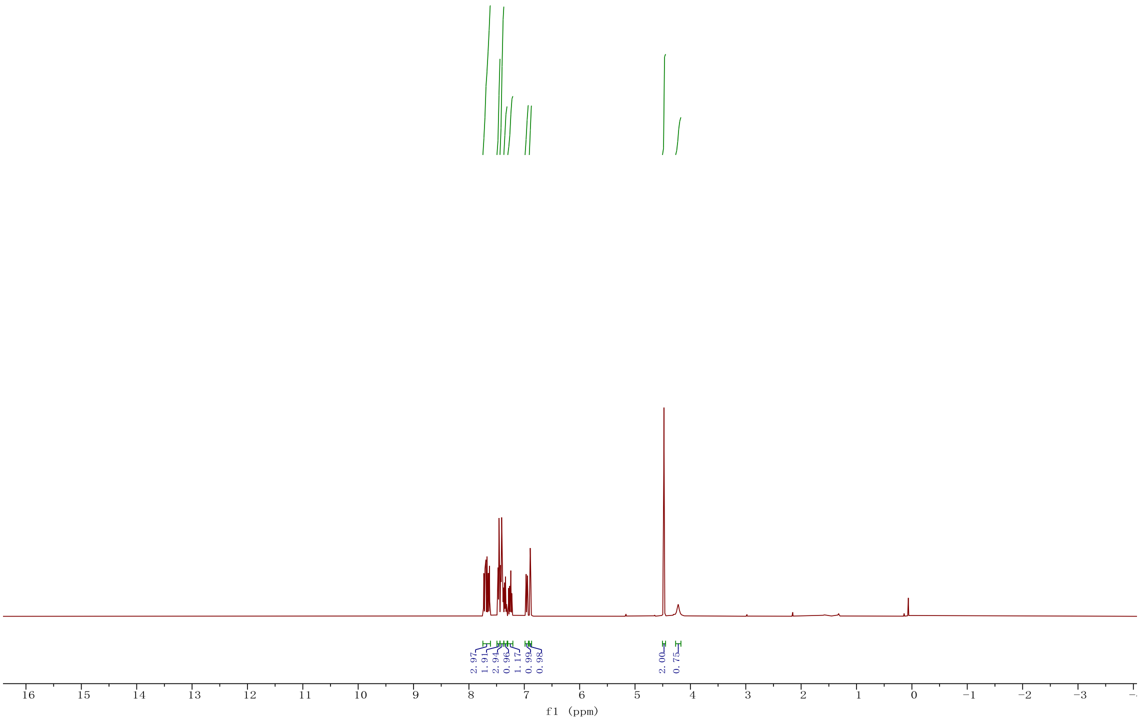

Figure S6-14a. The ^{1}H NMR spectra of **3an** in Chloroform-*d*.

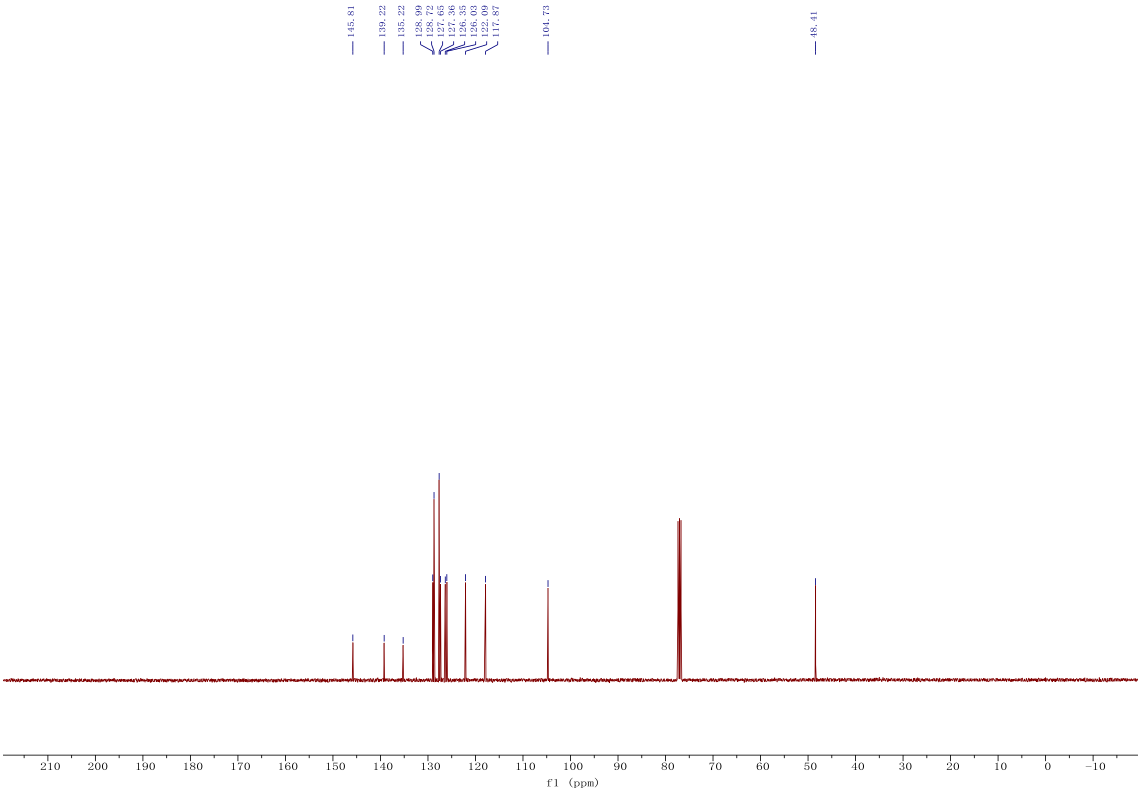

Figure S6-14b. The ^{13}C NMR spectra of **3an** in Chloroform-*d*.

N-benzylnaphthalen-1-amine (**3ao**).[6]

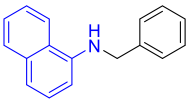

The compound was prepared as described in the general method (oil, 92 % isolated yield, 107 mg). ^{1}H NMR (400 MHz, Chloroform-*d*) δ 7.89 – 7.80 (m, -C_6H_5, 2H), 7.54 – 7.24 (m, -C_6H_5, 9H), 6.67 (d, *J* = 7.4 Hz, -C_6H_5, 1H), 4.72 (s, -NH, 1H), 4.54 (s, -CH_2, 2H) ppm. ^{13}C NMR (101 MHz, Chloroform-*d*) δ = 48.66, 104.77, 117.66, 119.89, 123.42, 124.76, 125.75, 126.61, 127.41, 127.75, 128.71, 128.74, 134.33, 139.13, 143.24 ppm.

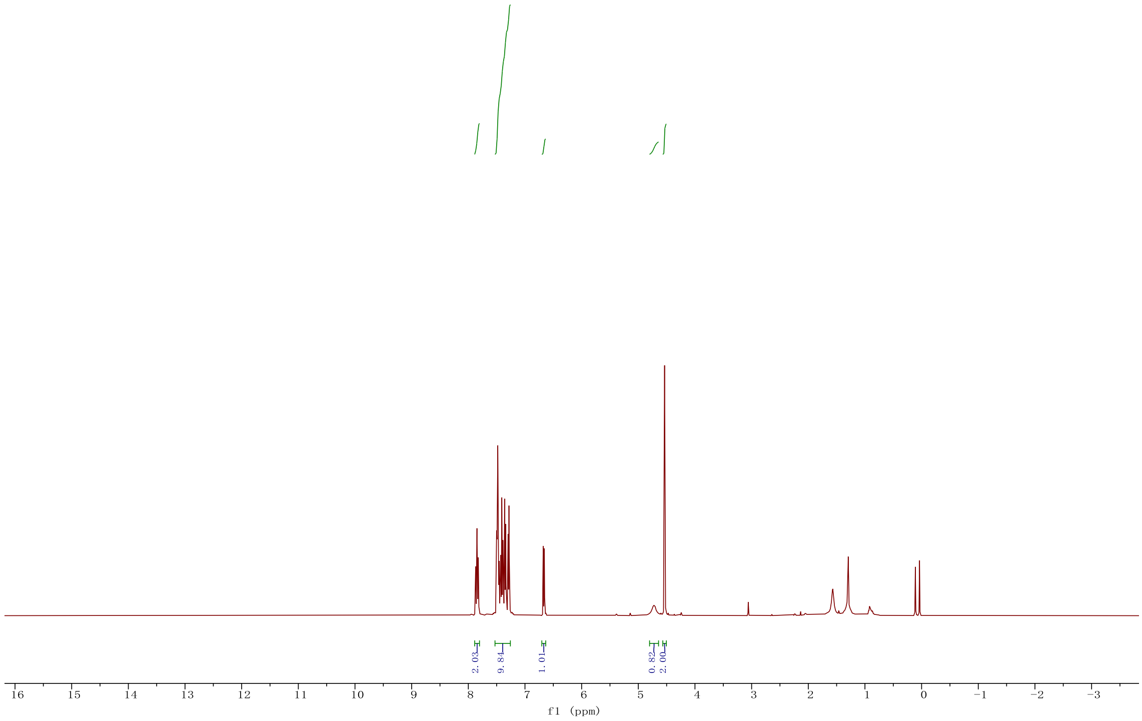

Figure S6-15a. The ^{1}H NMR spectra of **3ao** in Chloroform-*d*.

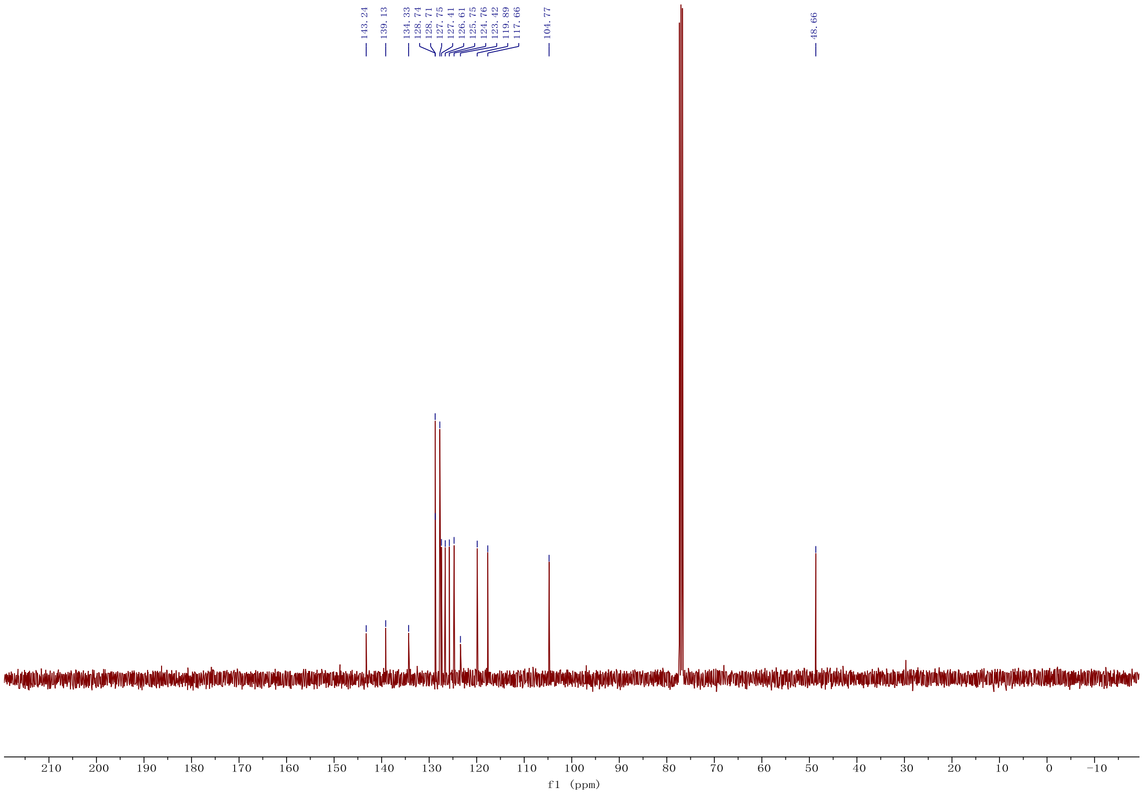

Figure S6-15b. The ^{13}C NMR spectra of **3ao** in Chloroform-*d*.

N-benzylquinolin-8-amine (**3ap**).[6]

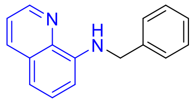

The compound was prepared as described in the general method (colorless oil, 81 % isolated yield, 95 mg). ^{1}H NMR (400 MHz, Chloroform-*d*) δ 8.76 (m, -C_6H_5, 1H), 8.10 (m, -C_6H_5, 1H), 7.52 – 7.44 (m, -C_6H_5, 2H), 7.44 – 7.23 (m, -C_6H_5, 5H), 7.10 (m, -C_6H_5, 1H), 6.67 (m, -C_6H_5, 2H), 4.60 (d, *J* = 5.8 Hz, -CH_2, 2H) ppm. ^{13}C NMR (101 MHz, Chloroform-*d*) δ = 47.77, 105.19, 114.19, 121.44, 127.18, 127.47, 127.82, 128.66, 128.71, 136.04, 138.31, 139.31, 144.67, 146.94 ppm.

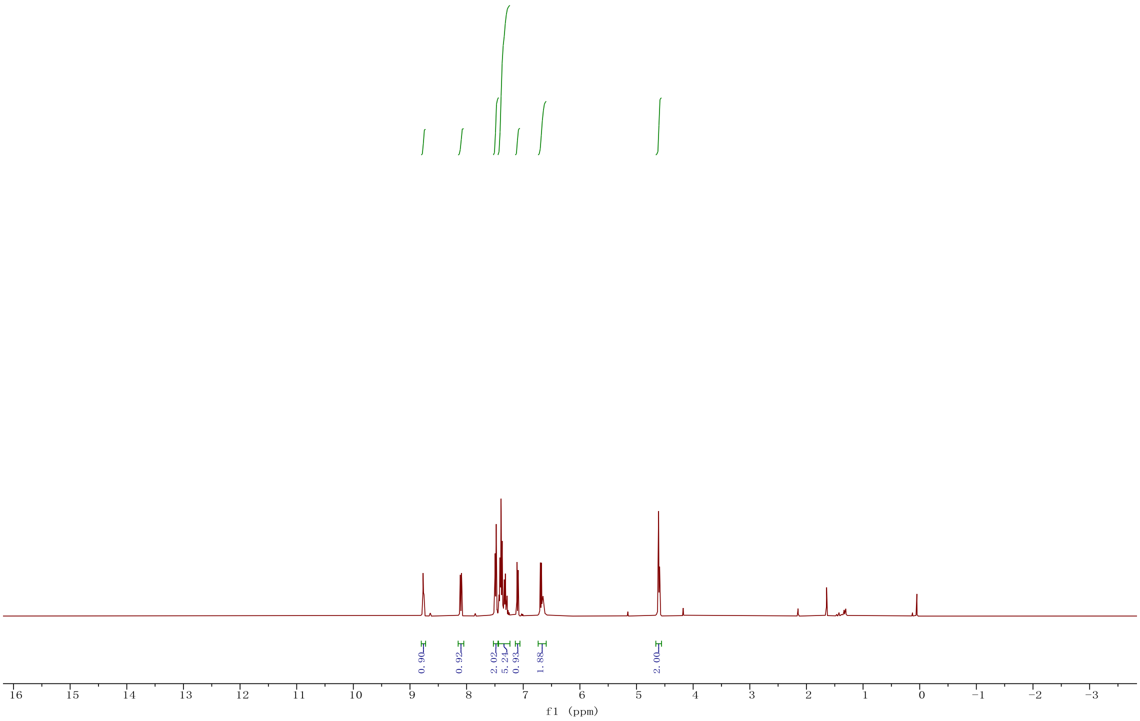

Figure S6-16a. The ^{1}H NMR spectra of **3ap** in Chloroform-*d*.

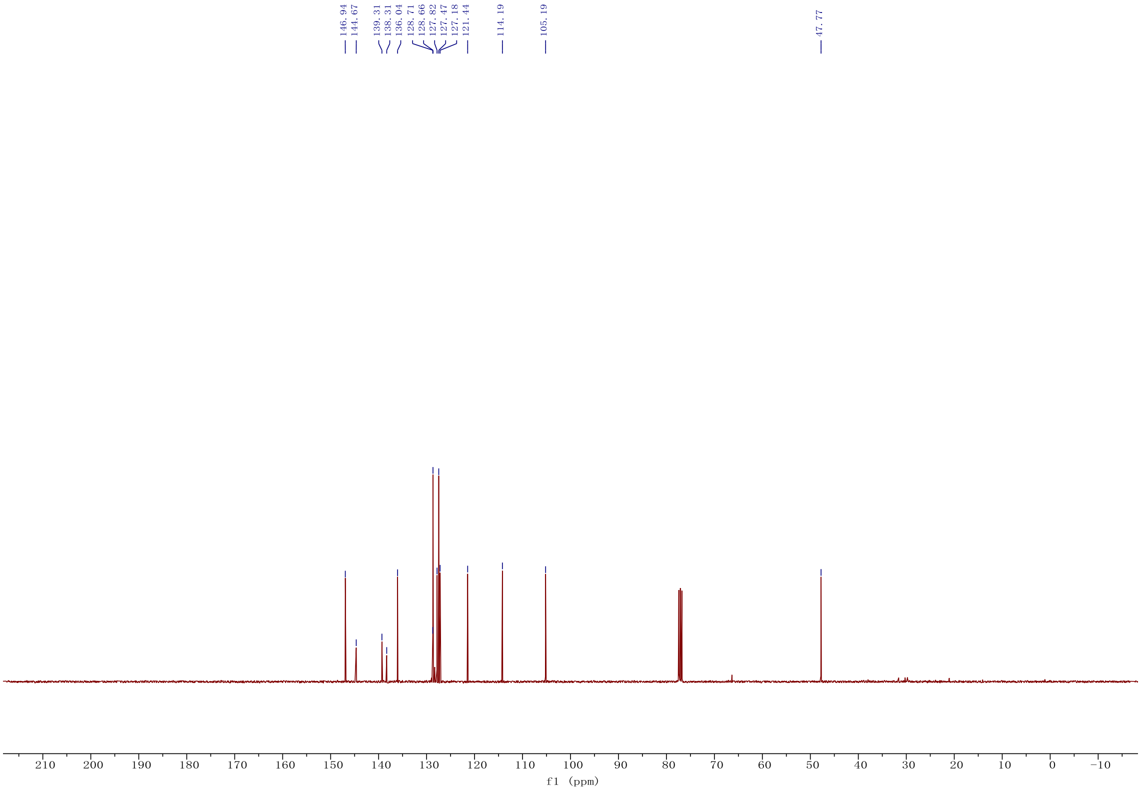

Figure S6-16b. The ^{13}C NMR spectra of **3ap** in Chloroform-*d*.

N-benzylpyridin-3-amine (**3aq**).[6]

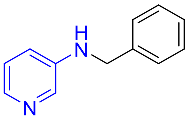

The compound was prepared as described in the general method (solid, 90 % isolated yield, 83 mg). ^{1}H NMR (400 MHz, Chloroform-*d*) δ 8.07 (d, *J* = 2.9 Hz, =CH, 1H), 7.96 (m, =CH, 1H), 7.48 – 7.26 (m, -C_6H_5, 5H), 7.06 (m, =CH, 1H), 6.88 (m, =CH, 1H), 4.59 (s, -NH, 1H), 4.34 (s, -CH_2, 2H) ppm. ^{13}C NMR (101 MHz, Chloroform-*d*) δ = 47.89, 118.64, 123.74, 127.42, 127.51, 128.78, 136.13, 138.54, 138.81, 144.10 ppm.

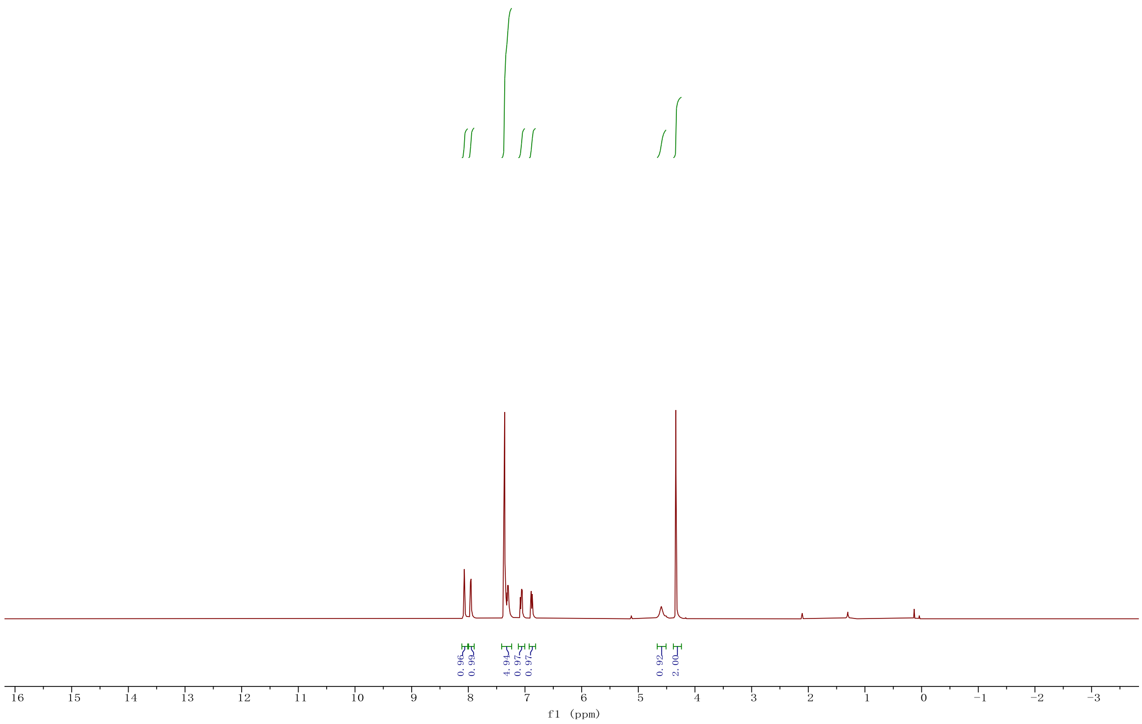

Figure S6-17a. The ^{1}H NMR spectra of **3aq** in Chloroform-*d*.

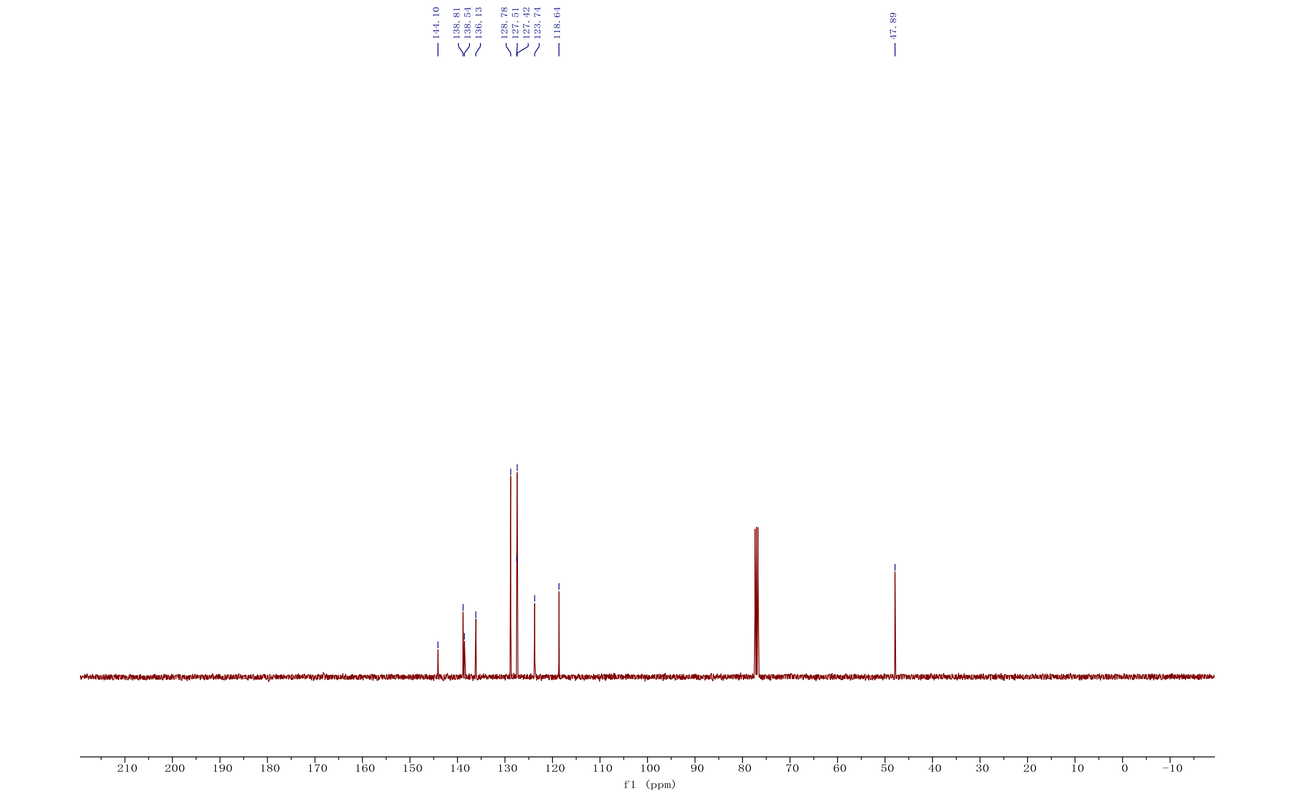

Figure S6-17b. The ^{13}C NMR spectra of **3aq** in Chloroform-*d*.

N-benzyl-9H-fluoren-2-amine (**3ar**).[6]

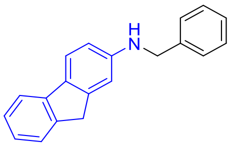

The compound was prepared as described in the general method (colorless oil, 50 % isolated yield, 68 mg). ^{1}H NMR (400 MHz, Chloroform-*d*) δ 7.63 (d, J = 7.6 Hz, -C_6H_5, 1H), 7.58 (d, J = 8.1 Hz, -C_6H_5, 1H), 7.32 (m, -C_6H_5, 6H), 7.23 – 7.11 (m, -C_6H_5, 2H), 6.68 (m, -C_6H_5, 1H), 6.45 (d, J = 2.2 Hz, -C_6H_5, 1H), 4.32 (s, -CH_2, 2H), 4.09 (s, -NH, 1H), 3.24 – 2.99 (m, -CH_2, 2H) ppm. ^{13}C NMR (101 MHz, Chloroform-*d*) δ = 40.37, 48.44, 109.24, 112.31, 118.39, 120.61, 124.49, 124.79, 126.30, 127.01, 128.25, 129.63, 139.37, 140.07, 141.40, 145.92, 147.48, 148.74 ppm.

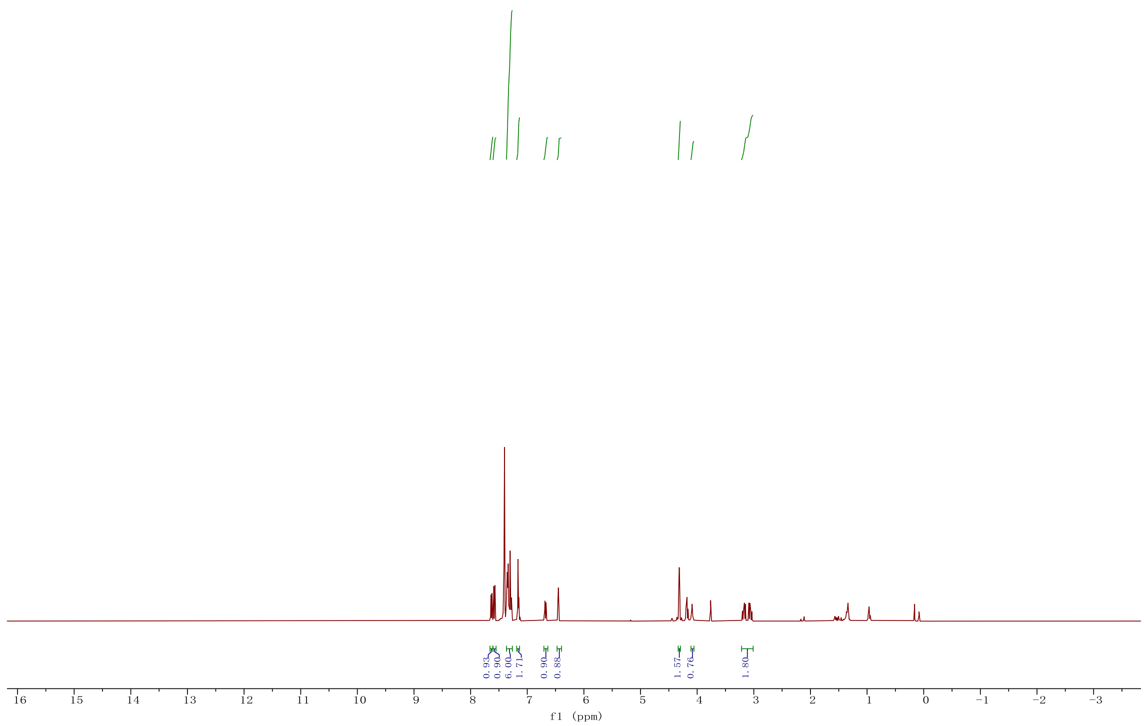

Figure S6-18a. The ^{1}H NMR spectra of **3ar** in Chloroform-*d*.

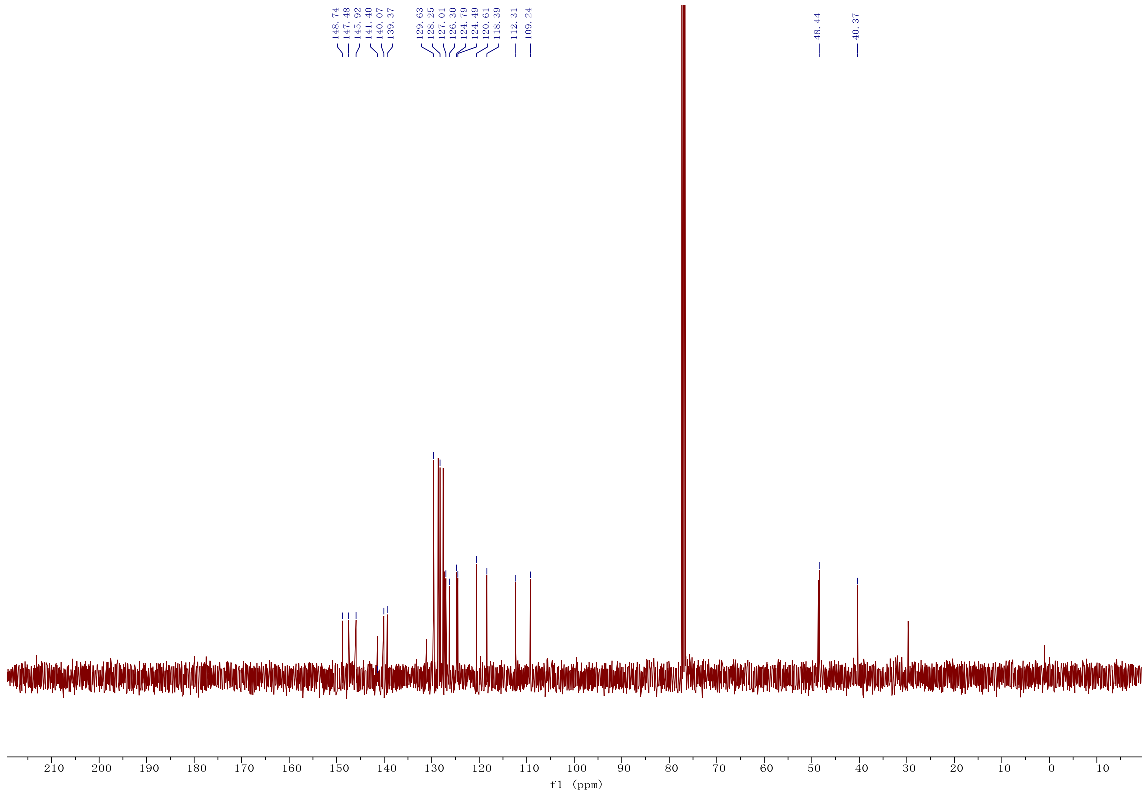

Figure S6-18b. The ^{13}C NMR spectra of **3ar** in Chloroform-*d*.

N-benzylbenzo[d][1,3]dioxol-5-amine (**3as**).[11]

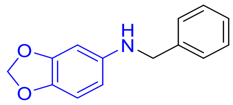

The compound was prepared as described in the general method (oil, 60 % isolated yield, 68 mg). ^{1}H NMR (400 MHz, Chloroform-*d*) δ 7.39 (s, -C_6H_5, 5H), 6.69 (d, *J* = 8.2 Hz, -C_6H_5, 1H), 6.30 (d, *J* = 2.3 Hz, -C_6H_5, 1H), 6.11 (m, -C_6H_5, 1H), 5.88 (s, -CH_2, 2H), 4.29 (s, -CH_2, 2H), 3.87 (s, -NH, 1H) ppm. ^{13}C NMR (101 MHz, Chloroform-*d*) δ = 49.29, 96.04, 100.58, 104.46, 108.65, 127.26, 127.54, 128.65, 139.46, 139.74, 143.99, 148.37 ppm.

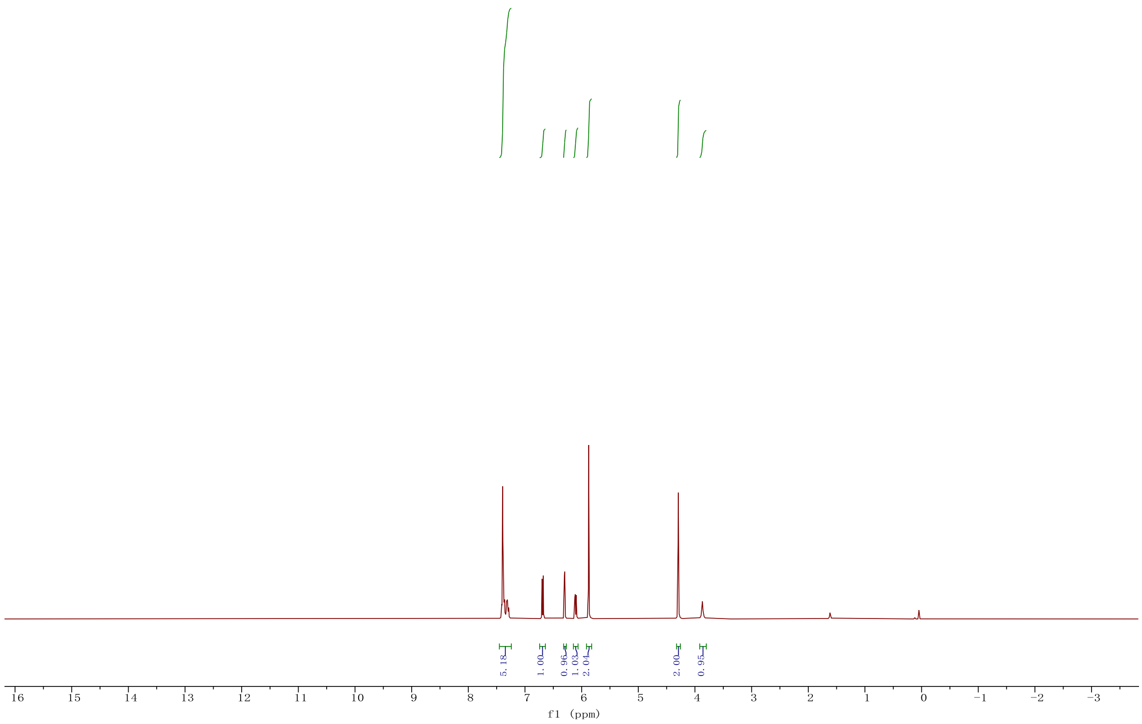

Figure S6-19a. The ^{1}H NMR spectra of **3as** in Chloroform-*d*.

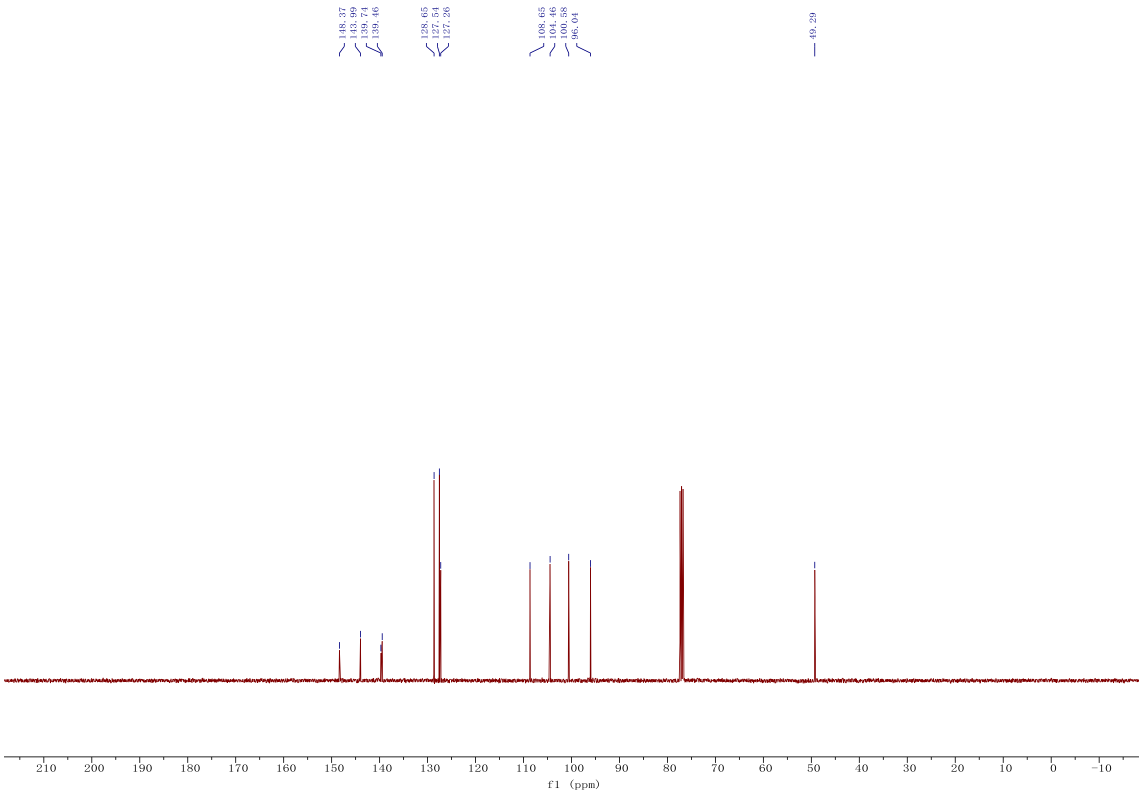

Figure S6-19b. The ^{13}C NMR spectra of **3as** in Chloroform-*d*.

N-benzylpyridin-2-amine (**3at**).[6]

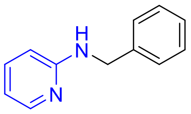

The compound was prepared as described in the general method (solid, 79% isolated yield, 73 mg). ^{1}H NMR (400 MHz, Chloroform-*d*) δ 8.17 – 8.12 (m, -C_6H_5, 1H), 7.47 – 7.27 (m, -C_6H_5, 6H), 6.62 (t, J = 6.2 Hz, -C_6H_5, 1H), 6.40 (d, J = 8.4 Hz, -C_6H_5, 1H), 4.91 (s, -NH, 1H), 4.53 (s, -CH_2, 2H) ppm. ^{13}C NMR (101 MHz, Chloroform-*d*) δ = 43.89, 107.78, 114.50, 127.29, 127.42, 128.68, 137.57, 140.44, 147.67, 160.23 ppm.

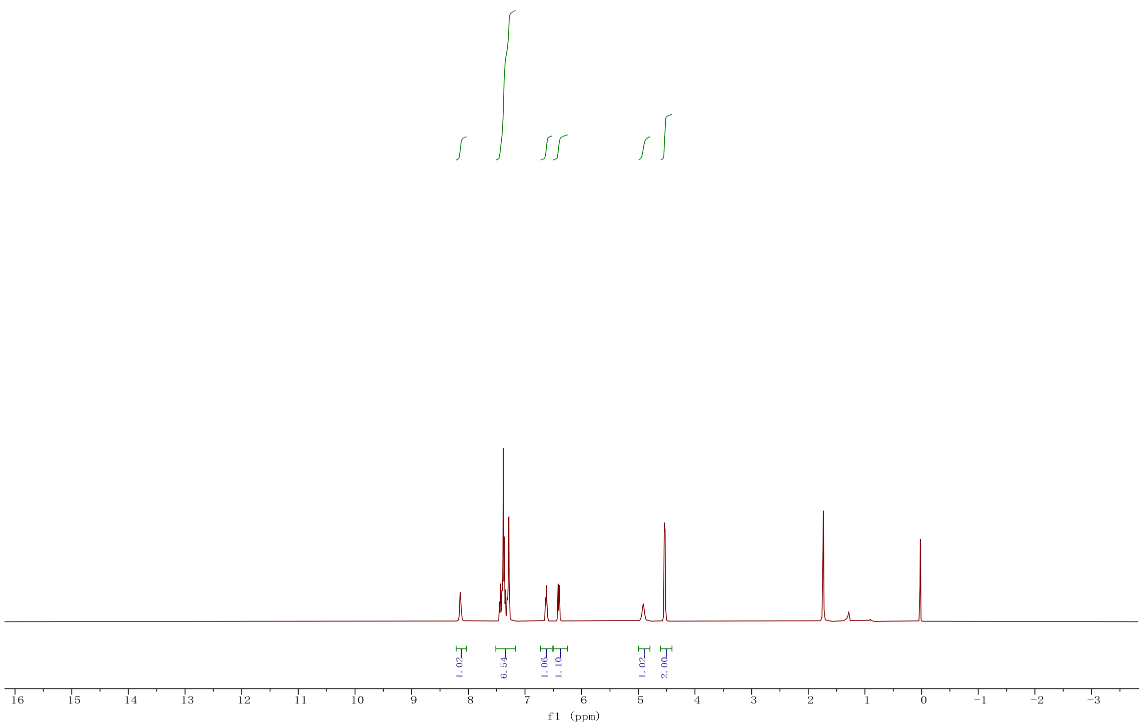

Figure S6-20a. The ^{1}H NMR spectra of **3at** in Chloroform-*d*.

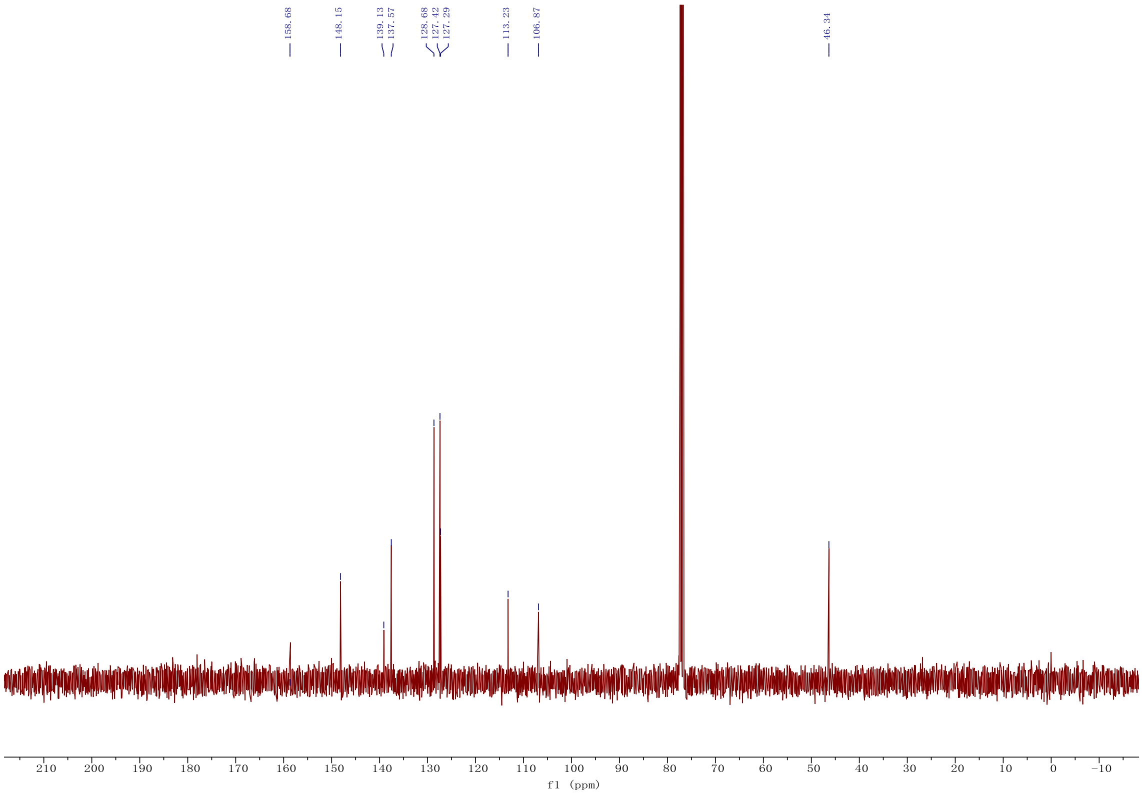

Figure S6-20b. The ^{13}C NMR spectra of **3at** in Chloroform-*d*.

N-(4-methoxybenzyl)aniline (**3ba**).[6]

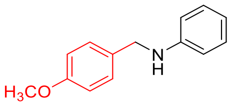

The compound was prepared as described in the general method (oil, 97% isolated yield, 103 mg). ^{1}H NMR (400 MHz, Chloroform-*d*) δ 7.43 – 7.09 (m, $-C_6H_5$, 4H), 6.91 (d, *J* = 8.2 Hz, $-C_6H_5$, 2H), 6.74 (t, *J* = 7.3 Hz, $-C_6H_5$, 1H), 6.67 (d, *J* = 8.0 Hz, $-C_6H_5$, 2H), 4.28 (s, $-CH_2$, 2H), 4.11 (s, -NH, 1H), 3.83 (s, $-OCH_3$, 3H) ppm. ^{13}C NMR (101 MHz, Chloroform-*d*) δ = 47.85, 54.33, 112.89, 114.60, 117.19, 128.86, 129.28, 131.33, 146.30, 160.84 ppm.

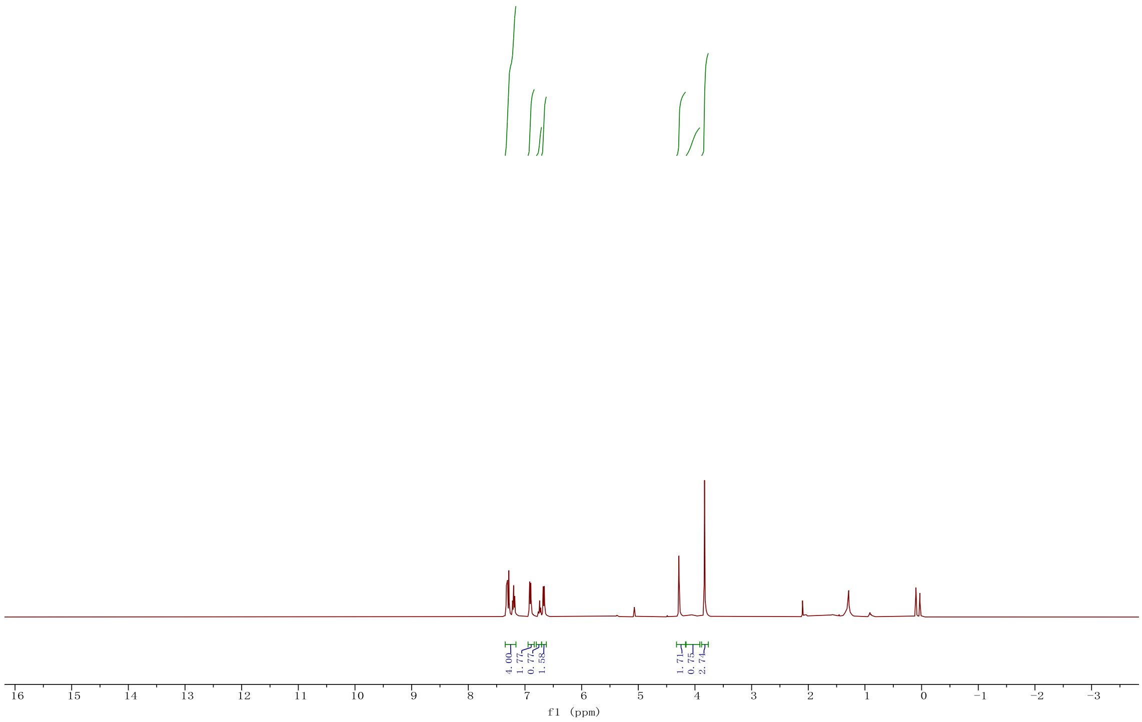

Figure S6-21a. The ^{1}H NMR spectra of **3ba** in Chloroform-*d*.

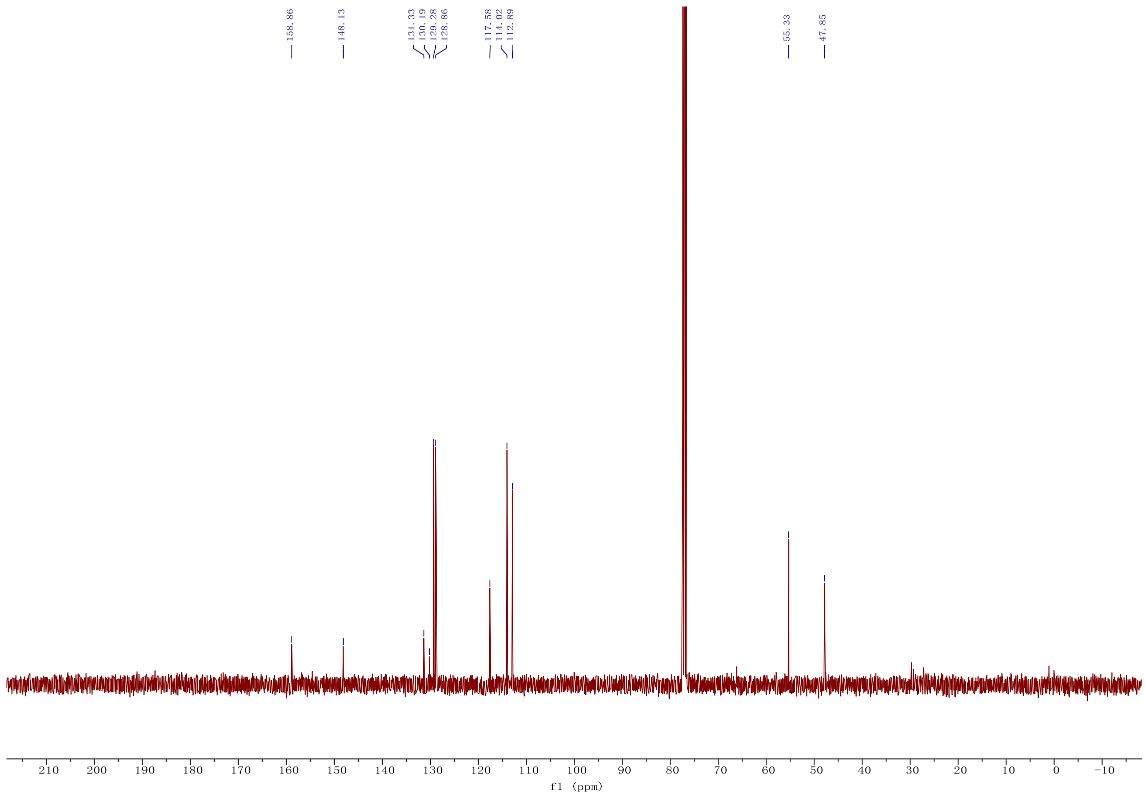

Figure S6-21b. The ^{13}C NMR spectra of **3ba** in Chloroform-*d*.

N-(3-methoxybenzyl)aniline (**3ca**).[12]

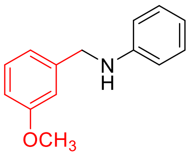

The compound was prepared as described in the general method (oil, 86% isolated yield, 92 mg). ^{1}H NMR (400 MHz, Chloroform-*d*) δ 7.30 – 7.15 (m, -C_6H_5, 3H), 7.03 – 6.94 (m, -C_6H_5, 2H), 6.84 (m, -C_6H_5, 1H), 6.74 (m, -C_6H_5, 1H), 6.70 – 6.63 (m, -C_6H_5, 2H), 4.34 (s, -CH_2, 2H), 4.09 (s, -NH, 1H), 3.83 (s, -OCH_3, 3H) ppm. ^{13}C NMR (101 MHz, Chloroform-*d*) δ = 48.38, 55.22, 112.69, 112.91, 113.06, 117.65, 119.76, 129.26, 129.65, 141.16, 148.12, 159.95 ppm.

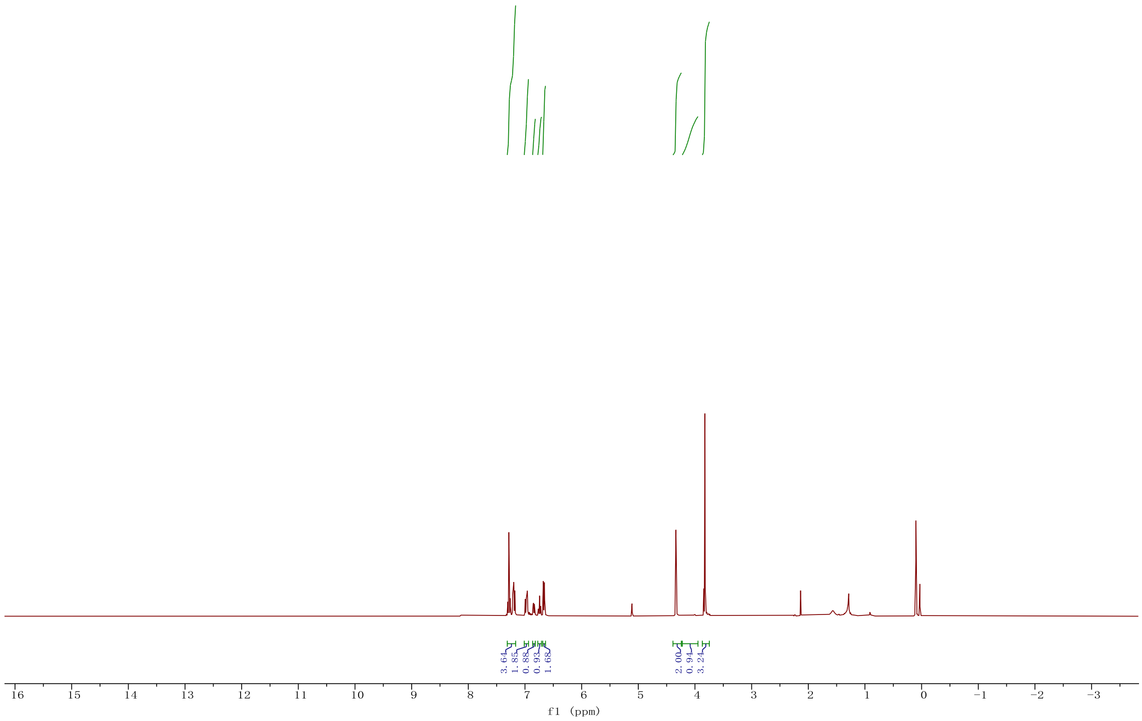

Figure S6-22a. The ^{1}H NMR spectra of **3ca** in Chloroform-*d*.

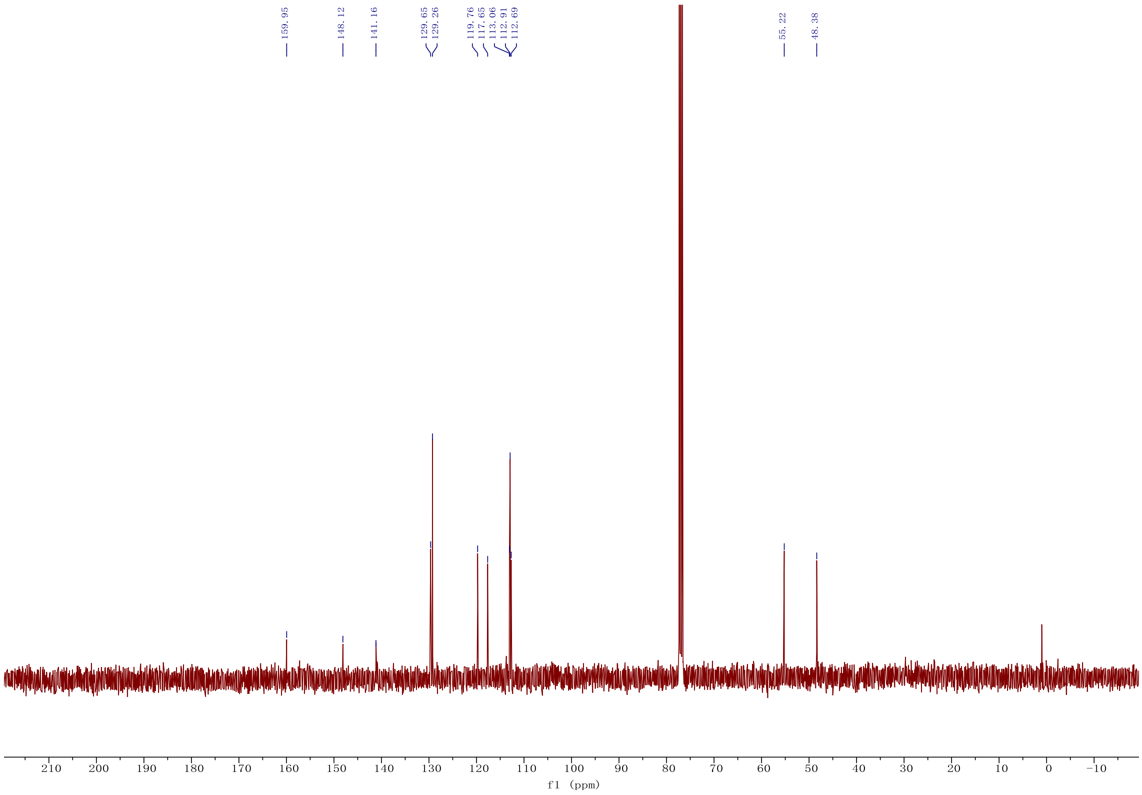

Figure S6-22b. The ^{13}C NMR spectra of **3ca** in Chloroform-*d*.

N-(2-methoxybenzyl)aniline (**3da**).[13]

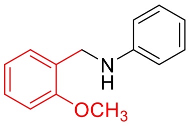

The compound was prepared as described in the general method (oil, 86% isolated yield, 92 mg). ^{1}H NMR (400 MHz, Chloroform-*d*) δ 7.45 – 7.17 (m, -C_6H_5, 4H), 7.01 – 6.91 (m, -C_6H_5, 2H), 6.79 – 6.68 (m, -C_6H_5, 3H), 4.40 (s, -CH_2, 2H), 4.16 (s, -NH, 1H), 3.91 (s, -OCH_3, 3H) ppm. ^{13}C NMR (101 MHz, Chloroform-*d*) δ = 43.50, 55.33, 110.31, 113.11, 117.37, 120.56, 127.41, 128.31, 128.94, 129.19, 148.47, 157.44 ppm.

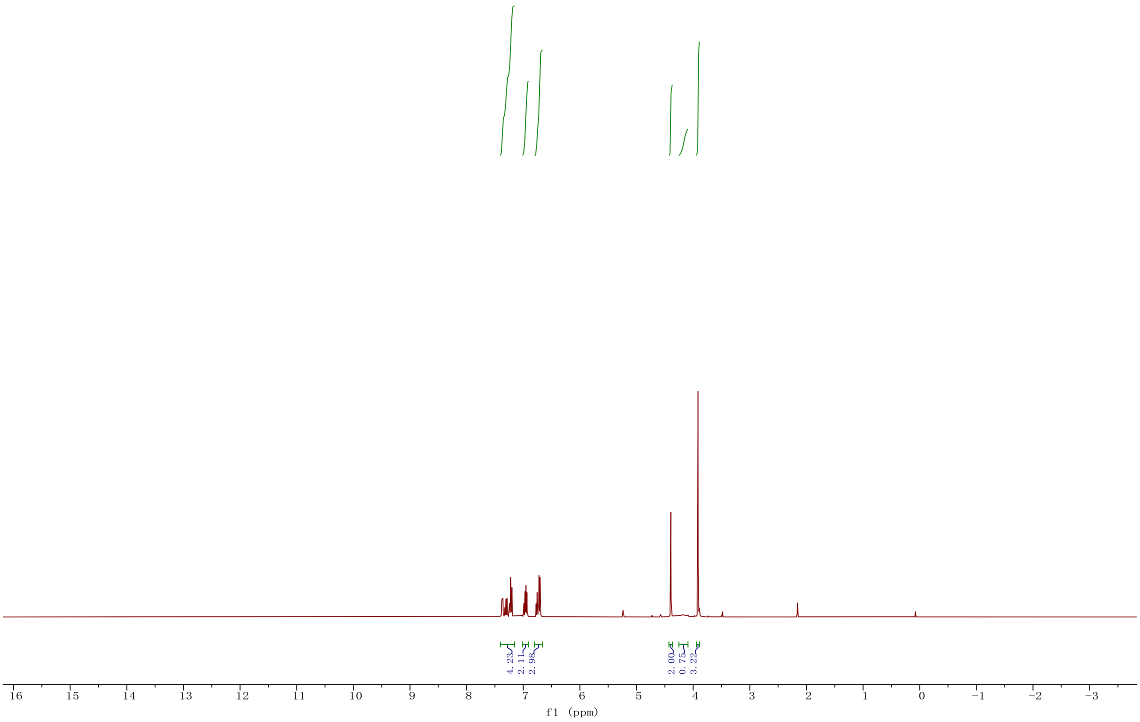

Figure S6-23a. The ^{1}H NMR spectra of **3da** in Chloroform-*d*.

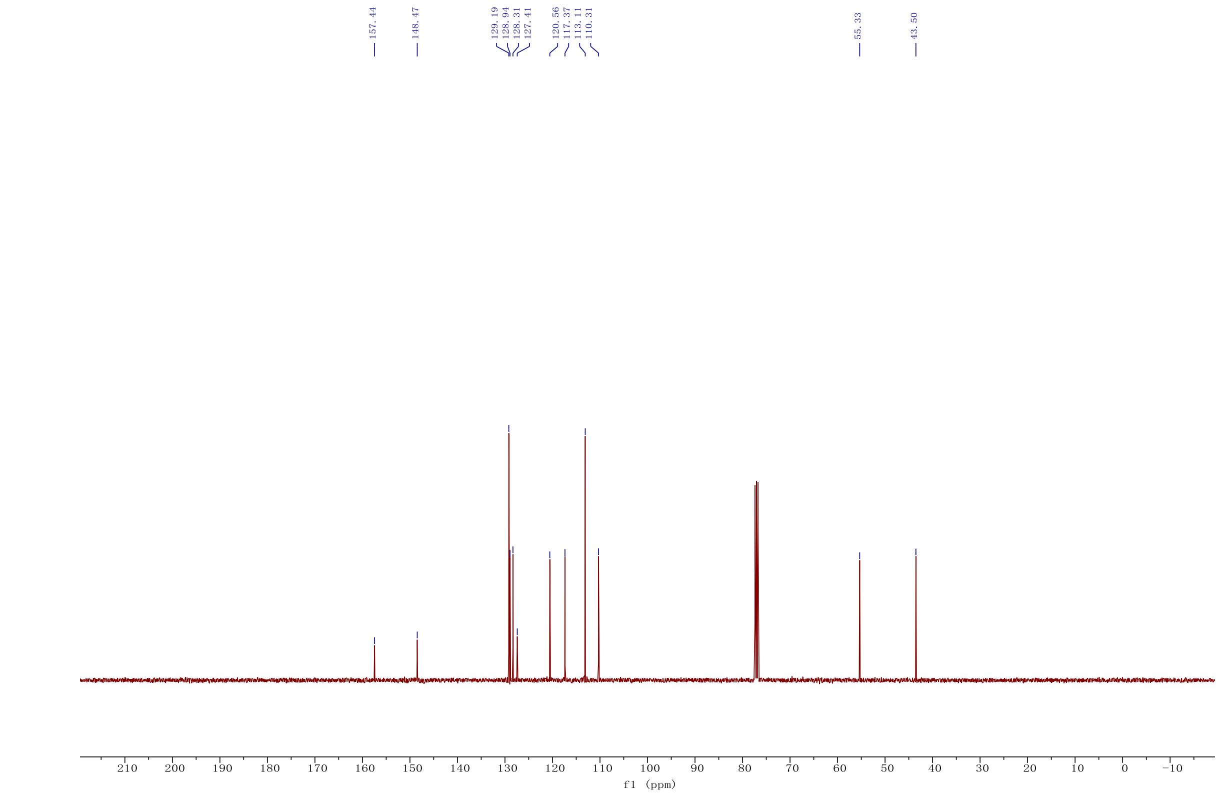

Figure S6-23b. The ^{13}C NMR spectra of **3da** in Chloroform-*d*.

N-(4-methylbenzyl)aniline (**3ea**).[6]

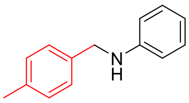

The compound was prepared as described in the general method (oil, 89% isolated yield, 88 mg). ^{1}H NMR (400 MHz, Chloroform-*d*) δ 7.45 – 7.04 (m, -C_6H_5, 6H), 6.85 – 6.55 (m, -C_6H_5, 3H), 4.31 (s, -CH_2, 2H), 4.12 (s, -NH, 1H), 2.37 (s, -CH_3, 3H) ppm. ^{13}C NMR (101 MHz, Chloroform-*d*) δ = 21.14, 48.12, 112.88, 117.56, 127.56, 129.28, 129.33, 136.29, 136.92, 148.15 ppm.

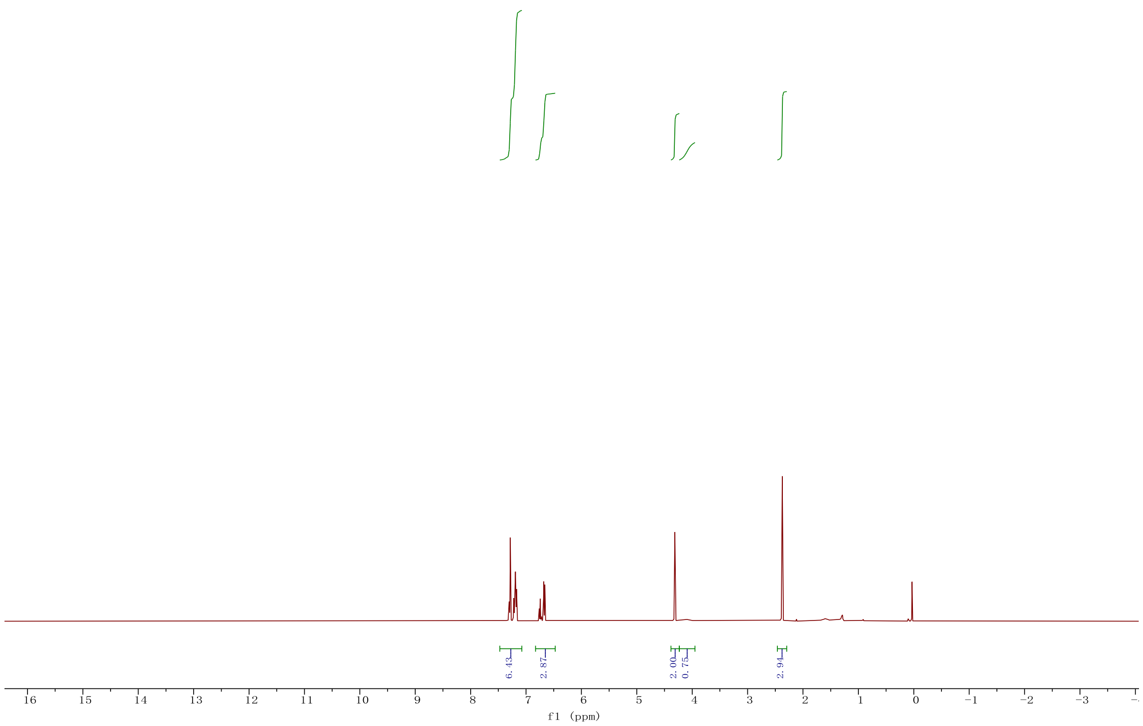

Figure S6-24a. The ^{1}H NMR spectra of **3ea** in Chloroform-*d*.

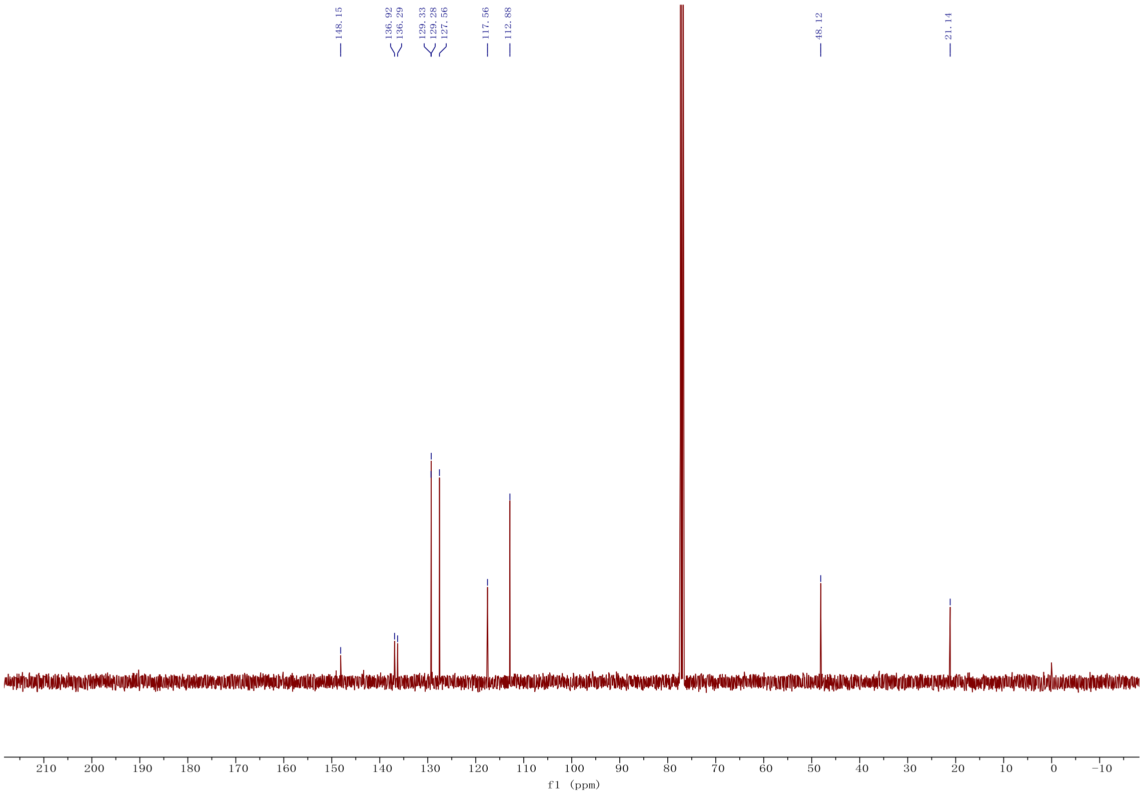

Figure S6-24b. The ^{13}C NMR spectra of **3ea** in Chloroform-*d*.

N-(3-methylbenzyl)aniline (**3fa**).[6]

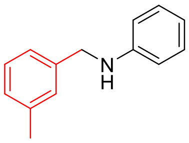

The compound was prepared as described in the general method (oil, 82% isolated yield, 81 mg). ^{1}H NMR (400 MHz, Chloroform-*d*) δ 7.27 – 7.04 (m, -C_6H_5, 6H), 6.78 – 6.64 (m, -C_6H_5, 3H), 4.31 (s, -CH_2, 2H), 4.11 (s, -NH, 1H), 2.38 (s, -CH_3, 3H) ppm. ^{13}C NMR (101 MHz, Chloroform-*d*) δ = 22.60, 47.79, 113.26, 117.93, 124.61, 128.02, 128.33, 128.56, 129.27, 136.79, 138.01, 148.66 ppm.

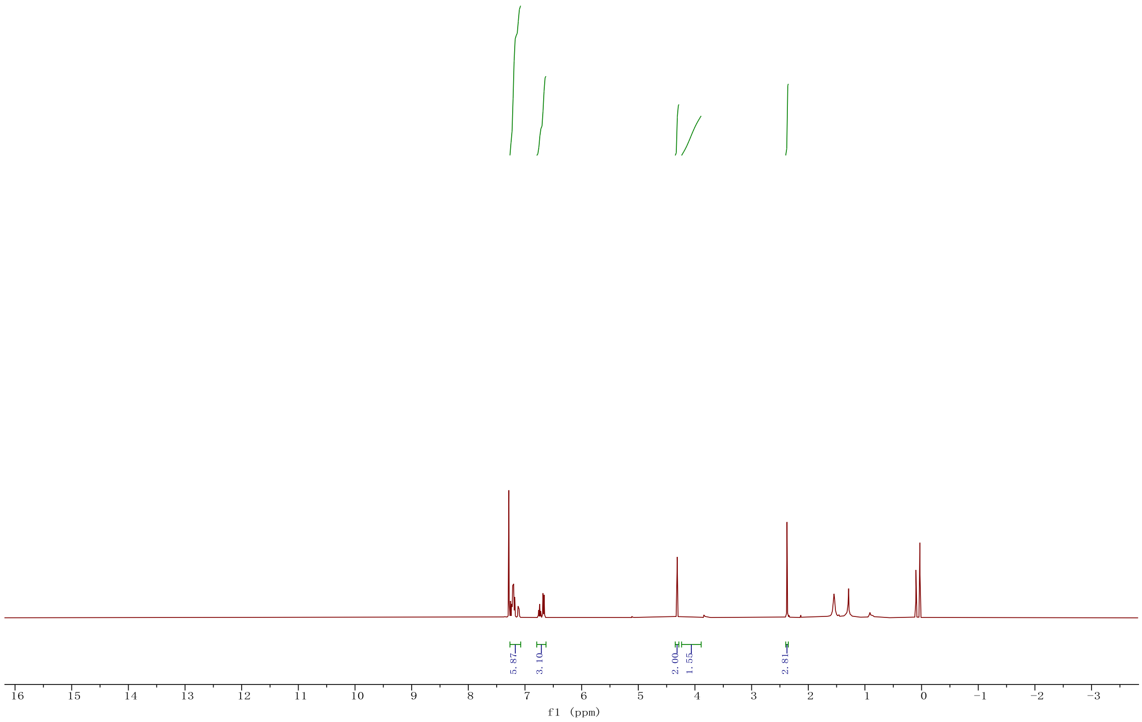

Figure S6-25a. The ^{1}H NMR spectra of **3fa** in Chloroform-*d*.

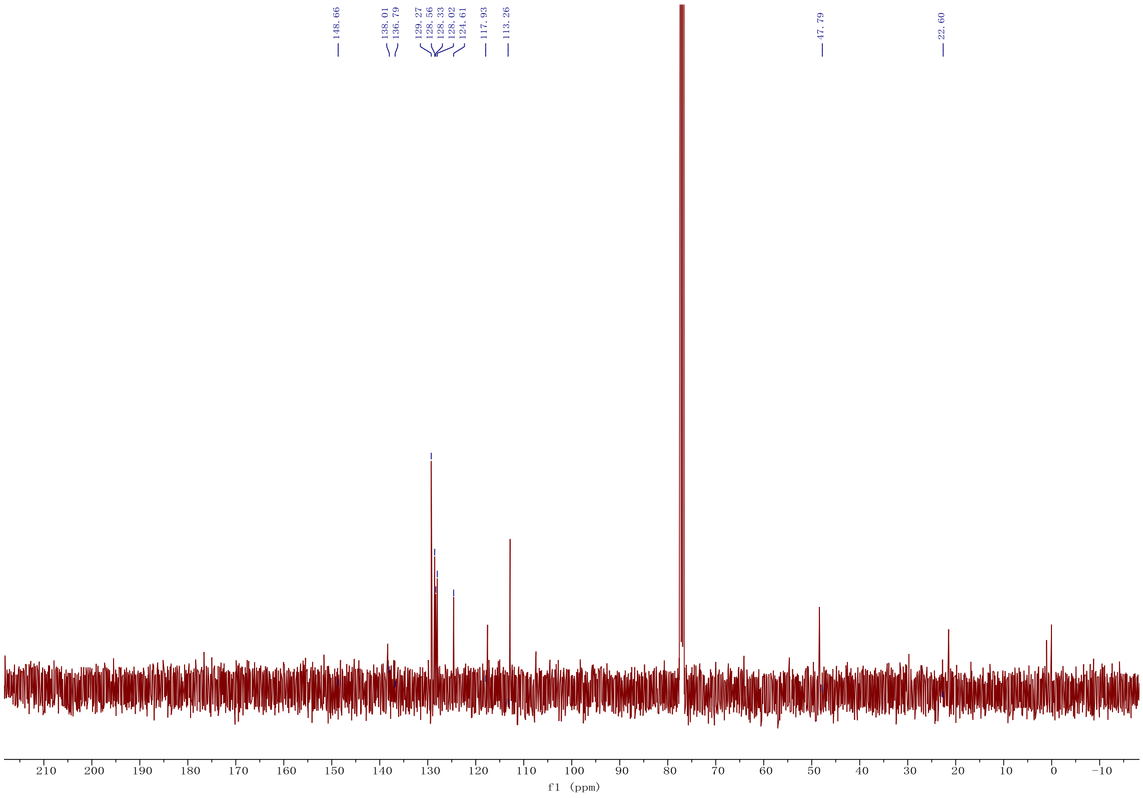

Figure S6-25b. The ^{13}C NMR spectra of **3fa** in Chloroform-*d*.

N-(2-methylbenzyl)aniline (**3ga**).[6]

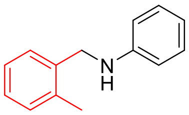

The compound was prepared as described in the general method (oil, 89 % isolated yield, 88 mg). ^{1}H NMR (400 MHz, Chloroform-*d*) δ 7.38 – 7.33 (m, -C_6H_5, 1H), 7.25 – 7.17 (m, -C_6H_5, 5H), 6.79 – 6.61 (m, -C_6H_5, 3H), 4.30 (s, -CH_2, 2H), 3.89 (s, -NH, 1H), 2.40 (s, -CH_3, 3H) ppm. ^{13}C NMR (101 MHz, Chloroform-*d*) δ = 20.17, 48.00, 113.57, 119.23, 126.18, 126.98, 127.86, 129.30, 131.71, 136.56, 137.42, 147.34 ppm.

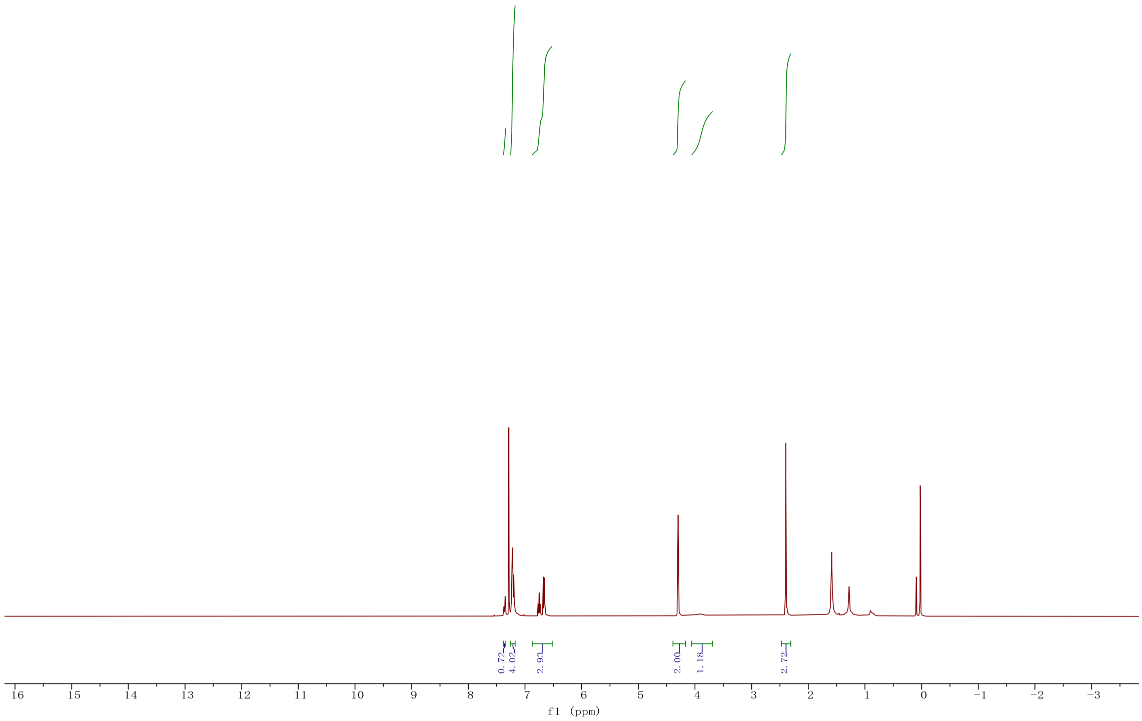

Figure S6-26a. The ^{1}H NMR spectra of **3ga** in Chloroform-*d*.

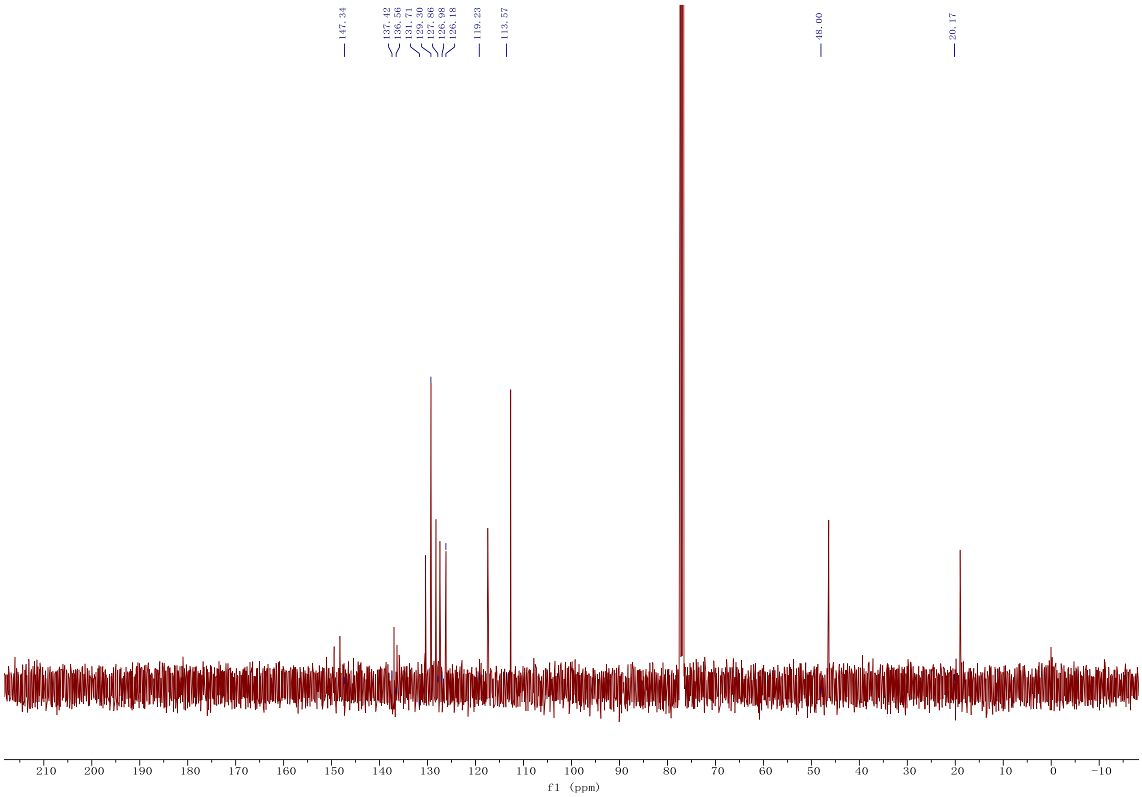

Figure S6-26b. The ^{13}C NMR spectra of **3ga** in Chloroform-*d*.

N-(3-(trifluoromethyl)benzyl)aniline (**3ha**).[8]

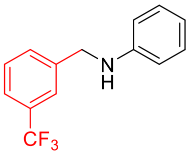

The compound was prepared as described in the general method (oil, 80 % isolated yield, 101 mg). ^{1}H NMR (400 MHz, Chloroform-*d*) δ 7.69 (s, -C_6H_5, 1H), 7.66 – 7.59 (m, -C_6H_5, 2H), 7.59 – 7.43 (m, -C_6H_5, 1H), 7.31 – 7.19 (m, -C_6H_5, 2H), 6.80 (m, -C_6H_5, 1H), 6.67 (d, J = 8.0 Hz, -C_6H_5, 2H), 4.44 (s, -CH_2, 2H), 4.13 (s, -NH, 1H) ppm. ^{13}C NMR (101 MHz, Chloroform-*d*) δ = 47.96, 112.98, 118.05, 124.10, 129.11, 129.37, 130.66, 140.70, 147.78 ppm.

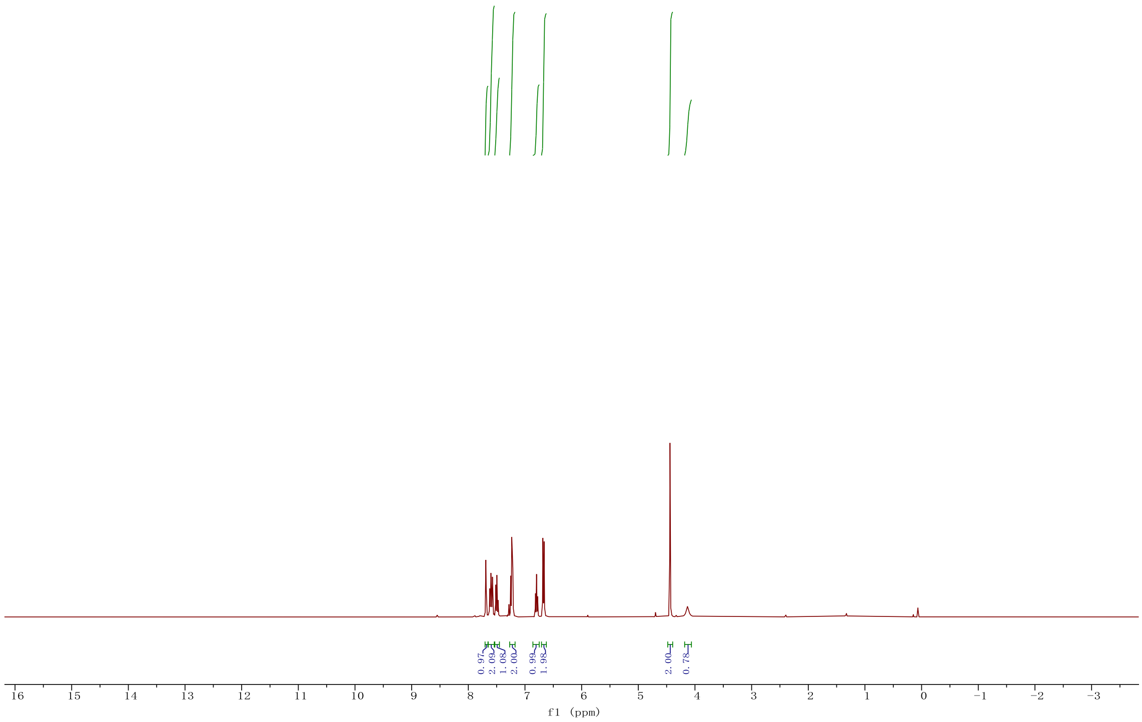

Figure S6-27a. The ^{1}H NMR spectra of **3ha** in Chloroform-*d*.

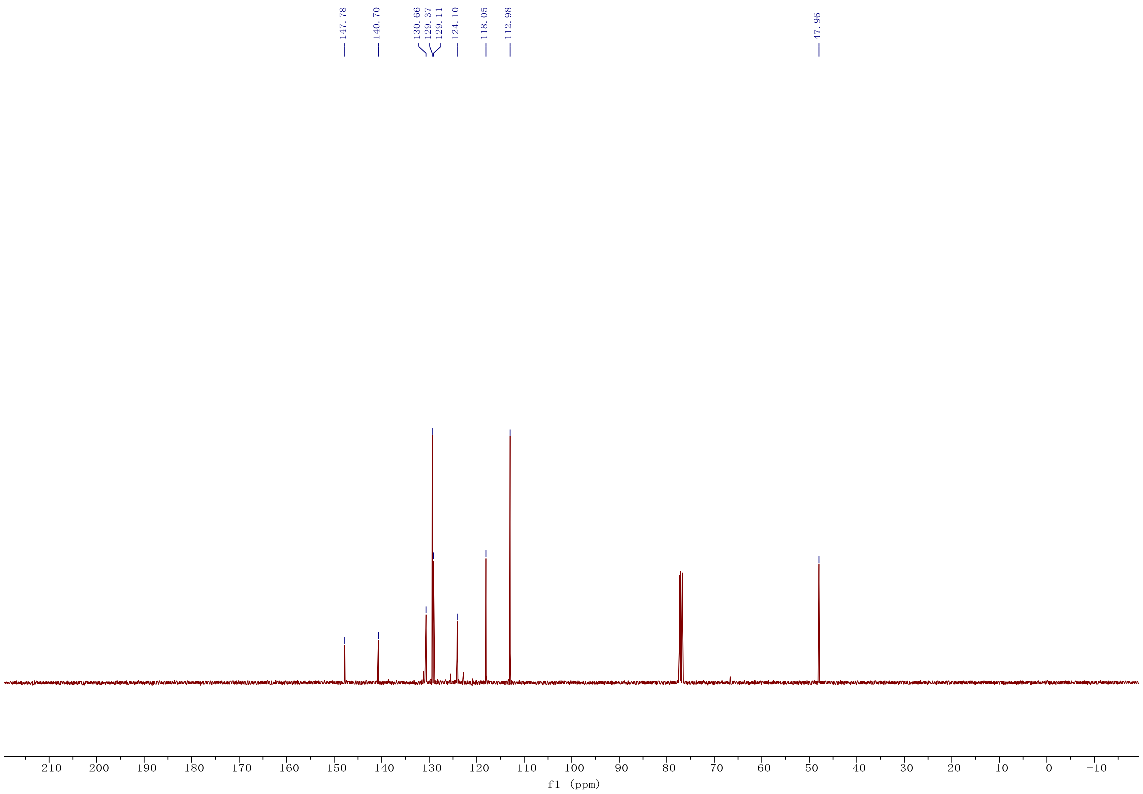

Figure S6-27b. The ^{13}C NMR spectra of **3ha** in Chloroform-*d*.

N-(cyclohexylmethyl)aniline (**3ia**).[13]

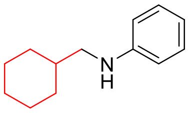

The compound was prepared as described in the general method (oil, 65 % isolated yield, 61 mg). ^{1}H NMR (400 MHz, Chloroform-*d*) δ 7.20 (t, *J* = 7.7 Hz, -C_6H_5, 2H), 6.71 (t, *J* = 7.3 Hz, -C_6H_5, 1H), 6.63 (d, *J* = 8.0 Hz, -C_6H_5, 2H), 3.73 (s, -NH, 1H), 2.98 (d, -CH_2, 2H), 2.07 – 0.80 (m, -CH_2, 11H) ppm. ^{13}C NMR (101 MHz, Chloroform-*d*) δ = 26.01, 26.61, 31.34, 37.58, 50.62, 112.63, 116.89, 129.24, 148.66 ppm.

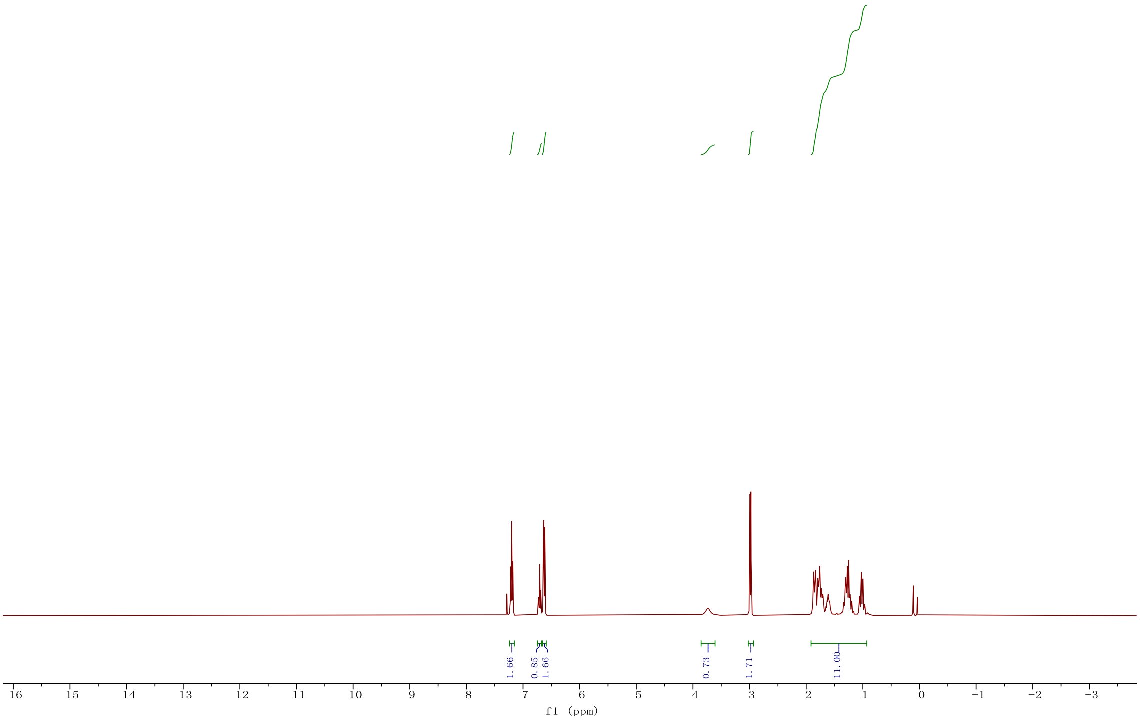

Figure S6-28a. The ^{1}H NMR spectra of **3ia** in Chloroform-*d*.

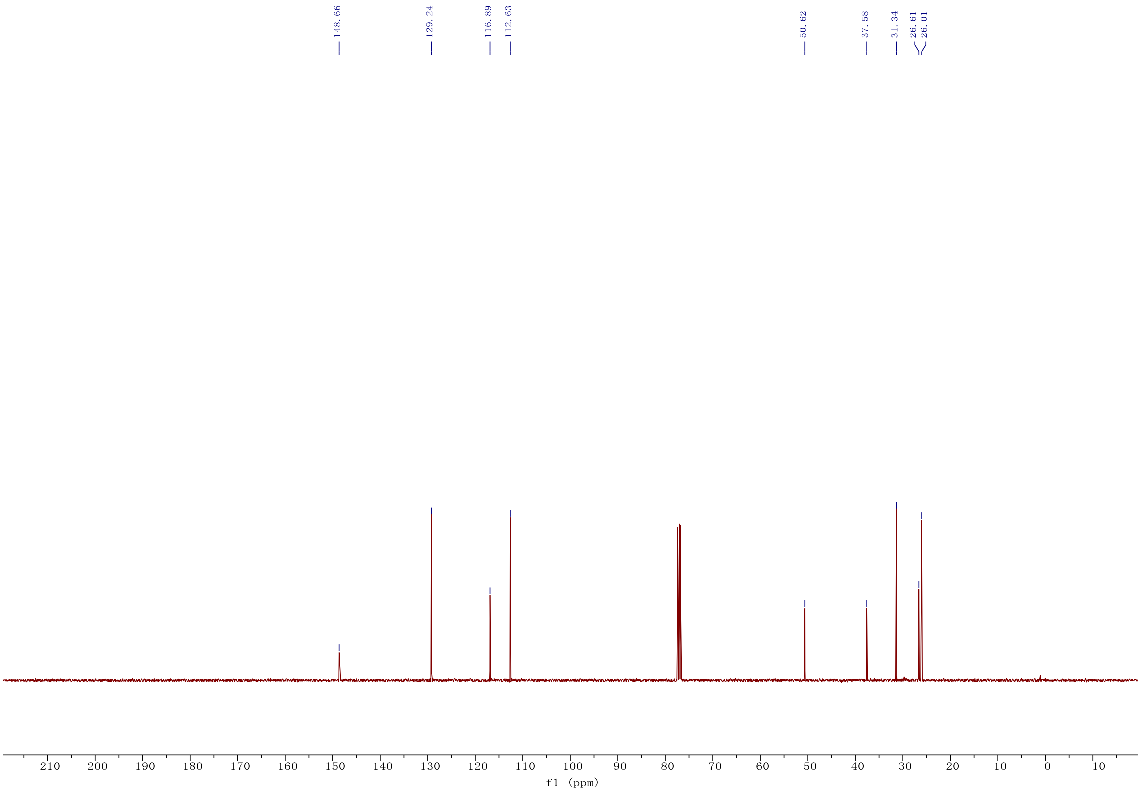

Figure S6-28b. The ^{13}C NMR spectra of **3ia** in Chloroform-*d*.

N-(4-(methylthio)benzyl)aniline (**3ja**).[6]

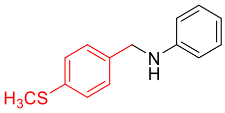

The compound was prepared as described in the general method (oil, 94 % isolated yield, 108 mg). ^{1}H NMR (400 MHz, Chloroform-d) δ 7.32 – 7.22 (m, -C_6H_5, 4H), 7.20 – 7.12 (m, -C_6H_5, 2H), 6.71 (t, J = 7.3 Hz, -C_6H_5, 1H), 6.65 – 6.58 (m, -C_6H_5, 2H), 4.28 (s, -CH_2, 2H), 3.99 (s, -NH, 1H), 2.47 (s, -SCH_3, 3H) ppm. ^{13}C NMR (101 MHz, Chloroform-*d*) δ 16.11, 47.92, 112.93, 117.69, 127.08, 128.08, 129.33, 136.48, 137.23, 148.10 ppm.

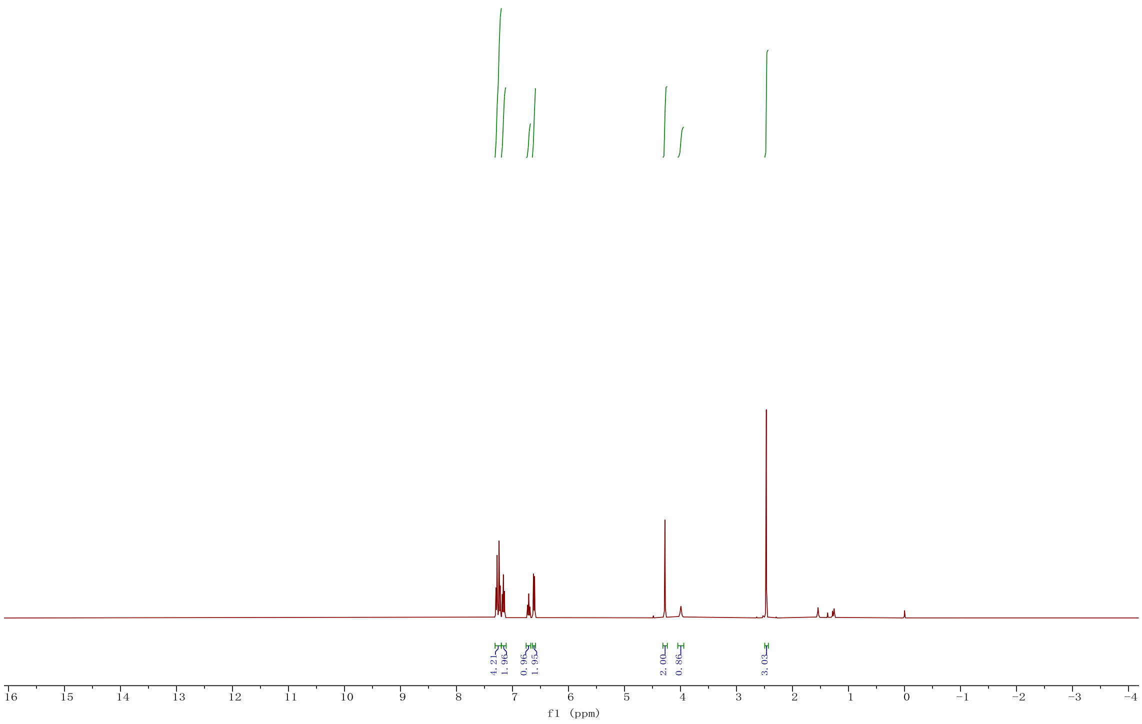

Figure S6-29a. The ^{1}H NMR spectra of **3ja** in Chloroform-*d*.

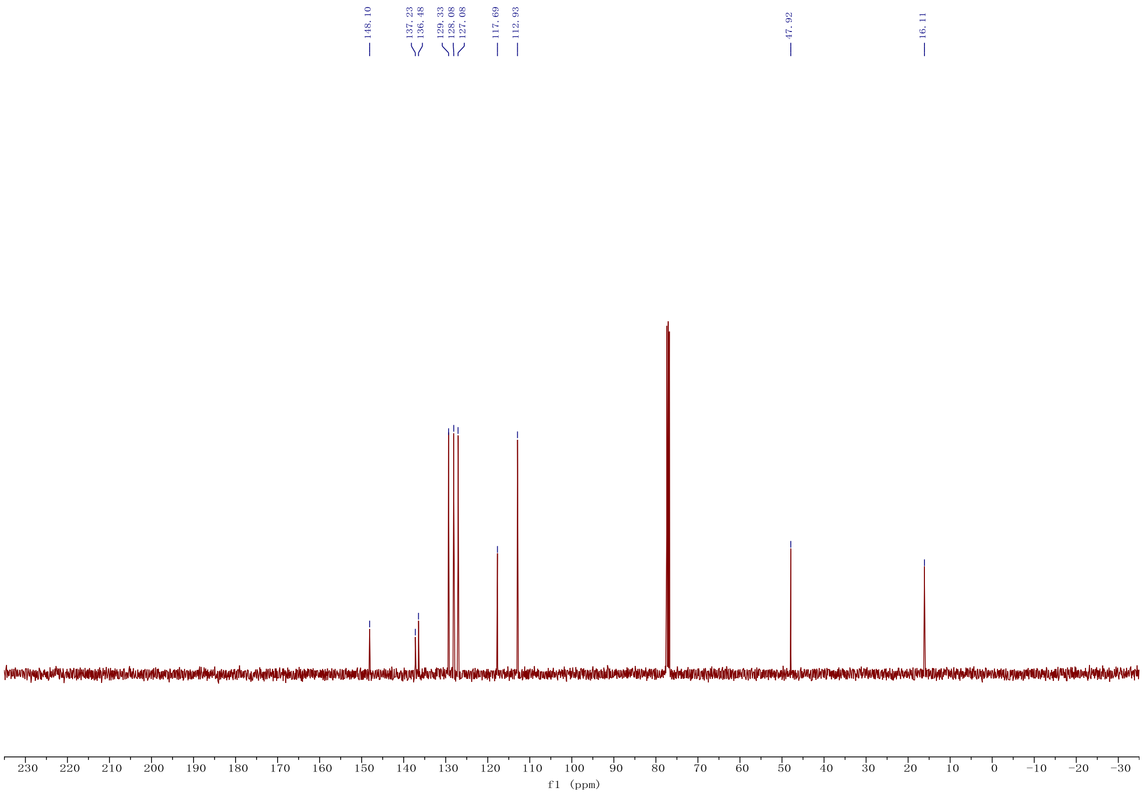

Figure S6-29b. The ^{13}C NMR spectra of **3ja** in Chloroform-*d*.

N-(thiophen-2-ylmethyl)aniline (**3ka**).[8]

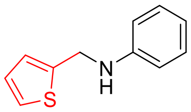

The compound was prepared as described in the general method (oil, 82 % isolated yield, 77 mg). ^{1}H NMR (400 MHz, Chloroform-*d*) δ 7.26 – 7.16 (m, =CH, 3H), 7.08 – 6.97 (m, -C_6H_5, 2H), 6.78 (m, -C_6H_5, 1H), 6.74 – 6.66 (m, -C_6H_5, 2H), 4.55 (s, -CH_2, 2H), 4.10 (m, -NH, 1H) ppm. ^{13}C NMR (101 MHz, Chloroform-*d*) δ = 43.54, 113.22, 118.15, 124.65, 125.10, 126.93, 129.35, 143.02, 147.67 ppm.

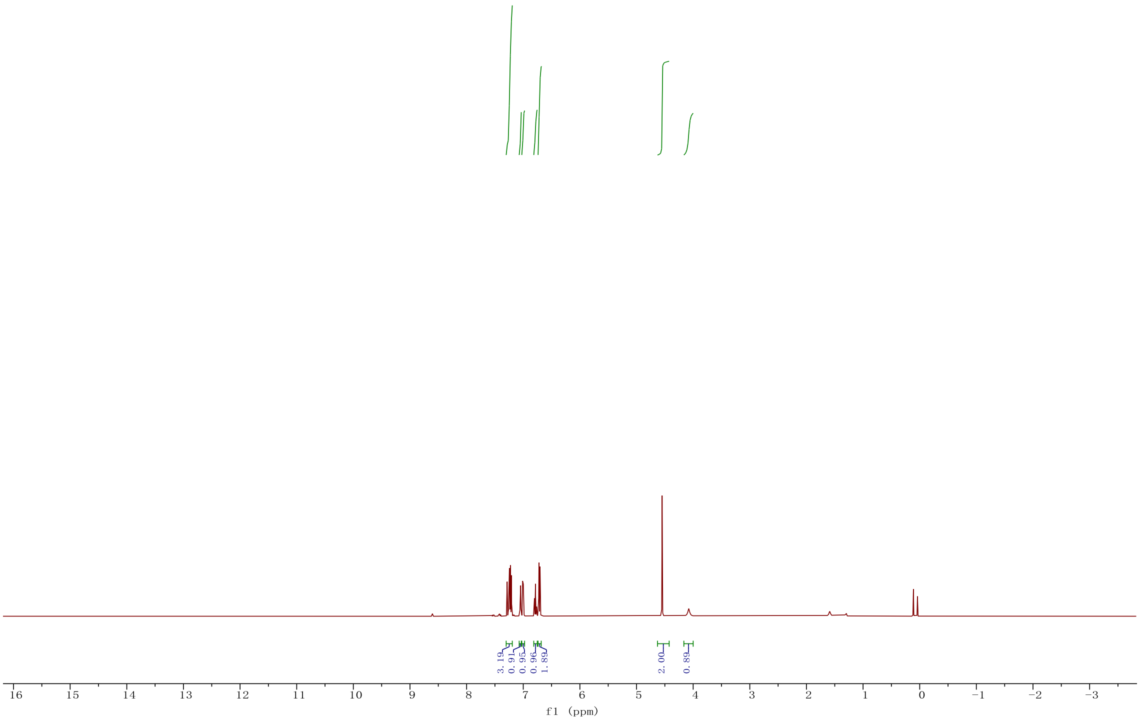

Figure S6-30a. The ^{1}H NMR spectra of **3ka** in Chloroform-*d*.

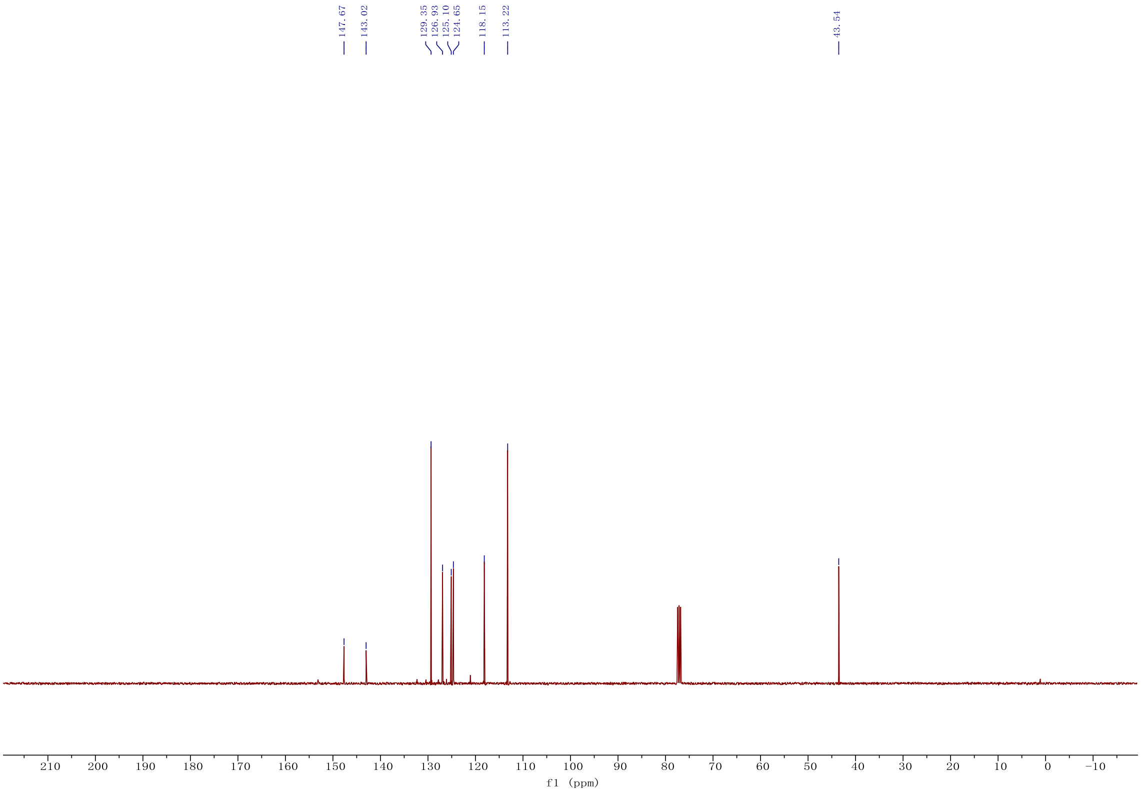

Figure S6-30b. The ^{13}C NMR spectra of **3ka** in Chloroform-*d*.

N-(thiophen-3-ylmethyl)aniline (**3la**).[8]

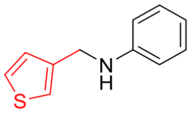

The compound was prepared as described in the general method (oil, 89 % isolated yield, 84 mg). ^{1}H NMR (400 MHz, Chloroform-*d*) δ 7.34 (m, =CH, 1H), 7.26 – 7.18 (m, $-C_6H_5$, =CH, 3H), 7.11 (d, *J* = 5.0 Hz, =CH, 1H), 6.76 (t, *J* = 7.3 Hz, $-C_6H_5$, 1H), 6.69 (d, *J* = 8.0 Hz, $-C_6H_5$, 2H), 4.37 (s, $-CH_2$, 2H), 4.01 (s, -NH, 1H) ppm. ^{13}C NMR (101 MHz, Chloroform-*d*) δ = 43.80, 112.96, 117.74, 121.73, 126.15, 127.17, 129.29, 140.50, 148.06 ppm.

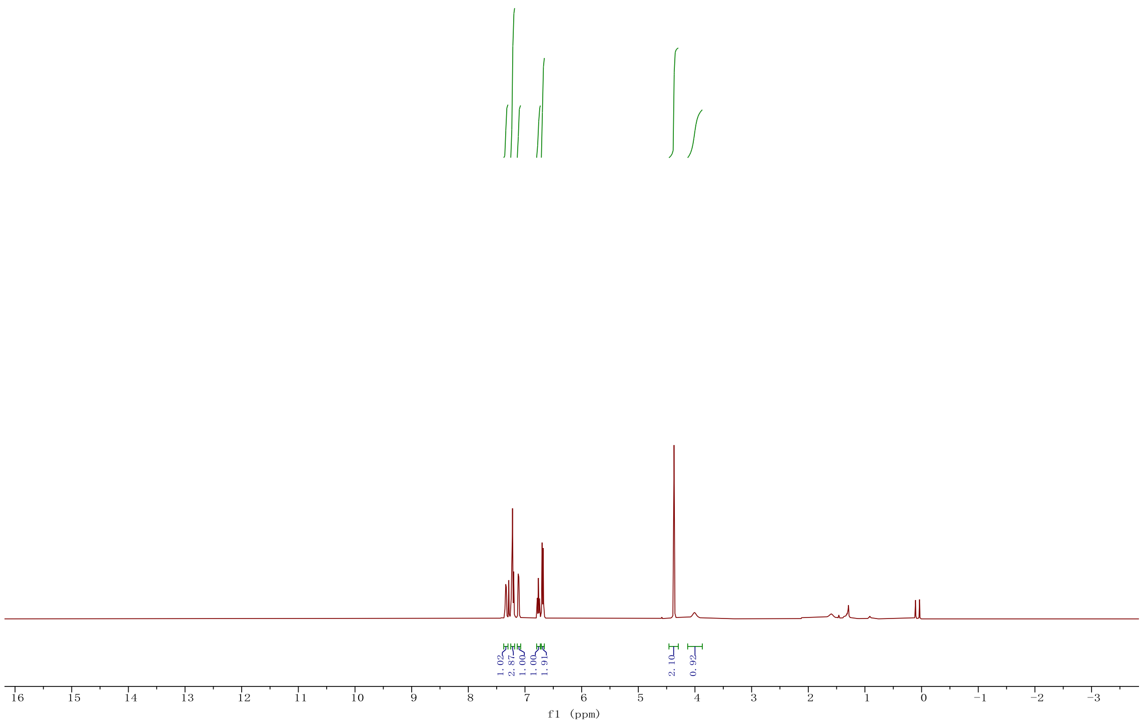

Figure S6-31a. The ^{1}H NMR spectra of **3la** in Chloroform-*d*.

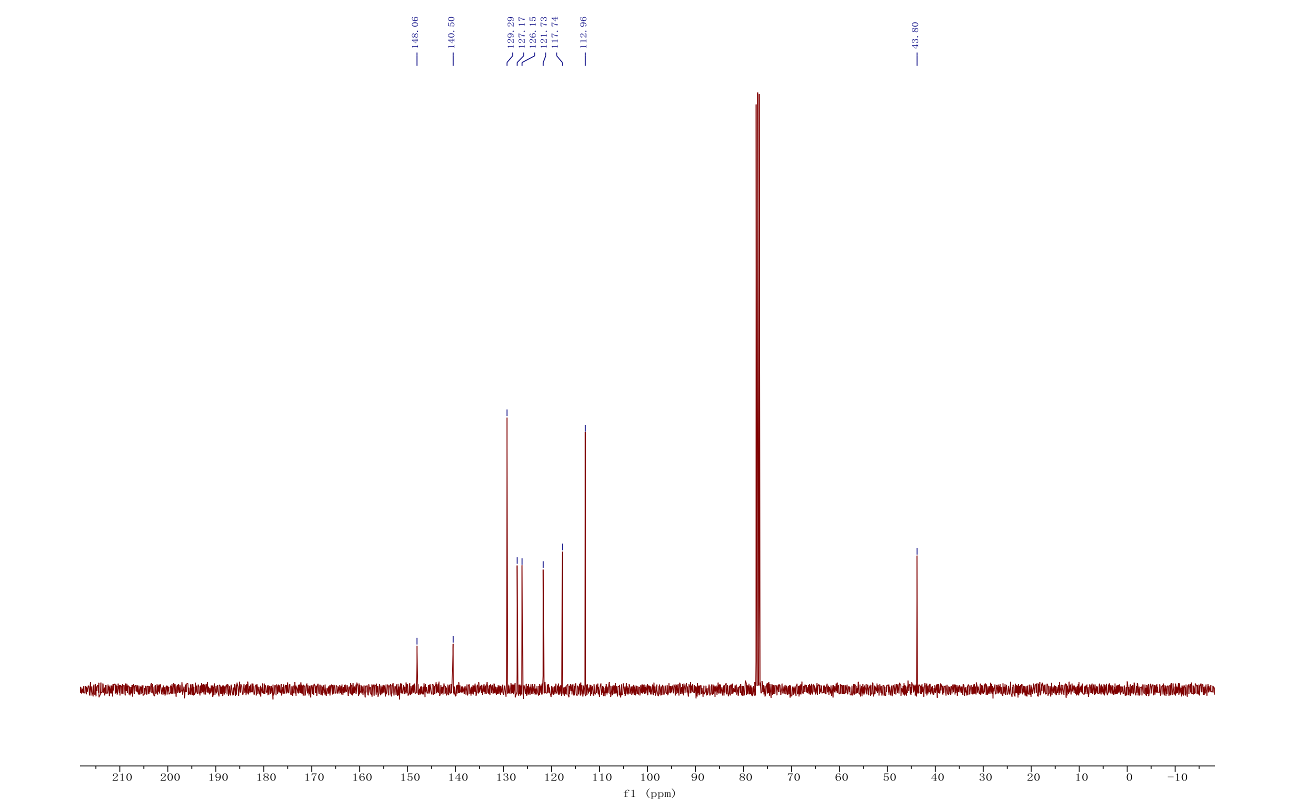

Figure S6-31b. The ^{13}C NMR spectra of **3la** in Chloroform-*d*.

N-(pyridin-3-ylmethyl)aniline (**3ma**).[8]

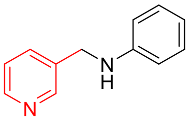

The compound was prepared as described in the general method (solid, 88 % isolated yield, 81 mg). ^{1}H NMR (400 MHz, Chloroform-*d*) δ 8.66 (d, *J* = 2.3 Hz, =CH, 1H), 8.55 (m, =CH, 1H), 7.72 (m, =CH, 1H), 7.33 – 7.25 (m, =CH, 1H), 7.24 – 7.16 (m, -C_6H_5, 2H), 6.81 – 6.73 (m, -C_6H_5, 1H), 6.69 – 6.62 (m, -C_6H_5, 2H), 4.39 (s, -CH_2, 2H), 4.16 (s, -NH, 1H) ppm. ^{13}C NMR (101 MHz, Chloroform-*d*) δ = 45.85, 112.99, 118.09, 123.54, 129.36, 135.09, 147.64, 148.76, 149.21 ppm.

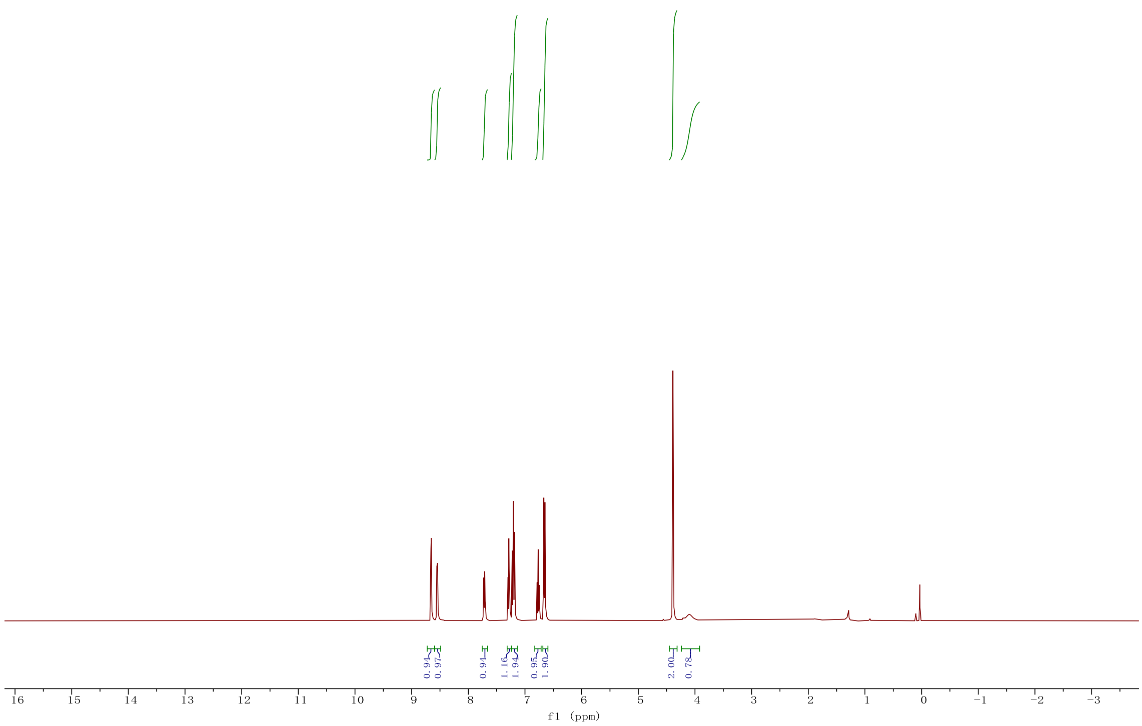

Figure S6-32a. The ^{1}H NMR spectra of **3ma** in Chloroform-*d*.

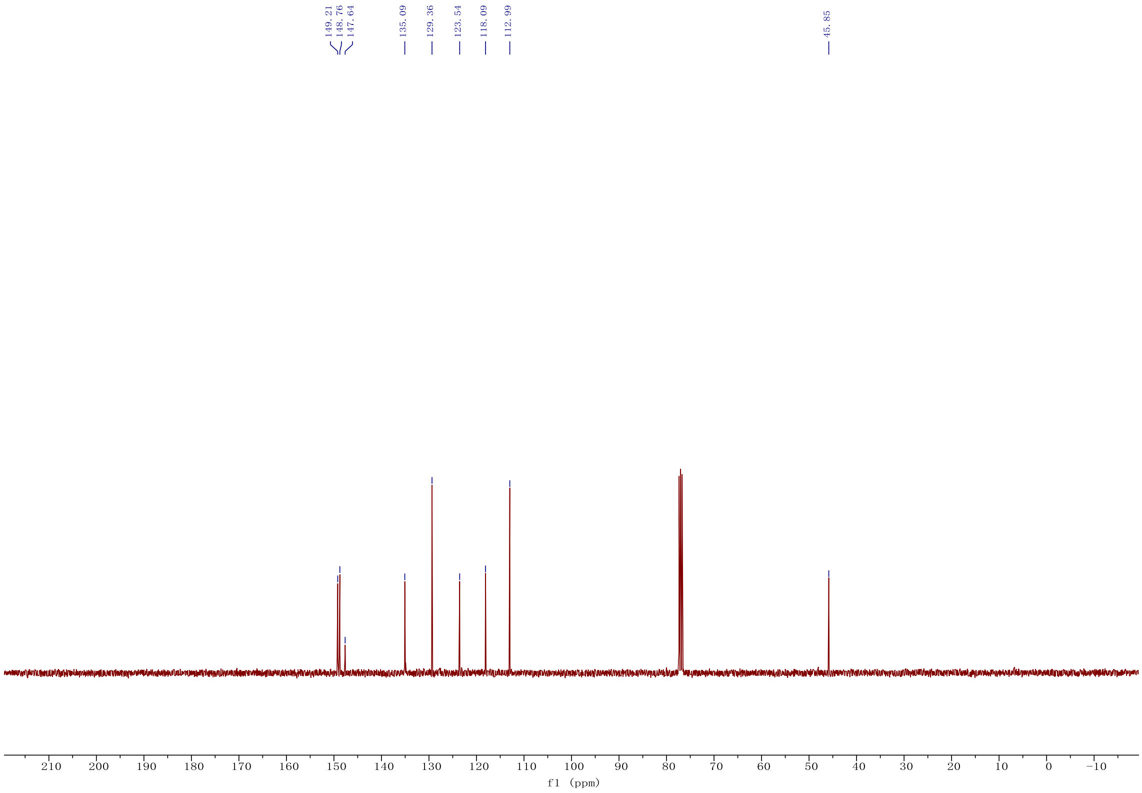

Figure S6-32b. The ^{13}C NMR spectra of **3ma** in Chloroform-*d*.

N-(pyridin-2-ylmethyl)aniline (**3na**).[6]

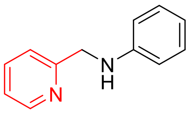

The compound was prepared as described in the general method (oil, 60 % isolated yield, 55 mg). ^{1}H NMR (400 MHz, Chloroform-*d*) δ 8.64 – 8.58 (m, =CH, 1H), 7.66 (m, =CH, 1H), 7.44 – 7.00 (m, -C_6H_5, =CH, 4H), 6.87 – 6.35 (m, -C_6H_5, 3H), 4.77 (s, -NH, 1H), 4.49 (s, -CH_2, 2H) ppm. ^{13}C NMR (101 MHz, Chloroform-*d*) δ = 49.35, 113.09, 117.63, 121.60, 122.09, 129.26, 136.61, 147.93, 149.24, 158.57 ppm.

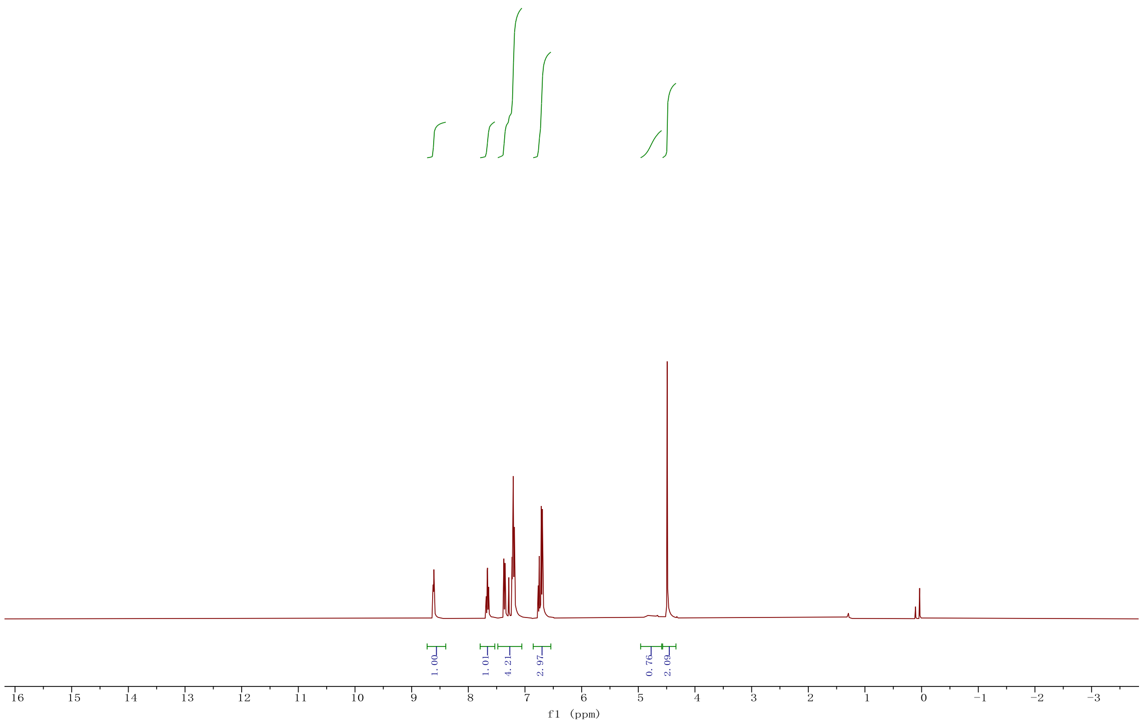

Figure S6-33a. The ^{1}H NMR spectra of **3na** in Chloroform-*d*.

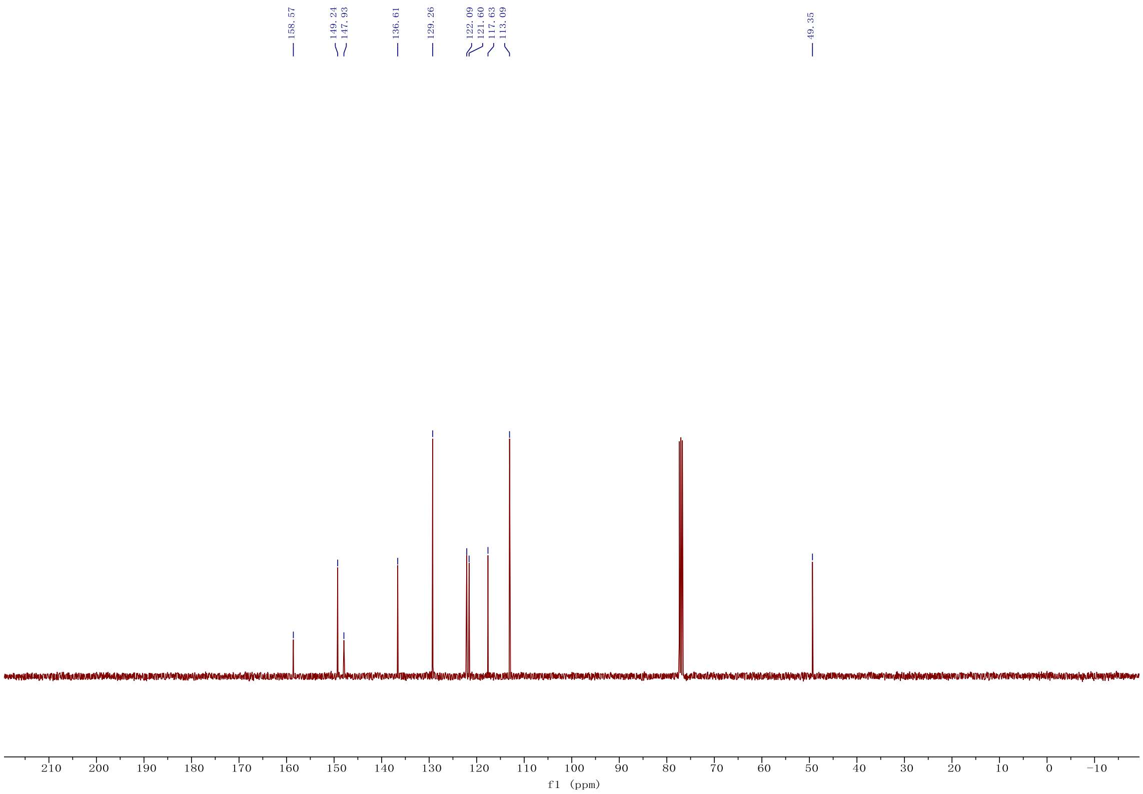

Figure S6-33b. The ^{13}C NMR spectra of **3na** in Chloroform-*d*.

N-(naphthalen-2-ylmethyl)aniline (**3oa**).[6]

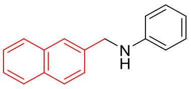

The compound was prepared as described in the general method (oil, 67 % isolated yield, 78 mg). ^{1}H NMR (400 MHz, Chloroform-*d*) δ 7.92 – 7.83 (m, -C_6H_5, 4H), 7.53 (m, -C_6H_5, 3H), 7.26 (m, -C_6H_5, 2H), 6.77 (m, -C_6H_5, 3H), 4.54 (s, -CH_2, 2H), 4.16 (s, -NH, 1H) ppm. ^{13}C NMR (101 MHz, Chloroform-*d*) δ = 48.57, 113.01, 117.71, 125.78, 125.97, 126.20, 127.75, 127.81, 128.42, 129.34, 132.84, 133.57, 137.03, 148.24 ppm.

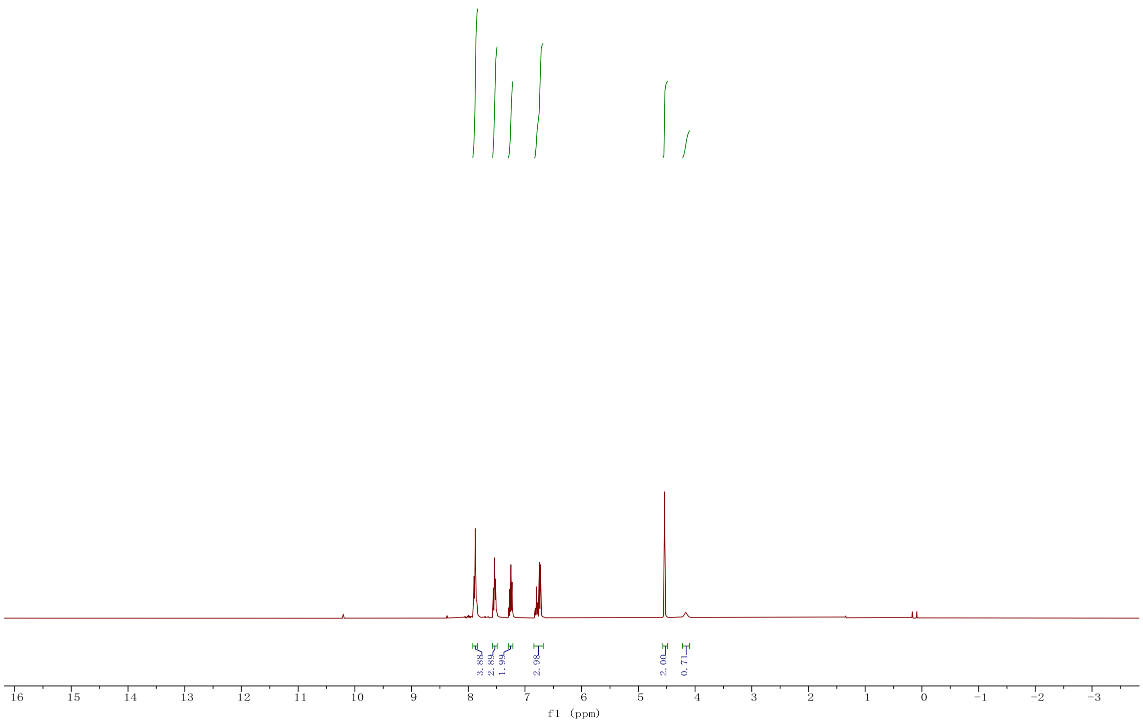

Figure S6-34a. The ^{1}H NMR spectra of **3oa** in Chloroform-*d*.

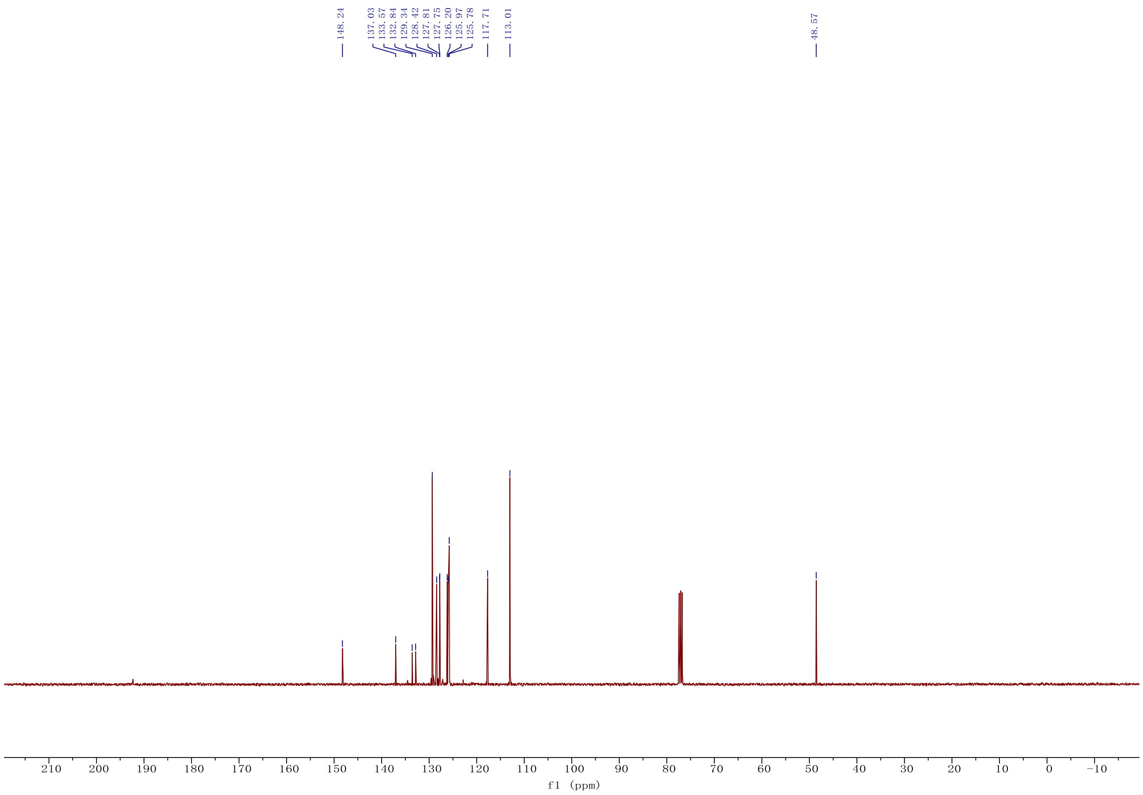

Figure S6-34b. The ^{13}C NMR spectra of **3oa** in Chloroform-*d*.

N-(naphthalen-1-ylmethyl)aniline (**3pa**).[6]

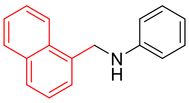

The compound was prepared as described in the general method (oil, 85 % isolated yield, 99 mg). ^{1}H NMR (400 MHz, Chloroform-*d*) δ 8.15 – 8.07 (m, -C_6H_5, 1H), 7.96 – 7.89 (m, -C_6H_5, 1H), 7.84 (d, J = 8.2 Hz, -C_6H_5, 1H), 7.56 (m, -C_6H_5, 3H), 7.46 (m, -C_6H_5, 1H), 7.29 – 7.20 (m, -C_6H_5, 2H), 6.83 – 6.70 (m, -C_6H_5, 3H), 4.78 (s, -CH_2, 2H), 4.08 (s, -NH, 1H) ppm. ^{13}C NMR (101 MHz, Chloroform-*d*) δ = 45.95, 113.71, 117.65, 123.58, 125.54, 125.84, 126.09, 126.34, 128.20, 128.77, 129.33, 131.57, 133.91, 134.91, 147.14 ppm.

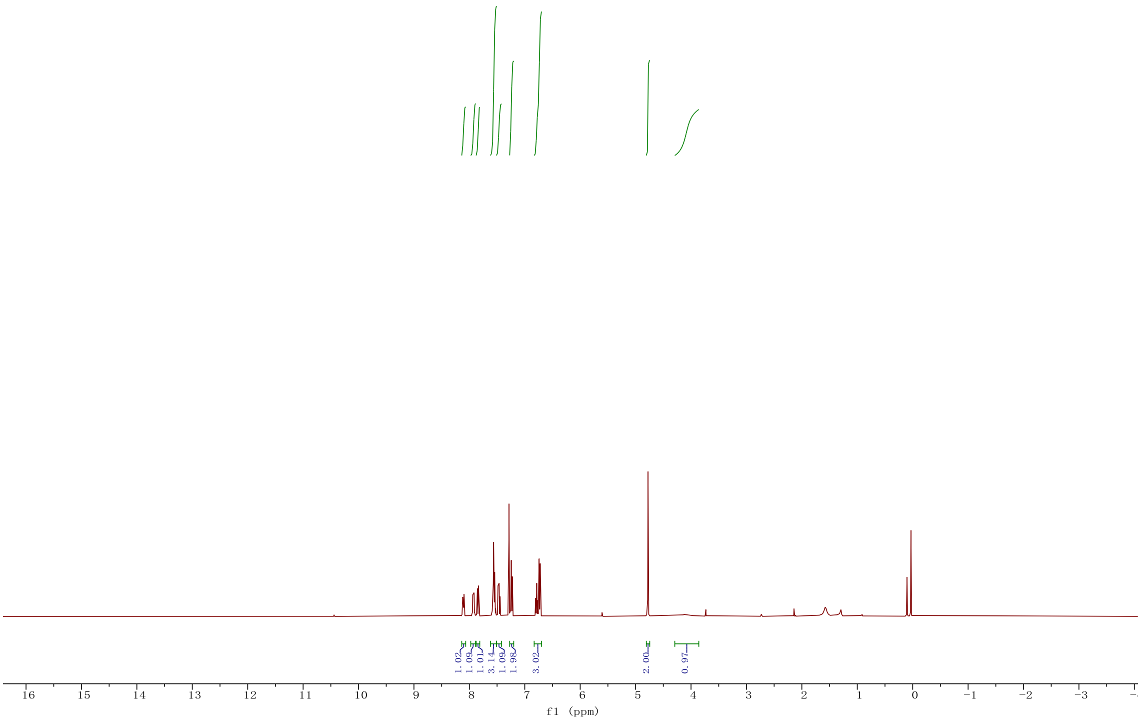

Figure S6-35a. The ^{1}H NMR spectra of **3pa** in Chloroform-*d*.

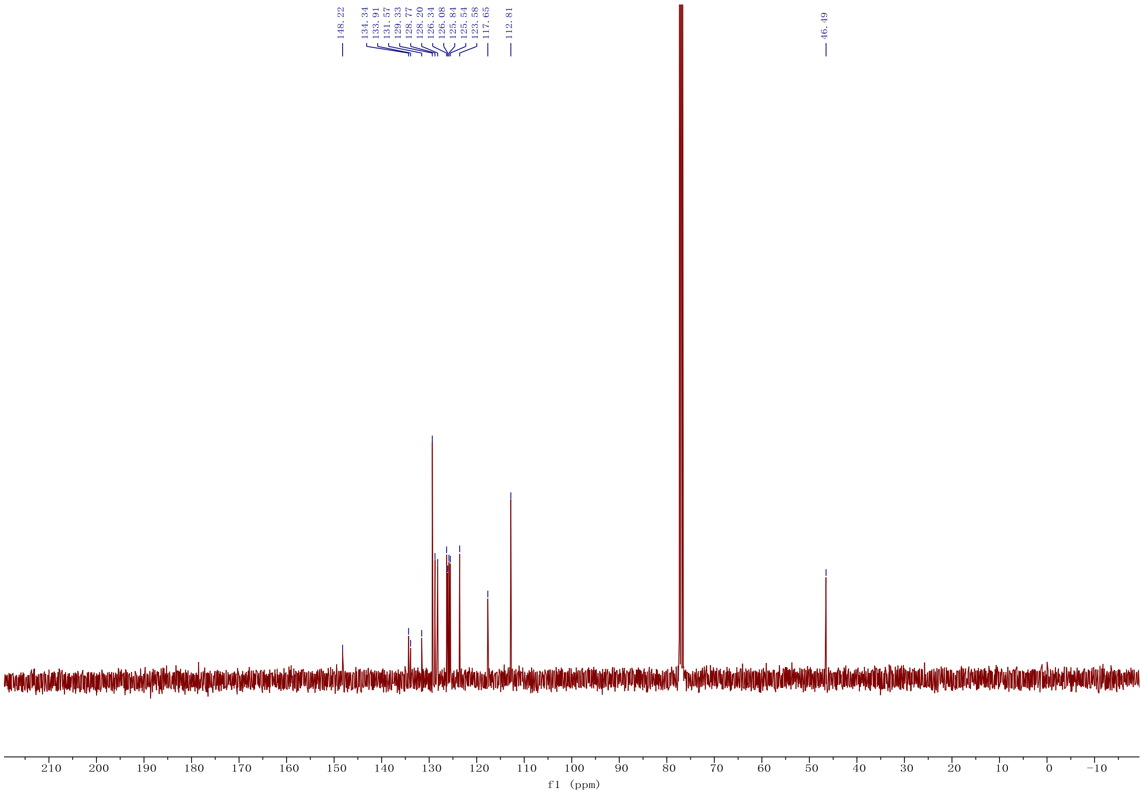

Figure S6-35b. The ^{13}C NMR spectra of **3pa** in Chloroform-*d*.

N-([1,1'-biphenyl]-4-ylmethyl)aniline (**3qa**).[6]

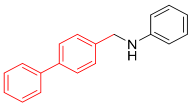

The compound was prepared as described in the general method (soild, 82 % isolated yield, 107 mg). ^{1}H NMR (400 MHz, Chloroform-*d*) δ 7.71 – 7.58 (m, -C_6H_5, 4H), 7.48 (m, -C_6H_5, 4H), 7.42 – 7.34 (m, -C_6H_5, 1H), 7.27 – 7.19 (m, -C_6H_5, 2H), 6.77 (m, -C_6H_5, 1H), 6.73 – 6.67 (m, -C_6H_5, 2H), 4.42 (s, -CH_2, 2H), 4.11 (s, -NH, 1H) ppm. ^{13}C NMR (101 MHz, Chloroform-*d*) δ = 48.02, 112.89, 117.65, 127.10, 127.30, 127.42, 127.95, 128.82, 129.33, 138.54, 140.24, 140.87, 148.14 ppm.

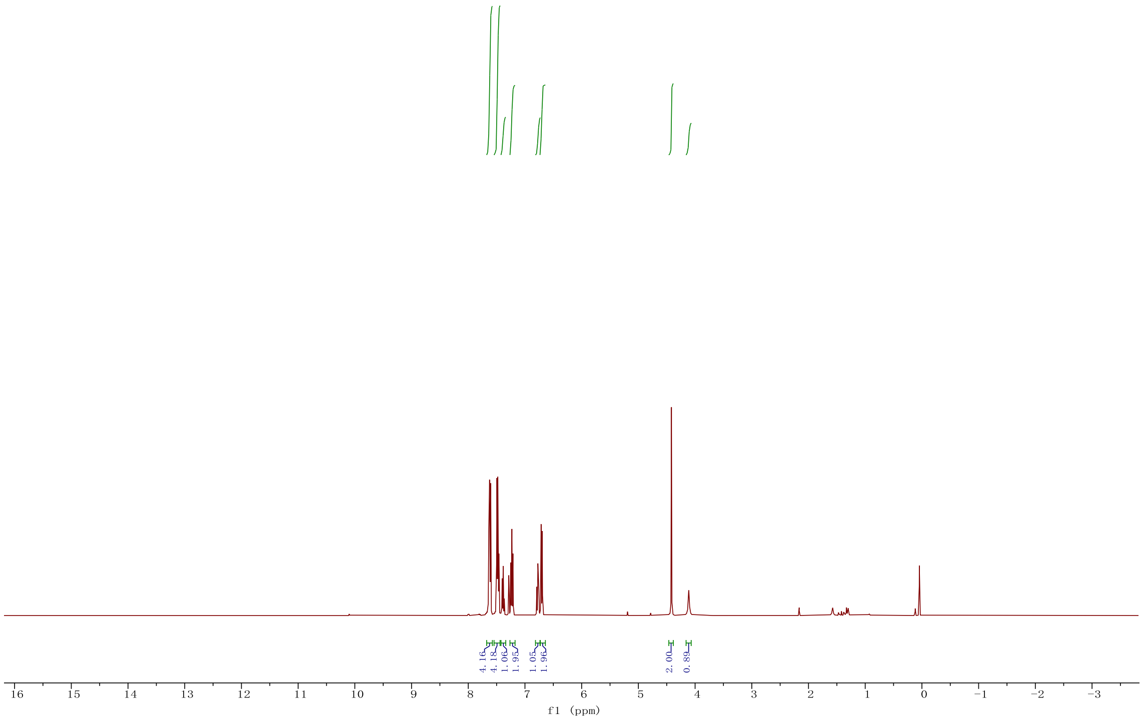

Figure S6-36a. The ^{1}H NMR spectra of **3qa** in Chloroform-*d*.

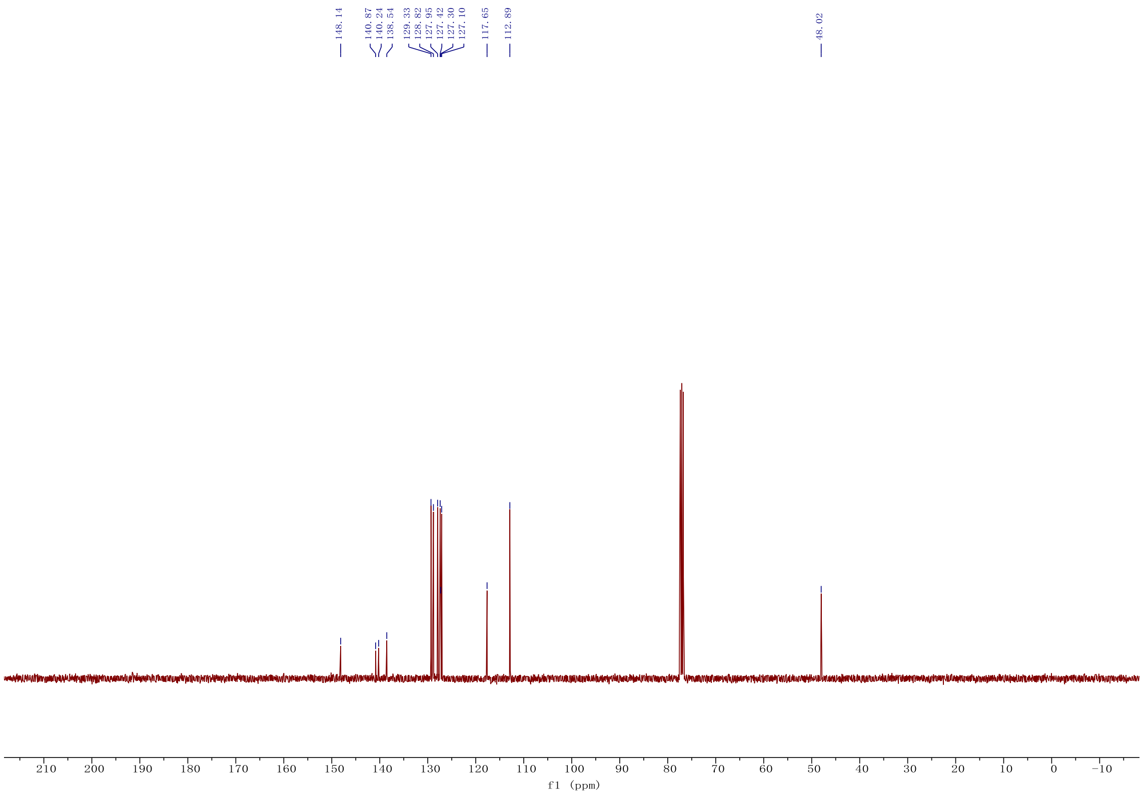

Figure S6-36b. The ^{13}C NMR spectra of **3qa** in Chloroform-*d*.

N-(3,7-dimethyloct-6-en-1-yl)aniline (**3ra**).[12]

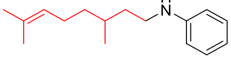

The compound was prepared as described in the general method (oil, 78 % isolated yield, 90 mg). ^{1}H NMR (400 MHz, Chloroform-*d*) δ 7.26 – 7.13 (m, -C_6H_5, 2H), 6.73 (t, *J* = 7.3 Hz, -C_6H_5, 1H), 6.68 – 6.62 (m, -C_6H_5, 2H), 5.15 (m, =CH, 1H), 3.58 (s, -NH, 1H), 3.17 (m, -CH_2, 2H), 2.15 – 1.95 (m, -CH_2, 2H), 1.74 (d, *J* = 1.6 Hz, -CH_3, 3H), 1.70 – 1.60 (m, -CH_2, 5H), 1.53 – 1.17 (m, -CH_3, 3H), 1.05 – 0.87 (m, -CH_3, 3H) ppm. ^{13}C NMR (151 MHz, Chloroform-*d*) δ = 17.68, 19.63, 25.51, 25.73, 30.48, 36.76, 37.13, 41.98, 112.73, 117.12, 124.70, 129.23, 131.32, 148.60 ppm.

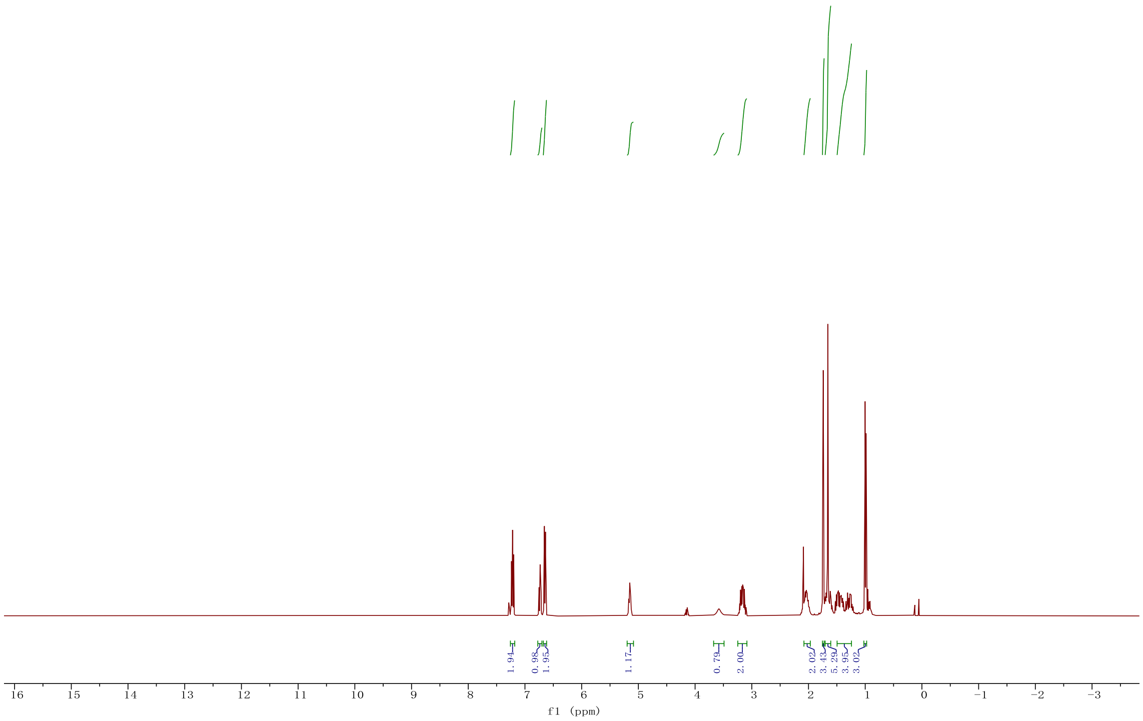

Figure S6-37a. The ^{1}H NMR spectra of **3ra** in Chloroform-*d*.

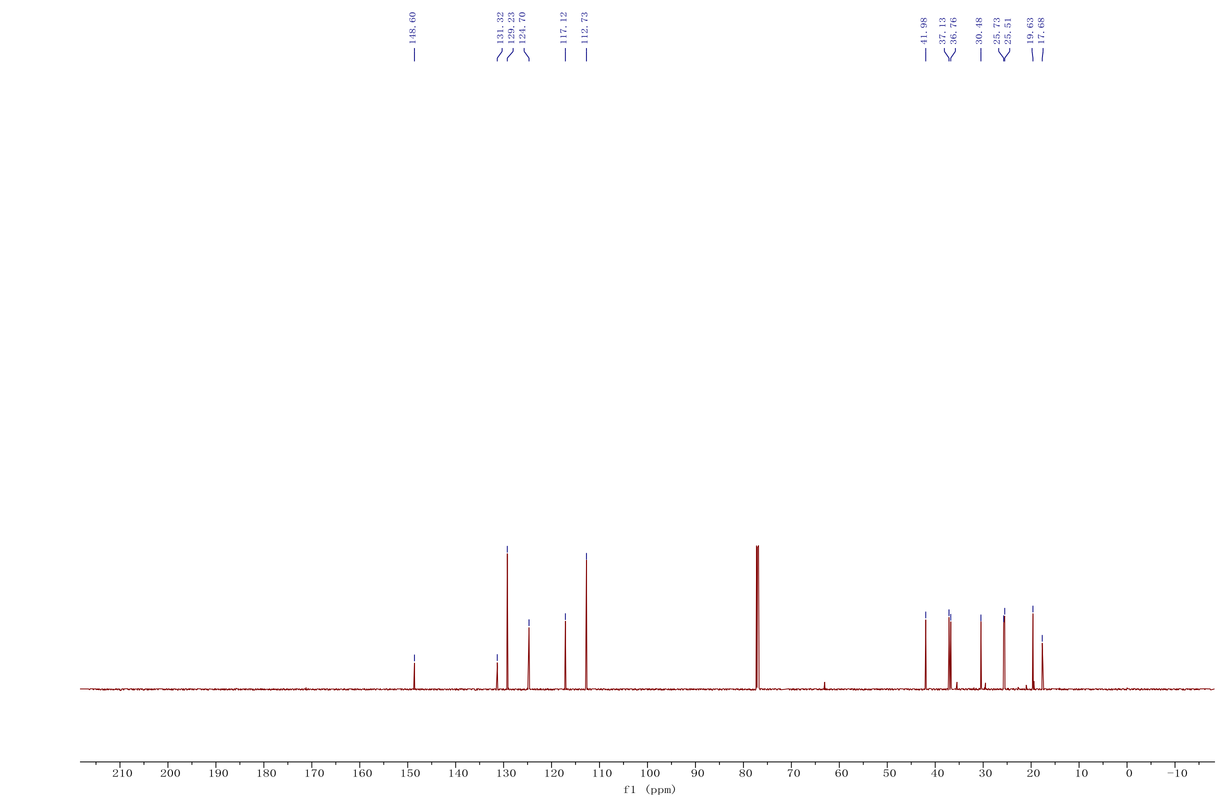

Figure S6-37b. The ^{13}C NMR spectra of **3ra** in Chloroform-*d*.

4-((3-(trifluoromethyl)benzyl)amino)benzonitrile (**3sa**).

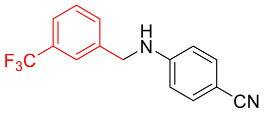

The compound was prepared as described in the general method (oil, 61 % isolated yield, 84 mg). ^{1}H NMR (400 MHz, Chloroform-d) δ 7.52 (m, -C_6H_5, 4H), 7.42 (d, -C_6H_5, 2H), 6.59 (d, J = 8.3 Hz, -C_6H_5, 2H), 4.73 (s, -NH, 1H), 4.45 (d, J = 4.7 Hz, -CH_2, 2H) ppm. ^{13}C NMR (101 MHz, Chloroform-*d*) δ 47.12, 99.76, 112.59, 120.23, 123.95, 124.62, 129.43, 130.50, 133.84, 139.06, 150.82 ppm.

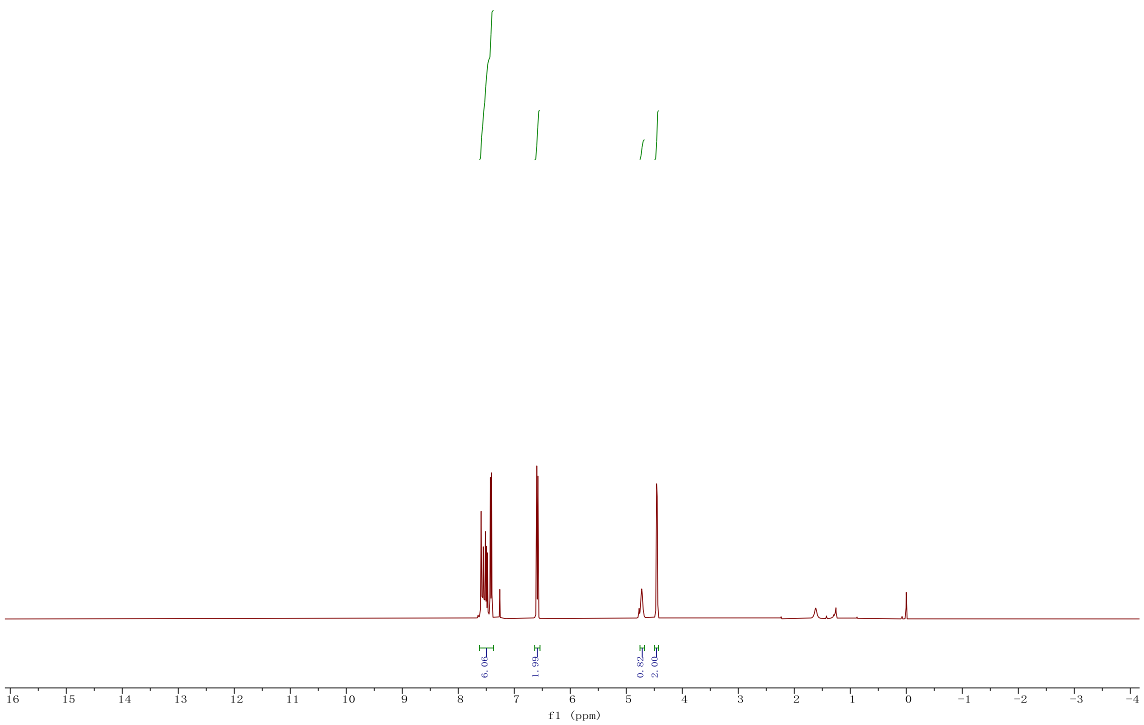

Figure S6-38a. The ^{1}H NMR spectra of **3sa** in Chloroform-*d*.

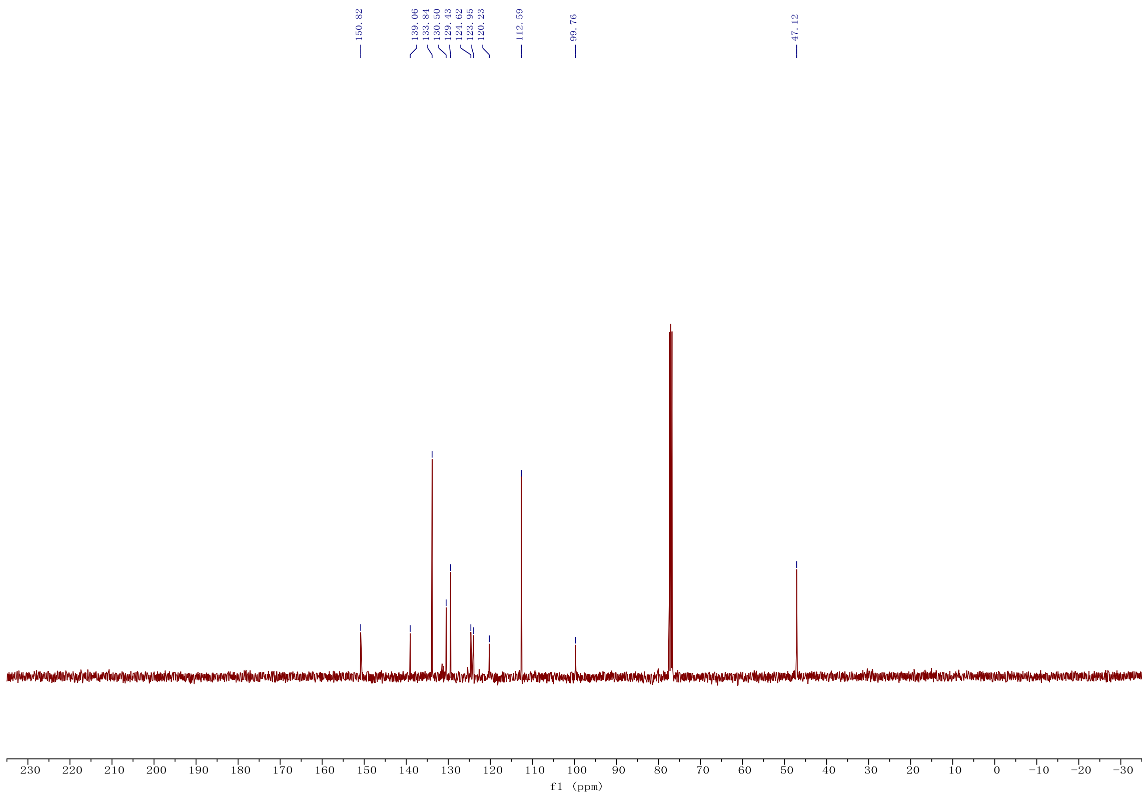

Figure S6-38b. The ^{1}H NMR spectra of **3sa** in Chloroform-*d*.

IV. Mechanism Details

The *bis*-NHC achieving silver-catalyzed transformation stimulates us to further investigate the silver (de)hydrogenation reaction mechanism. To gain more insights, preliminary control experiments were conducted. Three drops of Hg were added to the reaction under optimal conditions. The reaction still resulted in a satisfactory yield (83%), indicating the homogenous nature of this **NHC-Ag** catalysis (**Figure 3B**, entry 1). A radical process could be ruled out with the observation of a high yield (85% and 84%) in the presence of stoichiometric amounts of the radical scavengers, TEMPO and BHT (**Figure 3B**, entry 2), respectively. To further illustrate the existence of silver-hydride species in the *N*-alkylation reaction, verification experiments were performed. A significant concentration of hydrogen gas was detected during the reaction by GC analysis, produced by the silver-hydride species (**Figure S7**). The hydrogen-deuterium exchange was observed for the *N*-alkylation between **1a-D** and **2a** under the optimal reaction conditions. The **3aa**, **3aa-*d*₁**, and **3aa-*d*₂** ratios were calculated based on the detected ^{1}H NMR spectra (14:42:44, **Figure 3B**, entry 3, and **Figure S8**). Furthermore, the kinetic isotope effect (KIE = 2.15) was measured by using benzyl alcohol-α-d_2 (**1a-D**), which indicated that the (de)hydrogenation of alcohols was probably involved in the rate-determining step (RDS) along the catalytic cycle. Meanwhile, substrate **1b**, benzaldehydes **4a**, and **2a** were added in a molar ratio of 1:1:1 under optimal reaction conditions. The yields of 28% for **3aa** and 36% for **3ba** were detected by GC, which further confirmed the realization of silver-catalyzed BH/HA reaction (**Figure 3B**, entry 4). Ensuring the silver achieved the BH/HA process, the kinetics of the silver catalytic system were carried out to establish a catalytic model (**Figure 3C**). The reaction was found to be 0.5 order concerning **NHC-Ag**, indicating the dissociation of di-nuclear **NHC-Ag** in the reaction cycle. Meanwhile, the reaction order of ligand **L2** was found to be one, which confirmed that one *bis*-NHC ligand was involved in the catalytic cycle (**Figure S9-3b**). Combined together, these results revealed the final mechanism study model in which the catalytic species was likely composed of only one silver center and one *bis*-NHC ligand.

1. Detection of hydrogen gas

To a 15 mL reaction tube in a glovebox, was added aniline (**2a**, 0.5 mmol), benzyl alcohol (**1a**, 1.0 mmol), ^{t}BuOK (0.7 equiv.), **NHC-Ag** (2.5 mol%), and 1,4-dioxane (1 mL). Then the tube was closed with a rubber stopper sealed with iron wire and removed from the glovebox. The reaction mixture was stirred for 2 h at 150 °C. The head gas was collected by a gas-tight syringe and analyzed by GC. Hydrogen gas was detected.

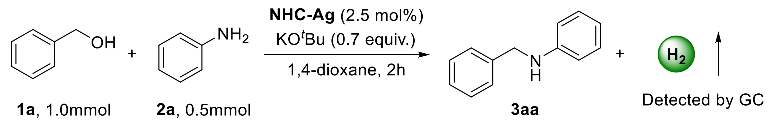

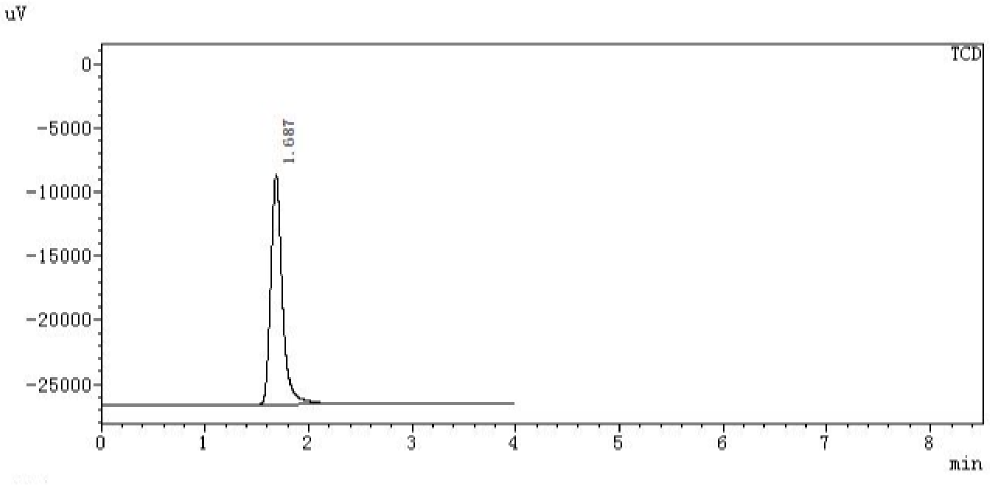

Figure S7. Detection of hydrogen gas by GC. GC parameters: injection temperature = 200 °C, column temperature = 60 °C, detector temperature = 150 °C. 5 Å molecular sieves column was used, and the carrier gas was N_2. The retention time for H_2 was 1.687 min.

2. Deuterium-labelling experiment

Following the general method[6] to prepare the benzylalcohol-α-d_2 (**1a-D**). To a 10 mL seal tube in a glovebox, was added aniline (**2a**, 0.5 mmol), benzyl alcohol-α-d_2 (**1a-D**, 1.0 mmol), ^{t}BuOK (0.7 equiv.), **NHC-Ag** (2.5 mol%) and 1,4-dioxane (1 mL). Then the tube was sealed with a Teflon plug and removed from the glovebox. The reaction mixture was stirred for 24 h at 150 °C. After cooling to R.T., the crude reaction mixture was diluted with 2 mL CH_3OH and 5 mL EtOAc in order. The solvent was evaporated to dryness and the corresponding amine was purified by column chromatography with silica gel (ethyl acetate/petroleum ether).

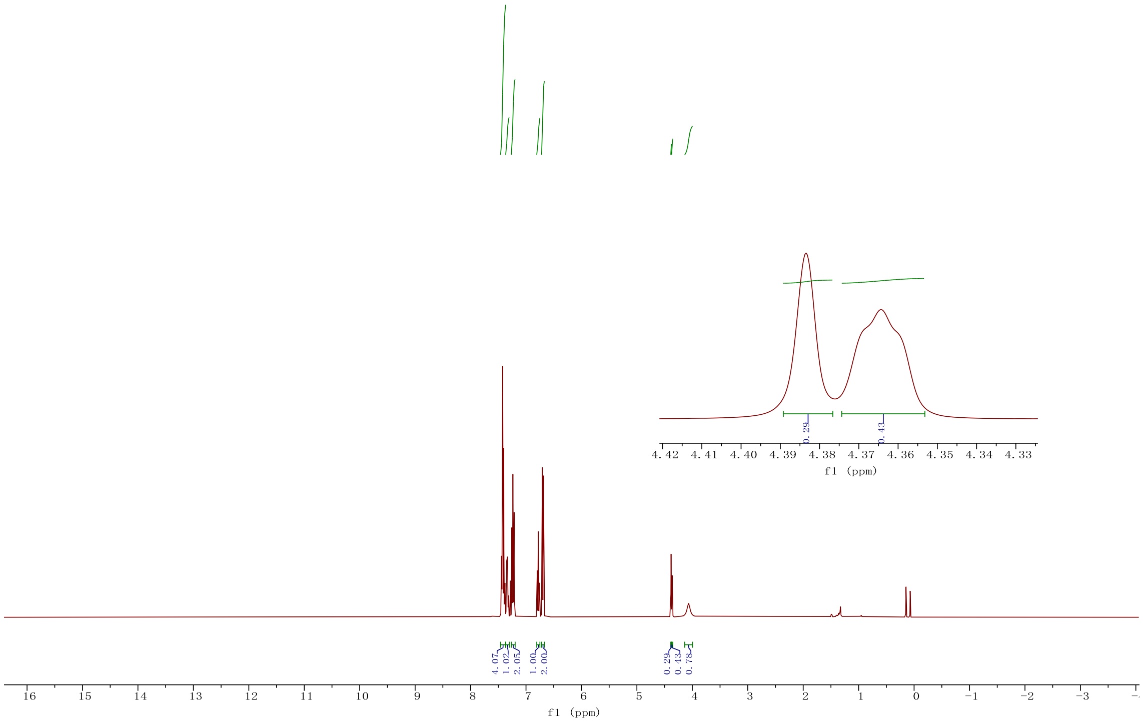

Figure S8. The ^{1}H NMR spectra of **3aa-D** in Chloroform-*d*.

3. Kinetic isotope effect (KIE) studies

The kinetic isotope effect (KIE) studies were carried out to gain more information concerning the reaction pathway. Two parallel reactions, one with benzyl alcohol (**1a**) and another with benzyl alcohol-*α*-d_2 (**1a-D**), were carried out. To a 15 mL reaction tube in a glovebox, was added aniline (0.5 mmol), alcohol (1.0 mmol), KO^{t}Bu (0.7 equiv.), **NHC-Ag** (2.5 mol%) and dodecane (113 uL) in 1,4-dioxane (1 mL). Then the tube was closed with a rubber stopper sealed with iron wire and removed from the glovebox. The reaction mixture was stirred at 150 °C, the aliquots were taken after 10, 20, 30, 40, 50, 60 and 70 min. And yields of products were determined by GC. The calculated KIE (k_H/k_D) was 2.15.

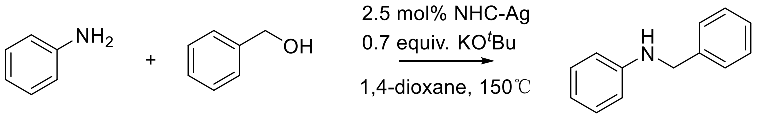

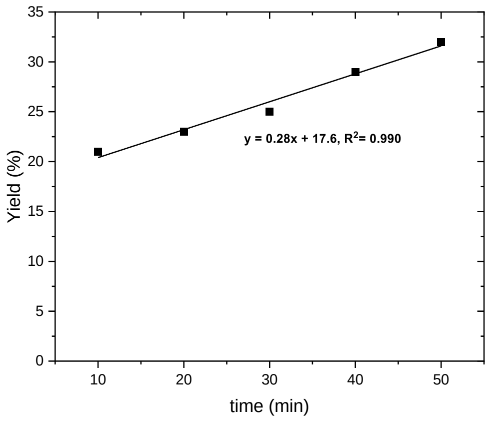

Figure S9-1. Correlation between time and GC yield.

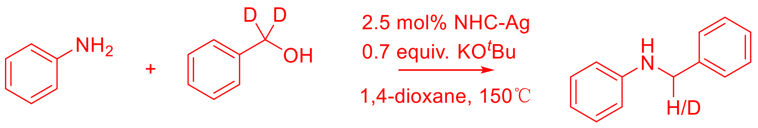

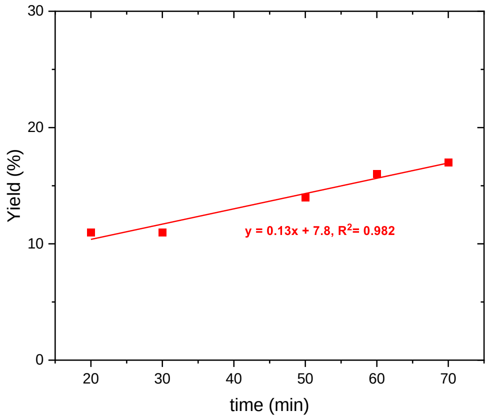

Figure S9-2. Correlation between time and GC yield.

4. Reaction order of silver catalyst and ligand 6

Two situ reactions, one with **NHC-Ag** and another with **L6**, were carried out. To a 15 mL reaction tube in a glovebox, was added aniline (0.5 mmol), alcohol (1.0 mmol), KO^{t}Bu (0.7 equiv.), **NHC-Ag** and dodecane (113 uL) in 1,4-dioxane (1 mL). To a 15 mL reaction tube in a glovebox, was added aniline (0.5 mmol), alcohol (1.0 mmol), KO^{t}Bu (0.7 equiv.), **L6** and dodecane (113 uL) in 1,4-dioxane (1 mL). Then the tubes were closed with rubber stoppers sealed with iron wire and removed from the glovebox. The reaction mixture was stirred at 150 ^{o}C, the aliquots were taken after 10, 20, 30, 40, 50, 60 and 70 min. And yields of products were determined by GC. The order of **NHC-Ag** was 0.5, and the order of **L6** was 1.0.

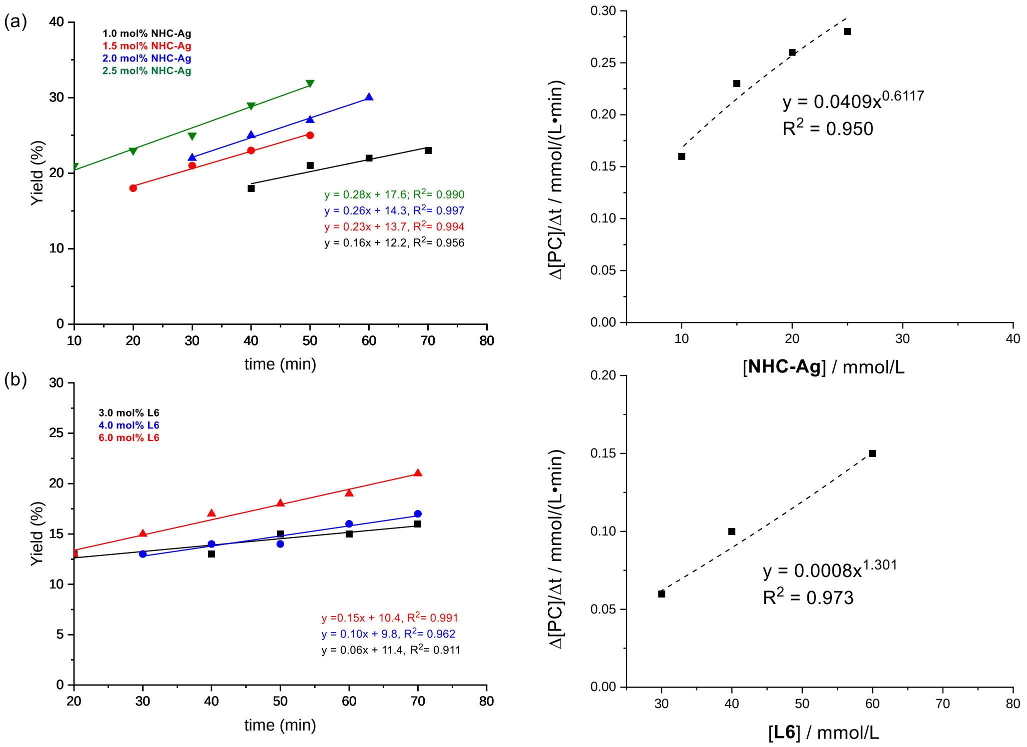

Figure S9-3. (a) Order of **NHC-Ag**. (b) Order of **L6**.

5. Detection of the dissociative functional arm by ^{1}H NMR

Four experiment tests were carried out, respectively. Test 1 was the ^{1}H NMR of *bis*-NHC in DMSO-d_6. Test 2 was the ^{1}H NMR of **NHC-Ag** in DMSO-d_6. The **NHC-Ag** (0.06 mmol) and KO^{t}Bu (0.125 mmol) were added to 1 mL 1,4-dioxane for Test 3. The solvent was removed after stirring 1 h at R.T., and the ^{1}H NMR in DMSO-d_6 was detected. The **NHC-Ag** (0.06 mmol), KO^{t}Bu (0.125 mmol), and benzyl alcohol (**1a**, 0.125 mmol) were added to 1 mL 1,4-dioxane for Test 4. The solvent was removed after stirring 2 h at 150 °C under Ar atmosphere and detected the ^{1}H NMR in DMSO-d_6.

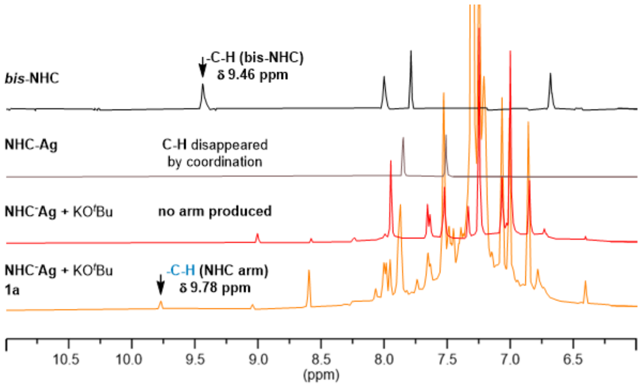

Figure S9-4. Detection of the dissociative functional arm by ^{1}H NMR.

6. Gram-scale reaction

The gram-scale reaction was carried out. To a 150 mL seal tube in a glovebox, was added aniline (8 mmol), alcohol (16 mmol), KO^{t}Bu (0.7 equiv.), **NHC-Ag** (2.5 mol%) in 1,4-dioxane (16 mL). The reaction mixture was stirred for 24 h at 150 °C. After cooling to R.T., the crude reaction mixture was diluted with CH_3OH. The solvent

was evaporated to dryness and the corresponding amine was purified by column chromatography with silica gel (ethyl acetate/petroleum ether).

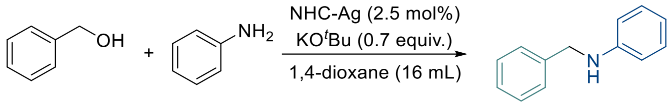

1a, 16.0 mmol **2a**, 8 mmol **3aa**, 73%, 1.07g

Figure S9-5. Gram-scale reaction.

V. Computational Details

All of the geometry optimizations and frequency calculations used the mixed basis sets with M06-L/BSI level[14-17] (BSI designated the basis set combination of Lanl2DZ for Ag, and 6-31G** for other atoms) in density functional theory (DFT) calculations.[18-20] All of the reactants, intermediates and products had no imaginary frequency, and the transition states had only one imaginary frequency. Intrinsic reaction coordinate (IRC) computations were used to confirm the structure of transition states. The energetic results were then further refined by single-point calculations at M06-L/BSII level[21,22] (BSII designated the basis set combination of SDD for Ag, and 6-311++G** for other atoms). The solvation effects of 1,4-dioxane (ε = 2.2099) were simulated by the SMD[23] solvent model. All 3D diagrams of the structures were generated by CYLview[24] and IQmol[25]. The Multiwfn program[26] were employed to analyze the orbital components

Cartesian coordinates

NHC-Ag

N	2.18559	1.52963	2.15504
N	1.59617	2.47711	0.33600
N	-0.12582	3.22428	-1.18451
N	-2.24836	3.46477	-1.17277
N	-1.20214	-1.21263	2.83369
N	-1.46705	-2.32565	1.03331
N	-0.41747	-3.16642	-0.95002
N	1.59705	-3.31515	-1.65062
C	1.70203	1.25629	0.92472
C	1.19361	2.63207	-1.03737
H	1.91382	3.25894	-1.56796
H	1.20978	1.63443	-1.48498
C	-1.27352	2.62386	-0.75636
C	-1.40682	-1.05173	1.50463
C	-1.64243	-2.63386	-0.36594
H	-2.43817	-3.36990	-0.48970
H	-1.95881	-1.71677	-0.86912
C	0.76623	-2.49395	-0.96802
C	-3.67066	3.20448	-1.00501
H	-4.17091	4.11413	-0.66806
H	-4.10860	2.86838	-1.94563
H	-3.80893	2.41034	-0.27334
C	2.99897	-3.00796	-1.89400
H	3.15875	-1.93722	-1.78225
H	3.63895	-3.52041	-1.17312
H	3.27002	-3.31260	-2.90576
C	-1.11670	-0.10788	3.76645
H	-0.88219	0.79765	3.20214
H	-2.06607	0.03413	4.28842
H	-0.32618	-0.29458	4.49674
C	2.70089	0.49590	3.03177
H	2.13652	-0.42243	2.85368
H	3.75033	0.30957	2.79166
H	2.59176	0.80058	4.07468
C	2.40666	2.88472	2.32616
C	2.02468	3.49059	1.17560
C	-0.38403	4.40621	-1.85383
C	-1.73262	4.55494	-1.84601
C	-1.13995	-2.55051	3.18626
C	-1.30438	-3.25700	2.04056
C	-0.32425	-4.37816	-1.60759
C	0.95632	-4.47060	-2.05049
H	2.82427	3.29175	3.23324
H	2.01635	4.52983	0.88720
H	0.39953	5.01876	-2.27194
H	-2.36211	5.32890	-2.25514
H	-1.00015	-2.87539	4.20500

H	-1.31945	-4.31992	1.85794
H	-1.16718	-5.04231	-1.71797
H	1.45796	-5.24445	-2.60920
Ag	-1.49501	0.76806	0.32170
Ag	1.30685	-0.64465	-0.03376
P	-4.87177	-0.40383	-0.40917
F	-6.20265	-0.66251	0.45662
F	-5.35794	-1.38646	-1.59330
F	-4.28151	0.60003	0.78110
F	-3.45615	-0.11289	-1.26309
F	-5.54111	0.88255	-1.14017
F	-4.11755	-1.65909	0.33483
P	4.90016	0.07361	-0.47659
F	6.17336	-0.23575	0.46489
F	5.73655	1.09465	-1.40280
F	5.27025	-1.17364	-1.45520
F	3.53616	0.36123	-1.40295
F	3.97436	-0.95867	0.45025
F	4.45608	1.30562	0.50834

NHC-Ag-RC

N	-2.44579	2.23623	-0.28914
N	-1.56858	1.58229	1.54393
N	-0.10324	-0.26563	1.99178
N	1.16365	-1.66298	0.93370
C	-1.25025	1.98229	0.27412
C	-0.60346	1.02673	2.45684
H	0.26012	1.69078	2.54825
H	-1.07275	0.90438	3.43492
C	1.13173	-0.44978	1.48406
C	2.31631	-2.18412	0.20901
H	2.98090	-2.72936	0.88221
H	1.95927	-2.85103	-0.57466
H	2.83860	-1.32869	-0.22926
C	-2.63974	2.45847	-1.70917
H	-1.67195	2.67597	-2.16100
H	-3.05902	1.54770	-2.14335
H	-3.31789	3.29825	-1.87225
H	1.95427	0.26345	1.41159
Ag	0.74368	1.79651	-0.39868
O	2.68383	1.03007	-0.25887
C	3.83847	1.31589	-0.97686
H	3.67685	1.33718	-2.07207
H	4.25517	2.31130	-0.71762
C	4.86753	0.25547	-0.66667
C	5.31454	0.09555	0.65060
C	5.30872	-0.65043	-1.63238
C	6.18089	-0.93652	0.99138
H	4.96433	0.79309	1.40956
C	6.17545	-1.69049	-1.29687
H	4.95998	-0.54076	-2.65805
C	6.61209	-1.83742	0.01634
H	6.52562	-1.04168	2.01728
H	6.50742	-2.38780	-2.06185
H	7.28802	-2.64621	0.28052
C	-0.87597	-1.38015	1.72788
C	-0.07341	-2.25868	1.07323
C	-2.94050	1.56876	1.74271
C	-3.48924	1.98621	0.58136
H	-0.30873	-3.21462	0.63762
H	-1.94199	-1.41041	1.90502
H	-3.39319	1.23182	2.66088
H	-4.51888	2.05835	0.27605
P	-3.54897	-1.49034	-0.61237
F	-2.18238	-0.55361	-0.58149
F	-3.27005	-1.82553	-2.16746
F	-4.40866	-0.15445	-1.03006
F	-4.88861	-2.38475	-0.60179
F	-3.78540	-1.11868	0.98852
F	-2.64676	-2.77663	-0.14043

NHC-Ag-TS1

N	3.17942	-0.73921	-1.72192
N	2.53284	-2.14633	-0.25895
N	1.03023	-1.95361	1.60017
N	-0.49012	-0.68568	2.46493
C	2.05659	-1.27037	-1.19550
C	1.66749	-2.85483	0.64921
H	0.86654	-3.34821	0.09346
H	2.25385	-3.60504	1.18487
C	-0.28786	-1.66832	1.58813
C	-1.77106	-0.01330	2.63350
H	-1.71588	0.95544	2.13406
H	-2.53329	-0.63606	2.15936
H	-1.97920	0.11444	3.69635
C	3.18438	0.37794	-2.65069
H	2.20837	0.43339	-3.12948
H	3.34819	1.30591	-2.09967

H	3.96157	0.22814	-3.40251
H	-1.10827	-2.17782	1.05483
Ag	-0.04314	-0.59073	-1.14394
O	-2.83034	-2.29405	0.59982
C	-3.05856	-1.67904	-0.47384
H	-1.77716	-0.69177	-0.86548
H	-2.89839	-2.18788	-1.44512
C	-4.09361	-0.60423	-0.54149
C	-4.88431	-0.33270	0.57643
C	-4.33449	0.08612	-1.73247
C	-5.88893	0.62880	0.51071
H	-4.71368	-0.91056	1.48166
C	-5.33569	1.04481	-1.80085
H	-3.70851	-0.12320	-2.59857
C	-6.11411	1.32185	-0.67560
H	-6.50408	0.83197	1.38354
H	-5.51045	1.58348	-2.72820
H	-6.89675	2.07377	-0.72760
C	1.67216	-1.09306	2.47047
C	0.71540	-0.29459	3.00597
C	3.91847	-2.12150	-0.18020
C	4.32151	-1.22894	-1.11534
H	0.79804	0.56007	3.65606
H	2.74461	-1.07018	2.56747
H	4.47767	-2.75249	0.49313
H	5.30941	-0.90671	-1.40330
P	1.51013	2.25410	0.39046
F	1.83459	2.52800	1.95890
F	0.13495	1.43404	0.83088
F	0.66693	3.61961	0.27466
F	2.87194	2.98736	-0.08884
F	1.15156	1.88198	-1.18355
F	2.34280	0.82796	0.49737

NHC-Ag-IM

Ag	3.61933	0.01674	-0.65480
N	2.03714	-2.35574	0.83799
N	0.63442	-1.05918	-0.10979
N	0.00703	1.29044	-0.25835
N	0.60216	3.11735	0.74056
C	1.97931	-1.25916	0.05072
C	0.09592	-0.01561	-0.94163
H	0.75710	0.11197	-1.80235
H	-0.91666	-0.26662	-1.25980
C	1.02887	1.91972	0.33409
C	1.41064	4.09922	1.44364
H	1.34906	5.05871	0.92909
H	1.05467	4.21240	2.46900
H	2.44771	3.76695	1.45293
C	3.27587	-2.96219	1.28270
H	4.10590	-2.37177	0.89148
H	3.32498	-2.97372	2.37391
H	3.35480	-3.98596	0.91001
C	0.77433	-2.84021	1.14549
C	-0.12250	-2.02092	0.54660
C	-1.10401	2.10869	-0.23523
C	-0.72845	3.25507	0.39306
H	0.62732	-3.72186	1.74861
H	-1.20243	-2.02524	0.50779
H	-2.05676	1.78337	-0.62689
H	-1.29126	4.14005	0.63935
H	2.04122	1.53509	0.42052
P	-3.68191	-0.41542	-0.12623
F	-2.27852	-0.12207	0.72902
F	-2.83983	0.01092	-1.51014
F	-3.15805	-1.95313	-0.33539
F	-4.42950	-0.80767	1.24161
F	-4.06671	1.16175	0.05472
F	-4.99131	-0.69029	-1.01143
H	4.77443	1.12763	-1.08952

NHC-Ag-TS2

N	1.47911	-2.50873	-2.04927
N	1.53501	-1.28910	-0.30150
N	0.59786	0.83133	0.30492
N	-0.78503	2.38859	-0.27612
C	0.67279	-1.81173	-1.22221
C	1.08420	-0.46047	0.79566
H	0.24665	-0.94315	1.30855
H	1.91476	-0.29362	1.47738
C	-0.68756	1.21994	0.36273
C	-2.03489	3.10467	-0.47034
H	-1.86679	4.17382	-0.34081
H	-2.43054	2.91019	-1.47055
H	-2.76107	2.75188	0.26295
C	1.01387	-3.24922	-3.20314

H	1.24380	-4.31175	-3.09430
H	-0.06592	-3.12149	-3.28234
H	1.48843	-2.87329	-4.11233
H	-1.52480	0.75104	0.89360
Ag	-1.45108	-1.36223	-1.09147
C	-3.96391	-0.36730	0.60033
H	-3.05051	-0.70664	-0.67932
H	-4.37620	-1.38237	0.49633
C	-4.83586	0.69883	0.04704
C	-5.07776	1.86426	0.78379
C	-5.44941	0.54974	-1.20136
C	-5.89696	2.86675	0.27177
H	-4.61812	1.95469	1.76434
C	-6.26696	1.55020	-1.71282
H	-5.25572	-0.35193	-1.77919
C	-6.48922	2.71607	-0.97977
H	-6.08249	3.76381	0.85680
H	-6.73034	1.42428	-2.68748
H	-7.12868	3.49788	-1.37992
C	1.33261	1.76405	-0.40090
C	0.45796	2.73939	-0.76721
C	2.84939	-1.64695	-0.55168
C	2.80811	-2.42777	-1.65838
H	0.61552	3.65265	-1.31686
H	2.39508	1.65016	-0.57145
H	3.68004	-1.27812	0.03264
H	3.60090	-2.90954	-2.20719
N	-3.19069	-0.05314	1.63466
C	-2.38605	-1.03379	2.17745
C	-1.57614	-0.66366	3.27733
C	-2.20774	-2.33400	1.64569
C	-0.64704	-1.53655	3.81725
H	-1.70949	0.33624	3.68401
C	-1.25406	-3.20004	2.18660
H	-2.84232	-2.68445	0.83588
C	-0.46788	-2.81123	3.26516
H	-0.04421	-1.22355	4.66562
H	-1.13397	-4.19171	1.75685
H	0.26980	-3.49086	3.68068
P	4.98160	1.26718	0.21955
F	5.55298	-0.26662	0.27043
F	5.72643	1.62866	1.59952
F	6.23000	1.75340	-0.66929
F	4.14183	0.86504	-1.15679
F	3.65090	0.73732	1.09425
F	4.28940	2.74256	0.15577

C1

N	-2.96393	-0.75909	0.75539
N	-2.96365	0.75917	-0.75544
C	-2.12927	-0.00004	0.00007
C	-2.52576	1.73948	-1.73153
H	-2.89251	2.73269	-1.46526
H	-1.43508	1.75281	-1.74253
C	-2.52636	-1.73953	1.73150
H	-2.89106	-1.47622	2.72627
H	-2.89282	-2.73274	1.46485
C	-4.28632	0.48122	-0.47891
C	-4.28649	-0.48092	0.47858
H	-5.09956	0.98305	-0.97843
H	-5.09992	-0.98261	0.97794
H	-2.88992	1.47593	-2.72644
H	-1.43569	-1.75267	1.74303
Ag	0.00001	-0.00007	-0.00001
C	4.28647	-0.47899	-0.48058
C	4.28634	0.47929	0.48076
H	5.09990	-0.97876	-0.98186
H	5.09963	0.97920	0.98211
N	2.96390	-0.75601	-0.75849
N	2.96370	0.75607	0.75854
C	2.12927	-0.00004	-0.00002
C	2.52581	1.73227	1.73875
H	2.89350	2.72640	1.47723
H	1.43514	1.74631	1.74904
H	2.88903	1.46396	2.73272
C	2.52627	-1.73243	-1.73858
H	1.43560	-1.74503	-1.75059
H	2.89141	-1.46536	-2.73218
H	2.89224	-2.72685	-1.47574

C1-RC

N	2.03329	-1.51977	-0.43615
N	2.80800	0.02477	0.87733
C	1.75958	-0.33528	0.12576
C	2.92049	1.25941	1.66720
H	3.35519	2.02970	1.06504

H	1.94765	1.56579	1.99063
C	1.14870	-2.27296	-1.33676
H	0.53576	-1.59050	-1.88764
H	1.73976	-2.85019	-2.01673
H	0.52668	-2.92643	-0.76147
Ag	-0.01775	0.77294	-0.12926
O	-1.70255	1.82321	-0.37094
C	-2.52591	1.18483	-1.35045
H	-3.42729	1.74671	-1.47965
H	-1.99999	1.13534	-2.28097
C	-2.87220	-0.24012	-0.88012
C	-2.96710	-0.52237	0.48928
C	-3.09242	-1.25459	-1.82155
C	-3.28223	-1.81908	0.91725
H	-2.79897	0.25220	1.20808
C	-3.40755	-2.55130	-1.39358
H	-3.01995	-1.03909	-2.86711
C	-3.50245	-2.83355	-0.02418
H	-3.35468	-2.03458	1.96282
H	-3.57570	-3.32587	-2.11239
H	-3.74304	-3.82362	0.30258
C	3.73455	-0.93473	0.78388
C	3.25153	-1.89770	-0.03505
H	4.68780	-0.94016	1.26987
H	3.75470	-2.80045	-0.31214
H	3.54053	1.08141	2.52088

C1-TS1

N	-3.77016	-0.36143	-0.00315
N	-2.85469	1.58105	-0.08344
C	-2.56604	0.25819	-0.04198
C	-1.87266	2.65012	-0.13179
H	-1.97029	3.29237	0.74529
H	-0.87514	2.20888	-0.14000
C	-3.96402	-1.80015	0.05230
H	-2.98802	-2.28714	0.03485
H	-4.48277	-2.07738	0.97156
H	-4.54407	-2.13719	-0.80826
Ag	-0.66625	-0.65266	-0.01746
O	1.29602	-1.61660	-0.06224
C	1.99517	-0.88151	0.70571
H	0.90725	0.47223	0.73714
H	1.82534	-0.91951	1.80090
C	3.23846	-0.24965	0.29767
C	3.69266	-0.34501	-1.02833
C	3.99780	0.43796	1.25941
C	4.89976	0.23497	-1.37907
H	3.09312	-0.89086	-1.75050
C	5.20264	1.02210	0.90025
H	3.63506	0.50118	2.28335
C	5.65151	0.91908	-0.41732
H	5.26689	0.15732	-2.39726
H	5.79638	1.55068	1.63847
H	6.59697	1.37279	-0.69929
C	-4.21884	1.78592	-0.07840
C	-4.79736	0.55851	-0.02770
H	-4.65303	2.77228	-0.11236
H	-5.83465	0.26472	-0.00822
H	-2.00823	3.24675	-1.03539

C1-IM

Ag	-0.00007	1.71667	-0.00004
N	-3.09207	1.25419	-0.54615
N	-2.80925	2.74860	0.95771
C	-2.82428	0.17433	-1.47321
H	-1.79547	0.20241	-1.76589
H	-3.44432	0.28485	-2.33822
C	-2.12308	1.88369	0.17553
C	-2.20226	3.67864	1.89779
H	-2.43664	4.70644	1.61621
H	-2.56998	3.48728	2.90704
H	-1.12108	3.53779	1.87670
C	-4.35393	1.71409	-0.21240
C	-4.17084	2.66266	0.73864
H	-5.25474	1.34913	-0.68016
H	-4.88044	3.28311	1.26295
H	-3.03518	-0.76213	-1.00049
H	1.62935	1.58848	-0.13478

C1-TS2

N	-3.35399	-2.20969	0.05809
N	-3.29299	-0.21787	0.83588
C	-2.52997	-1.13755	0.19209
C	-2.82671	1.11064	1.19519
H	-1.78120	1.21224	0.89294

H	-3.40716	1.87509	0.67502
C	-2.96127	-3.44930	-0.57900
H	-1.92155	-3.35130	-0.89490
H	-3.04637	-4.28494	0.11976
H	-3.58509	-3.64795	-1.45387
Ag	-0.45857	-0.83305	-0.40759
C	2.30282	0.63328	-0.50654
H	1.26403	-0.50718	-0.65761
H	2.25712	0.76813	-1.60213
C	3.49831	-0.12714	-0.07190
C	3.97476	-0.03307	1.23920
C	4.17197	-0.94933	-0.98037
C	5.10268	-0.74570	1.62887
H	3.44561	0.62063	1.92621
C	5.29963	-1.66349	-0.59145
H	3.79791	-1.02628	-2.00045
C	5.76859	-1.56412	0.71711
H	5.46947	-0.66126	2.64888
H	5.81549	-2.29666	-1.30874
H	6.65085	-2.11978	1.02400
C	-4.55998	-0.70070	1.09487
C	-4.60020	-1.96528	0.60209
H	-5.31095	-0.11372	1.59965
H	-5.39192	-2.69764	0.59222
N	1.82314	1.56910	0.30947
C	0.71353	2.25439	-0.08956
C	0.21392	3.26290	0.77465
C	-0.04085	1.99004	-1.26792
C	-0.93126	3.97678	0.47335
H	0.77764	3.46010	1.68294
C	-1.20160	2.71237	-1.55517
H	0.32176	1.26741	-1.99366
C	-1.65444	3.70823	-0.69835
H	-1.27260	4.75476	1.15303
H	-1.74630	2.49644	-2.47241
H	-2.55045	4.27574	-0.93563
H	-2.90628	1.26062	2.27424

C2

N	-2.67002	-1.91929	1.45236
N	-3.04461	-0.15987	0.29361
C	-2.04925	-1.03640	0.63869
C	-2.00784	-3.05022	2.07479
H	-0.96861	-3.05681	1.74432
H	-2.04073	-2.95938	3.16250
H	-2.49027	-3.98343	1.77712
Ag	0.00004	-0.85026	-0.00004
C	-4.25081	-0.50506	0.88688
C	-4.00988	-1.61638	1.62113
H	-5.15382	0.06997	0.76854
H	-4.66443	-2.20409	2.24499
C	-2.84175	0.95077	-0.57416
C	-3.92372	1.60188	-1.16389
C	-1.32963	2.33455	-1.57318
C	-3.65123	2.68692	-1.98806
H	-4.94342	1.26763	-1.00896
C	-2.33014	3.06928	-2.19923
H	-0.27882	2.58332	-1.70953
H	-4.46659	3.22006	-2.46651
H	-2.08080	3.91003	-2.83668
N	-1.57265	1.29585	-0.76922
C	4.25091	-0.50490	-0.88668
C	4.01012	-1.61624	-1.62093
H	5.15388	0.07018	-0.76832
H	4.66481	-2.20398	-2.24463
C	2.84164	0.95091	0.57415
C	3.92354	1.60245	1.16355
C	3.65089	2.68747	1.98769
H	4.94332	1.26858	1.00838
C	1.32931	2.33429	1.57346
C	2.32973	3.06942	2.19917
H	4.46620	3.22093	2.46587
H	0.27844	2.58272	1.71002
H	2.08029	3.91016	2.83660
C	2.00826	-3.05026	-2.07473
H	0.96879	-3.05645	-1.74500
H	2.04198	-2.95992	-3.16246
H	2.49023	-3.98346	-1.77628
C	2.04933	-1.03632	-0.63880
N	2.67026	-1.91919	-1.45234
N	3.04464	-0.15975	-0.29356
N	1.57248	1.29559	0.76955

C2-RC

N	-2.77568	-2.36899	-0.08768
N	-3.25093	-0.28107	-0.05776

C	-2.18594	-1.14864	-0.04912
C	-2.03360	-3.61365	-0.09296
H	-0.97100	-3.36764	-0.05421
H	-2.29831	-4.22062	0.77604
H	-2.24096	-4.17824	-1.00510
Ag	-0.14673	-0.62609	-0.03866
O	1.80572	-0.04590	-0.04813
C	2.83347	-0.88416	0.30171
H	2.77037	-1.24754	1.35421
H	2.86745	-1.82384	-0.29830
C	4.19127	-0.22786	0.15088
C	4.29952	1.07594	-0.33023
C	5.35982	-0.91723	0.48845
C	5.54811	1.67852	-0.47172
H	3.37938	1.59280	-0.58570
C	6.60787	-0.32008	0.34889
H	5.28513	-1.93726	0.86658
C	6.70702	0.98504	-0.13353
H	5.61808	2.69690	-0.84881
H	7.50687	-0.87097	0.61665
H	7.68126	1.45496	-0.24367
C	-4.46059	-0.96132	-0.09743
C	-4.15622	-2.27987	-0.12026
H	-5.41358	-0.46112	-0.14682
H	-4.79308	-3.14907	-0.16678
C	-3.11049	1.13193	0.00509
C	-4.16490	1.92655	0.45573
C	-1.74952	2.93016	-0.31225
C	-3.96480	3.30058	0.49289
H	-5.09884	1.48990	0.79351
C	-2.73471	3.82065	0.10202
H	-0.76586	3.28099	-0.61623
H	-4.75985	3.95414	0.83895
H	-2.53978	4.88725	0.12384
N	-1.92976	1.60909	-0.37330

C2-TS1

N	-0.93991	2.44518	0.43865
N	-2.37118	0.85222	0.52590
C	-1.13082	1.16717	0.04249
C	0.25957	3.21793	0.14178
H	1.00155	2.57993	-0.36175
H	0.00542	4.05891	-0.50749
H	0.68263	3.60185	1.07319
Ag	0.31446	-0.01072	-0.99842
O	2.52736	1.60547	-1.44083
C	2.84649	0.36879	-1.50758
H	1.83706	-0.47168	-1.91226
H	3.48062	0.02588	-2.36523
C	3.28971	-0.34300	-0.23448
C	3.24202	0.34298	0.97851
C	3.76845	-1.65488	-0.25406
C	3.65055	-0.27663	2.15764
H	2.89143	1.37288	0.95723
C	4.18097	-2.27572	0.91896
H	3.81001	-2.18869	-1.20373
C	4.11942	-1.58774	2.13204
H	3.61340	0.26678	3.09928
H	4.55401	-3.29695	0.89259
H	4.44351	-2.07129	3.05002
C	-2.93585	1.92531	1.20205
C	-2.02529	2.92689	1.14828
H	-3.89446	1.86954	1.69133
H	-2.05107	3.92451	1.55743
C	-2.99443	-0.40982	0.32882
C	-4.38594	-0.50928	0.34179
C	-2.73294	-2.63460	-0.06318
C	-4.94466	-1.76692	0.15444
H	-5.01088	0.36917	0.46495
C	-4.10613	-2.85763	-0.05238
H	-2.03611	-3.45375	-0.22825
H	-6.02379	-1.88707	0.15597
H	-4.50396	-3.85483	-0.20606
N	-2.17525	-1.43774	0.13325

C2-IM

N	1.77413	1.67149	0.04555
N	-0.32487	1.28727	0.09196
C	0.88686	0.64765	0.02097
C	3.20903	1.47393	-0.01627
H	3.39164	0.40086	-0.10324
H	3.62851	1.98691	-0.88500
H	3.68707	1.85139	0.89091
Ag	1.28787	-1.51579	-0.08717
C	-0.17854	2.66675	0.15284
C	1.15379	2.90673	0.12862

H	-1.01263	3.34270	0.24846
H	1.70375	3.83369	0.17317
C	-1.56775	0.59877	0.06497
C	-2.71906	1.24409	-0.38695
C	-2.67818	-1.34885	0.44824
C	-3.90411	0.51932	-0.38938
H	-2.68644	2.26549	-0.75202
C	-3.89096	-0.80559	0.03503
H	-2.61049	-2.38316	0.77887
H	-4.82299	0.98339	-0.73509
H	-4.79459	-1.40549	0.04079
N	-1.53421	-0.66292	0.47826
H	1.71446	-3.12147	-0.21875

C2-TS2

N	-3.35484	0.11525	0.47742
N	-2.31972	1.91047	1.00412
C	-2.10151	0.66814	0.52034
C	-1.25996	2.88063	1.22567
Ag	-0.20417	-0.20496	-0.14377
C	2.92772	-0.08056	-0.54931
H	1.47269	-0.68269	-0.43392
H	2.80415	-0.11029	-1.64627
C	3.72908	-1.21203	-0.02808
C	4.35098	-1.13827	1.22217
C	3.88197	-2.37312	-0.79207
C	5.10933	-2.20332	1.69373
H	4.23134	-0.22339	1.79482
C	4.63983	-3.43944	-0.32128
H	3.39370	-2.43281	-1.76393
C	5.25639	-3.35799	0.92594
H	5.59346	-2.13355	2.66482
H	4.75270	-4.33471	-0.92752
H	5.85089	-4.18920	1.29618
C	-3.66052	2.14144	1.25904
C	-4.32025	1.00477	0.93167
H	-4.02567	3.08374	1.63629
H	-5.37335	0.77624	0.94282
N	2.99010	1.08085	0.09319
C	2.24524	2.11892	-0.38463
C	2.34452	3.36513	0.28567
C	1.31151	2.04110	-1.45559
C	1.59655	4.46177	-0.10225
H	3.04569	3.42679	1.11414
C	0.55326	3.15256	-1.82980
H	1.22616	1.12634	-2.03558
C	0.68936	4.36735	-1.16779
H	1.71526	5.40597	0.42528
H	-0.14189	3.06312	-2.66269
H	0.10757	5.23242	-1.47555
H	-0.31123	2.43913	0.90957
H	-1.43502	3.77748	0.62860
H	-1.20652	3.14662	2.28385
C	-3.60123	-1.21509	0.04595
C	-4.75657	-1.88333	0.45224
C	-4.94728	-3.18071	-0.00517
H	-5.46897	-1.41785	1.12512
C	-2.86263	-3.00515	-1.15054
H	-5.83336	-3.73469	0.28974
H	-2.07593	-3.41602	-1.77982
H	-4.09494	-4.77283	-1.19826
C	-3.98449	-3.76059	-0.82501
N	-2.66960	-1.75082	-0.73702

C3

Ag	-0.16525	-0.49024	-0.28712
N	1.22362	-3.36525	-0.64418
N	2.46264	-2.08176	0.53242
N	4.11976	-0.35362	0.94209
N	5.35021	1.14033	-0.03789
N	-5.66474	-0.25775	0.16772
N	-4.02649	0.74865	1.16205
N	-2.01592	1.94902	0.51433
N	-0.76549	2.58624	-1.09209
C	1.27197	-2.11209	-0.14006
C	2.85690	-0.95969	1.34554
H	2.08885	-0.17261	1.26564
H	2.97045	-1.26433	2.38848
C	4.24955	0.38623	-0.18416
C	-4.50888	0.39283	-0.04876
C	-2.63332	1.10020	1.48333
H	-2.64963	1.58005	2.46534
H	-2.07633	0.15362	1.51498
C	-1.04757	1.49970	-0.34367
C	5.70006	2.22435	-0.93971
H	4.88279	2.95383	-0.92504

H	6.61772	2.69150	-0.58730
H	5.86088	1.84792	-1.95079
C	0.30506	2.62734	-2.07358
H	0.40578	1.63360	-2.51370
H	0.05613	3.34740	-2.85456
H	1.23755	2.88881	-1.55947
C	-6.46098	-0.90149	-0.86593
H	-6.81291	-0.17089	-1.59579
H	-5.86167	-1.67039	-1.35808
H	-7.32367	-1.36775	-0.39428
C	0.11378	-3.86934	-1.44102
H	-0.78418	-3.29495	-1.18999
H	0.33942	-3.78128	-2.50658
H	-0.05631	-4.91822	-1.19578
C	2.34850	-4.09782	-0.31012
C	3.13484	-3.29038	0.44386
C	5.09100	0.03860	1.84926
C	5.86052	0.96649	1.23536
C	-5.88827	-0.37259	1.52411
C	-4.86104	0.25197	2.14564
C	-2.31008	3.28942	0.30899
C	-1.50854	3.68774	-0.70896
H	2.48452	-5.12018	-0.62598
H	4.08585	-3.46686	0.92129
H	5.14234	-0.38268	2.83958
H	6.72545	1.50994	1.57694
H	-6.75672	-0.87526	1.91559
H	-4.64199	0.38554	3.19208
H	-3.03810	3.82630	0.89648
H	-1.39300	4.65173	-1.17849
C	-3.95997	0.70856	-1.38094
H	-3.74625	1.77637	-1.48097
H	-3.04071	0.13417	-1.53405
H	-4.67348	0.42448	-2.15439
C	3.46937	0.23322	-1.42465
H	3.84732	-0.60648	-2.02266
H	2.41639	0.06349	-1.18627
H	3.52323	1.14892	-2.01333
Cl	2.32199	2.29005	0.62944
Cl	-2.56820	-1.95269	0.05807

C3-RC

N	2.72685	0.07292	0.68112
N	1.13950	0.99310	-0.47184
N	-1.29801	0.87632	-0.53446
N	-3.04973	-0.08434	0.28512
C	1.39275	0.19505	0.59540
C	-0.07588	1.02863	-1.28963
H	-0.09358	1.98373	-1.81879
H	-0.01063	0.16715	-1.97225
C	-1.99262	-0.27750	-0.50405
C	-4.06836	-1.08900	0.57528
H	-3.76116	-2.03644	0.13668
H	-4.16985	-1.20457	1.65447
H	-5.02279	-0.78096	0.14703
C	3.42347	-0.75224	1.66005
H	3.26920	-0.36848	2.66998
H	3.06729	-1.78134	1.58719
H	4.48726	-0.72775	1.43385
C	3.32944	0.74702	-0.36111
C	2.34084	1.31645	-1.08637
C	-1.93711	1.82212	0.24668
C	-3.03868	1.21559	0.76270
H	4.39963	0.76564	-0.48334
H	2.37410	1.90573	-1.98802
H	-1.56426	2.82816	0.35445
H	-3.81405	1.59155	1.41078
C	0.45506	-0.37256	1.58456
H	0.98220	-0.58397	2.51592
H	-0.35740	0.31861	1.81576
H	0.05271	-1.31350	1.18573
H	-1.59171	-1.20760	-0.95140
Cl	0.36172	-2.09196	-1.20699

C3-TS1

N	-1.52101	-2.80842	-0.63230
N	-2.11231	-1.16643	0.59672
C	-1.08426	-1.61230	-0.17686
C	-2.11133	0.06988	1.36447
H	-3.12567	0.18038	1.79478
H	-1.32962	0.04401	2.12515
C	-0.76787	-3.66574	-1.52333
H	-1.30603	-3.80710	-2.46356
H	0.19335	-3.19162	-1.72880
H	-0.59477	-4.63986	-1.06007
Ag	0.62909	-0.50229	-0.84385

O	2.79496	2.49646	-0.56674
C	3.01954	1.31686	-0.98286
H	1.86021	0.68940	-1.39534
H	3.47083	1.18401	-1.99457
C	3.56686	0.29089	-0.00258
C	3.85352	0.67885	1.30393
C	3.80822	-1.03106	-0.38950
C	4.35495	-0.24573	2.21958
H	3.68358	1.71856	1.57312
C	4.31235	-1.95456	0.51850
H	3.59237	-1.32967	-1.41727
C	4.58230	-1.56280	1.83153
H	4.57611	0.06471	3.23771
H	4.49988	-2.97906	0.20719
H	4.97522	-2.28280	2.54404
C	-3.17256	-2.05815	0.61986
C	-2.78995	-3.10199	-0.15683
H	-4.10349	-1.78627	1.11213
H	-3.30598	-4.00986	-0.42442
N	-1.88385	1.22110	0.50812
C	-2.80709	1.65958	-0.42364
C	-0.75431	1.96113	0.43279
C	-2.21647	2.69345	-1.07641
H	-3.80529	1.21605	-0.42569
H	-2.57773	3.33035	-1.86669
N	-0.95267	2.86657	-0.53965
C	0.02057	3.86675	-0.96531
H	-0.25620	4.20103	-1.96433
H	-0.00015	4.72227	-0.28570
H	1.02977	3.43341	-0.98489
C	0.44111	1.87609	1.29036
H	0.35637	2.55972	2.14359
H	0.57467	0.86597	1.68587
H	1.35108	2.15491	0.72672
Cl	-5.29000	0.31887	1.18960

C3-IM

N	-2.13733	1.88264	0.37618
N	-0.45319	0.93263	-0.50979
C	-1.74744	0.69424	-0.13964
C	0.38790	-0.08261	-1.07535
H	-0.25750	-0.86643	-1.48479
H	1.02487	0.36616	-1.84331
C	-3.46881	2.13096	0.89081
H	-3.42017	2.46690	1.92925
H	-3.97595	2.89167	0.29251
H	-4.03212	1.19804	0.83921
Ag	-2.74185	-1.25529	-0.18496
C	-0.03790	2.22566	-0.21765
C	-1.11801	2.82571	0.33958
H	0.99013	2.53483	-0.40569
H	-1.25373	3.83150	0.70461
N	1.28966	-0.63630	-0.06079
C	1.06806	-0.58725	1.30442
C	2.59747	-0.87549	-0.29147
C	2.25130	-0.85542	1.90440
H	0.09180	-0.37521	1.70875
H	2.51718	-0.92391	2.94584
N	3.18027	-1.07627	0.90415
C	4.62120	-1.07472	1.07781
H	4.84795	-1.20070	2.13527
H	5.07708	-1.89331	0.51827
H	4.99990	-0.10858	0.72160
C	3.23268	-1.15643	-1.59245
H	2.74462	-0.60484	-2.39407
H	4.26856	-0.81867	-1.57965
H	3.19306	-2.23044	-1.81656
Cl	3.27014	1.78337	-0.68079
H	-3.41570	-2.77267	-0.17584

C3-TS2

N	-3.21640	1.25864	-1.50578
N	-3.06989	-0.09739	0.13075
N	-1.54008	-1.81331	0.79215
N	0.35493	-2.73619	0.32280
C	-2.30671	0.67753	-0.68963
C	-2.56115	-0.88098	1.24264
H	-2.12585	-0.22911	2.00167
H	-3.40483	-1.48537	1.62811
C	-0.20800	-1.71164	0.98662
C	1.78692	-2.94346	0.18719
H	2.21123	-2.20775	-0.50384
H	2.27952	-2.84062	1.15478
H	1.95994	-3.94848	-0.19497
C	-2.87720	2.19325	-2.55743
H	-3.31645	3.17367	-2.35588

H	-1.79027	2.29029	-2.59350
H	-3.23779	1.83137	-3.52323
Ag	-0.13133	0.76236	-0.89428
C	2.99640	1.01609	-0.29249
H	1.65891	0.47607	-0.96584
H	3.08237	1.70277	-1.14981
C	3.89769	-0.15653	-0.38551
C	4.39775	-0.77319	0.76757
C	4.23595	-0.68745	-1.63618
C	5.20828	-1.90009	0.66916
H	4.14056	-0.33802	1.72957
C	5.04009	-1.81757	-1.73468
H	3.84350	-0.21272	-2.53397
C	5.52585	-2.43166	-0.58028
H	5.60049	-2.36400	1.57088
H	5.29053	-2.22094	-2.71216
H	6.15789	-3.31226	-0.65562
C	-1.82635	-2.89981	-0.01421
C	-0.63142	-3.47242	-0.30998
C	-4.42052	-0.01395	-0.17000
C	-4.50956	0.85542	-1.20645
H	-0.38844	-4.34351	-0.89547
H	-2.87397	-3.16208	-0.18766
H	-5.13519	-0.65881	0.33335
H	-5.36384	1.20582	-1.76298
N	2.67811	1.45144	0.92315
C	1.75567	2.45869	1.01515
C	1.27841	2.79007	2.30665
C	1.14153	3.11208	-0.08791
C	0.25699	3.70378	2.48913
H	1.74197	2.28665	3.15222
C	0.11528	4.04151	0.11143
H	1.52542	2.95818	-1.09307
C	-0.33994	4.33531	1.38922
H	-0.08633	3.93268	3.49495
H	-0.32422	4.53997	-0.75066
H	-1.14077	5.05371	1.53703
C	0.51174	-0.74623	1.83893
H	0.72592	-1.17726	2.82474
H	1.46426	-0.42211	1.39931
H	-0.07448	0.16344	1.98987
Cl	-4.91180	-3.02689	0.96577

NHC-Ag-RC-*p*-OCH$_3$

N	4.12111	-0.51372	-0.37544
N	2.87559	0.85306	0.69926
N	0.72341	1.96969	0.49563
N	-1.06158	2.13689	-0.70670
C	2.87598	-0.39373	0.13036
C	1.73147	1.40859	1.38977
H	1.24686	0.61838	1.96721
H	2.07443	2.19099	2.06921
C	-0.42896	1.35008	0.16501
C	-2.34953	1.78945	-1.30491
H	-3.16254	2.27312	-0.76044
H	-2.35522	2.10629	-2.34716
H	-2.47006	0.70564	-1.23803
C	4.60616	-1.69167	-1.07663
H	3.79663	-2.41861	-1.12725
H	4.91755	-1.42578	-2.08813
H	5.45017	-2.12803	-0.53986
H	-0.80387	0.31961	0.43528
Ag	1.03511	-1.44442	0.02051
O	-1.04472	-1.26404	0.06190
C	-2.12963	-2.11137	0.31854
H	-2.20399	-2.92033	-0.42768
H	-2.04441	-2.60316	1.30477
C	-3.38080	-1.27288	0.27763
C	-3.62932	-0.35397	1.30466
C	-4.24022	-1.29226	-0.82310
C	-4.70632	0.52367	1.23408
H	-2.96927	-0.34079	2.17125
C	-5.32140	-0.41435	-0.90049
H	-4.05791	-2.00474	-1.62541
C	-5.55436	0.49813	0.12555
H	-4.89740	1.21855	2.04779
C	0.81172	3.17177	-0.18490
C	-0.31587	3.27587	-0.93690
C	4.10063	1.48396	0.54535
C	4.88707	0.61399	-0.13340
H	-0.64970	4.05368	-1.60446
H	1.65207	3.83823	-0.07370
H	4.30883	2.46498	0.94334
H	5.91457	0.69236	-0.45122
H	-5.97623	-0.44777	-1.74606
O	-6.67044	1.38931	0.05433
C	-7.86958	0.67911	0.37458
H	-8.67511	1.37366	0.49127

H	-8.10034	-0.00537	-0.41481
H	-7.73055	0.13761	1.28692

NHC-Ag-TS1-*p*-OCH$_3$

N	3.93760	-0.76364	-0.34659
N	2.82233	0.62349	0.83542
N	0.84850	1.93514	0.47429
N	-0.86123	2.23697	-0.80935
C	2.67744	-0.47863	0.04212
C	1.70094	1.28660	1.46746
H	1.06995	0.55800	1.99091
H	2.08043	2.02657	2.17426
C	-0.41212	1.53055	0.23167
C	-2.16727	2.05529	-1.43733
H	-2.08138	1.35132	-2.26770
H	-2.86574	1.65567	-0.70045
H	-2.52439	3.01732	-1.80245
C	4.29486	-1.88617	-1.19938
H	3.37898	-2.39121	-1.50416
H	4.82453	-1.53149	-2.08499
H	4.92916	-2.58694	-0.65385
H	-0.92936	0.77463	0.88457
Ag	0.72458	-1.29772	-0.31251
O	-1.07903	-0.57077	1.88869
C	-1.71359	-1.36836	1.11457
H	-1.03868	-1.54692	-0.21010
H	-1.65219	-2.45500	1.31420
C	-3.05061	-0.93576	0.57855
C	-3.68620	0.15102	1.18670
C	-3.68437	-1.59793	-0.47653
C	-4.93709	0.57537	0.73895
H	-3.19365	0.63098	2.02880
C	-4.92556	-1.16925	-0.92936
H	-3.18521	-2.43962	-0.95363
C	-5.55381	-0.07825	-0.32454
H	-5.43419	1.40887	1.22759
H	-5.40925	-1.68442	-1.75418
C	1.21325	2.89928	-0.44436
C	0.13457	3.08817	-1.25036
C	4.14828	1.01324	0.93268
C	4.85303	0.12791	0.18558
H	-0.01400	3.75302	-2.08595
H	2.18792	3.36016	-0.44593
H	4.46877	1.85402	1.52803
H	5.91158	0.05106	-0.00524
O	-6.83557	0.35494	-0.78751
C	-6.66657	1.27312	-1.87069
H	-6.18698	0.77617	-2.68797
H	-7.62358	1.63414	-2.18484
H	-6.06269	2.09606	-1.54976

NHC-Ag-RC-*m*-CF$_3$

N	4.12111	-0.51372	-0.37544
N	2.87559	0.85306	0.69926
N	0.72341	1.96969	0.49563
N	-1.06158	2.13689	-0.70670
C	2.87598	-0.39373	0.13036
C	1.73147	1.40859	1.38977
H	1.24686	0.61838	1.96721
H	2.07443	2.19099	2.06921
C	-0.42896	1.35008	0.16501
C	-2.34953	1.78945	-1.30491
H	-3.16254	2.27312	-0.76044
H	-2.35522	2.10629	-2.34716
H	-2.47006	0.70564	-1.23803
C	4.60616	-1.69167	-1.07663
H	3.79663	-2.41861	-1.12725
H	4.91755	-1.42578	-2.08813
H	5.45017	-2.12803	-0.53986
H	-0.80387	0.31961	0.43528
Ag	1.03511	-1.44442	0.02051
O	-1.04472	-1.26404	0.06190
C	-2.12963	-2.11137	0.31854
H	-2.20399	-2.92033	-0.42768
H	-2.04441	-2.60316	1.30477
C	-3.38080	-1.27288	0.27763
C	-3.62932	-0.35397	1.30466
C	-4.24022	-1.29226	-0.82310
C	-4.70632	0.52367	1.23408
H	-2.96927	-0.34079	2.17125
C	-5.32140	-0.41435	-0.90049
H	-4.05791	-2.00474	-1.62541
C	-5.55436	0.49813	0.12555
C	0.81172	3.17177	-0.18490
C	-0.31587	3.27587	-0.93690
C	4.10063	1.48396	0.54535

C	4.88707	0.61399	-0.13340
H	-0.64970	4.05368	-1.60446
H	1.65207	3.83823	-0.07370
H	4.30883	2.46498	0.94334
H	5.91457	0.69236	-0.45122
H	-6.75629	1.45787	0.04885
H	-5.97623	-0.44777	-1.74606
C	-4.97704	1.50817	2.38694
F	-6.24427	1.35070	2.82494
F	-4.80667	2.77329	1.94769
F	-4.11751	1.26354	3.39880

NHC-Ag-TS1-*m*-CF_3

N	4.64487	-0.53270	-0.67575
N	3.50630	0.71970	0.67719
N	1.53022	2.12792	0.65197
N	-0.23749	2.81177	-0.42931
C	3.40241	-0.41429	-0.11264
C	2.39631	1.24746	1.46341
H	1.76006	0.42580	1.81853
H	2.79147	1.81452	2.30588
C	0.25043	1.80462	0.32693
C	-1.61144	2.87090	-0.96621
H	-1.98706	1.85944	-1.10573
H	-2.26319	3.40429	-0.27526
H	-1.60005	3.37874	-1.92786
C	5.03289	-1.61513	-1.58972
H	4.16618	-2.25452	-1.75045
H	5.35809	-1.20101	-2.54363
H	5.83868	-2.20378	-1.15214
H	-0.22408	0.86530	0.69234
Ag	1.64934	-1.61623	-0.18318
O	-0.19367	-0.56975	1.59357
C	-0.68910	-1.74674	1.21560
H	0.02592	-2.32682	0.06666
H	-0.48836	-2.60798	1.87116
C	-2.08128	-1.76662	0.62812
C	-2.68902	-2.95989	0.19734
C	-2.79197	-0.56501	0.53599
C	-3.98701	-2.94714	-0.32623
H	-2.14151	-3.89473	0.26762
C	-4.08510	-0.55454	-0.00506
H	-2.34820	0.33925	0.94033
C	-4.69214	-1.74046	-0.44152
H	-5.69524	-1.72128	-0.84949
C	1.85878	3.36546	0.08985
C	0.74654	3.79457	-0.58704
C	4.78995	1.28210	0.60002
C	5.50544	0.48926	-0.25258
H	0.57407	4.69270	-1.15015
H	2.82236	3.82496	0.20969
H	5.08292	2.15224	1.16046
H	6.52798	0.55514	-0.57626
H	-4.45052	-3.87042	-0.64836
C	-4.78363	0.74916	-0.16562
F	-6.16794	0.65579	-0.18507
F	-4.43668	1.68449	0.82858
F	-4.42415	1.39745	-1.39074

NHC-Ag-RC-*p*-CF_3

N	-5.03614	-0.03492	0.60048
N	-3.69824	1.07327	-0.64800
N	-1.42095	1.91391	-0.63427
N	0.42038	1.95569	0.49235
C	-3.83971	-0.13493	-0.01497
C	-2.53774	1.42221	-1.43772
H	-2.18981	0.53501	-1.97207
H	-2.81563	2.19216	-2.15977
C	-0.33966	1.18276	-0.28750
C	1.68376	1.52692	1.08804
H	2.52557	1.99781	0.57848
H	1.69133	1.80141	2.14289
H	1.75944	0.44287	0.98874
C	-5.63191	-1.09276	1.40100
H	-4.93918	-1.93263	1.43037
H	-5.81363	-0.73805	2.41685
H	-6.57398	-1.41704	0.95580
H	-0.10191	0.11042	-0.53623
Ag	-2.17937	-1.45017	-0.04159
O	-0.13932	-1.59338	-0.42180
C	0.82822	-2.59927	-0.46082
H	0.71389	-3.32970	0.35802
H	0.77489	-3.17781	-1.40156
C	2.17716	-1.93584	-0.34925
C	2.59974	-1.07178	-1.36938
C	2.96120	-2.04700	0.79983

C	3.75261	-0.31413	-1.22982
H	2.00206	-0.99425	-2.27526
C	4.12018	-1.28728	0.95255
H	2.65149	-2.72422	1.59267
C	4.50618	-0.40942	-0.05618
H	4.07844	0.35364	-2.02274
H	4.71986	-1.36781	1.85367
C	-1.33251	3.16880	-0.05835
C	-0.17162	3.19317	0.64653
C	-4.79251	1.89570	-0.42565
C	-5.63788	1.18913	0.36272
H	0.28378	3.97514	1.23240
H	-2.08887	3.92383	-0.20137
H	-4.88388	2.88034	-0.85723
H	-6.60817	1.44133	0.76010
C	5.65581	0.52620	0.13927
F	6.39350	0.67030	-0.97369
F	6.46924	0.14836	1.13611
F	5.19235	1.77044	0.45640

NHC-Ag-TS1-*p*-CF_3

N	3.93760	-0.76364	-0.34659
N	2.82233	0.62349	0.83542
N	0.84850	1.93514	0.47429
N	-0.86123	2.23697	-0.80935
C	2.67744	-0.47863	0.04212
C	1.70094	1.28660	1.46746
H	1.06995	0.55800	1.99091
H	2.08043	2.02657	2.17426
C	-0.41212	1.53055	0.23167
C	-2.16727	2.05529	-1.43733
H	-2.08138	1.35132	-2.26770
H	-2.86574	1.65567	-0.70045
H	-2.52439	3.01732	-1.80245
C	4.29486	-1.88617	-1.19938
H	3.37898	-2.39121	-1.50416
H	4.82453	-1.53149	-2.08499
H	4.92916	-2.58694	-0.65385
H	-0.92936	0.77463	0.88457
Ag	0.72458	-1.29772	-0.31251
O	-1.07903	-0.57077	1.88869
C	-1.71359	-1.36836	1.11457
H	-1.03868	-1.54692	-0.21010
H	-1.65219	-2.45500	1.31420
C	-3.05061	-0.93576	0.57855
C	-3.68620	0.15102	1.18670
C	-3.68437	-1.59793	-0.47653
C	-4.93709	0.57537	0.73895
H	-3.19365	0.63098	2.02880
C	-4.92556	-1.16925	-0.92936
H	-3.18521	-2.43962	-0.95363
C	-5.55381	-0.07825	-0.32454
C	1.21325	2.89928	-0.44436
C	0.13457	3.08817	-1.25036
C	4.14828	1.01324	0.93268
C	4.85303	0.12791	0.18558
H	-0.01400	3.75302	-2.08595
H	2.18792	3.36016	-0.44593
H	4.46877	1.85402	1.52803
H	5.91158	0.05106	-0.00524
H	-5.40207	-1.67676	-1.74193
H	-5.42661	1.39617	1.22014
C	-6.93417	0.38826	-0.82312
F	-7.08506	1.70449	-0.56376
F	-7.90083	-0.30902	-0.18920
F	-7.02666	0.17825	-2.15348

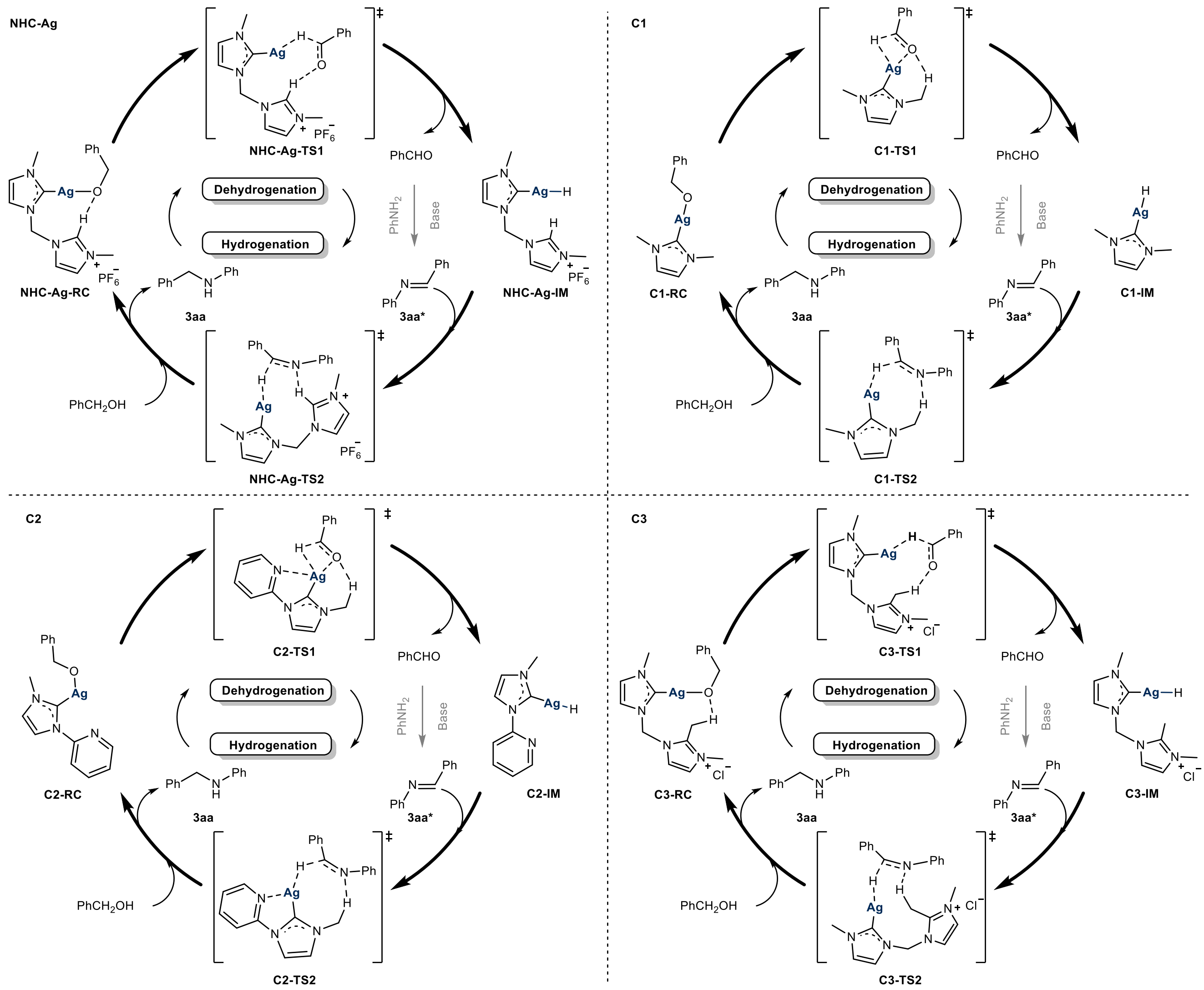

Figure S10. Reaction pathway of **NHC-Ag**, **C1**, **C2** and **C3**.

Table S2. Hydride dissociation energy (HDE) of Ag-H and orbital energy (OE) of Ag-H bond for ligands (**L1**, **L6**, **L7** and **L8**)

Ligand	^{1}H NMR shift (ppm)	Catalyst	HDE (ΔΔ*G* kcal/mmol)	OE (a.u.)
L1	3.8	**C1**	0.0	-0.163
L6	9.5	**NHC-Ag**	-38.2	-0.136
L8	4.0	**C2**	-5.8	-0.148
L7	2.8	**C3**	-32.9	-0.168

VI. References

1. M. Huang, Y. Li, J. Liu, S. Shu, Y. Liu and Z. Ke, Room temperature *N*-heterocyclic carbene manganese catalyzed selective *N*-alkylation of anilines with alcohols, *Chem. Commun.*, **2019,** 55, 6213-6216.
2. T. Yagyu, A. Tsubouchi and Y. Nishimura, Syntheses and Characterization of Palladium(II) Complexes with A Bi dentate Bis-NHC Ligand Having Methyl and Aryl Substituents on Terminal Nitrogen Atoms, *J. Organomet. Chem.*, **2014,** 767, 1-5.

3. G. Y. Kim, H. J. Jung, G. Park and D.-H. Lee, Synthesis and Characterization of Ag(I) and Pd(II) Complexes with a Pyridine Substituted N-Heterocyclic Carbene Ligand, *Bull. Korean Chem. Soc.*, **2010,** 31, 1739-1742.
4. K. R. Penn, E. J. Anders and V. N. G. Lindsay, Expedient Synthesis of Bis(imidazolium) Dichloride Salts and Bis (NHC) Complexes from Imidazoles Using DMSO as a Key Polar Additive, *Organometallics*, **2021,** 40, 3871-3875.
5. C. A. Quezada, J. C. Garrison, M. J. Panzner, C. A. Tessier and W. J. Youngs, The Potential Use of Rhodium N-Heterocyclic Carbene Complexes as Radiopharmaceuticals: The Transfer of a Carbene from Ag(I) to $RhCl_3 \cdot 3H_2O$, *Organometallics*, **2004,** 23, 4846-4848.
6. X.-B. Lan, Z. Ye, C. Yang, W. Li, J. Liu, M. Huang, Y. Liu and Z. Ke, Tungsten-Catalyzed Direct *N*-Alkylation of Anilines with Alcohols, *ChemSusChem*, **2021,** 14, 860-865.
7. L. Wang, X. Jv, R. Wang, L. Ma, J. Liu, J. Sun, T. Shi, L. Zhao, X. Zhang and B. Wang, Highly Selective Synergistic N Alkylation of Amines with ROH Catalyzed by Nickel−Ruthenium, *ACS Sustainable Chem. Eng.*, **2022,** 10, 8342−8349.
8. Z. Ye, Z. Yang, C. Yang, M. Huang, X. Xu and Z. Ke, Disarming the alkoxide trap to access a practical $FeCl_3$ system for borrowing-hydrogen *N*-alkylation, *Org. Chem. Front.*, **2022,** 9, 4803–4817.
9. W. Li, M. Huang, J. Liu, Y.-L. Huang, X.-B. Lan, Z. Ye, C. Zhao, Y. Liu and Z. Ke, Enhanced Hydride Donation Achieved Molybdenum Catalyzed Direct *N*-Alkylation of Anilines or Nitroarenes with Alcohols: From Computational Design to Experiment, *ACS Catal.*, **2021,** 11, 10377-10382.
10. S. Michlik, T. Hille and R. Kempe, The Iridium-Catalyzed Synthesis of Symmetrically and Unsymmetrically Alkylated Diamines under Mild Reaction Conditions, *Adv. Synth. Catal.*, **2012,** 354, 847-862.
11. S. Elangovan, J. Neumann, J.-B. Sortais, K. Junge, C. Darcel and M. Beller, Efficient and selective N-alkylation of amines with alcohols catalysed by manganese pincer complexes, *Nat. Commun.*, **2016,** 7, 12641.
12. Y. Zhao, S. W. Foo and S. Saito, Iron/Amino Acid Catalyzed Direct *N*-Alkylation of Amines with Alcohols, *Angew. Chem. Int. Ed.*, **2011,** 50, 3006-3009.
13. R. Kawahara, K. Fujita and R. Yamaguchi, *N*-Alkylation of Amines with Alcohols Catalyzed by a Water-Soluble Cp*Iridium Complex: An Efficient Method for the Synthesis of Amines in Aqueous Media, *Adv. Synth. Catal.*, **2011,** 353, 1161-1168.
14. Y. Zhao and D. G. Truhlar, A new local density functional for main-group thermochemistry, transition metal bonding, thermochemical kinetics, and noncovalent interactions, *J. Chem. Phys.*, **2006,** 125, 194101.
15. T. H. Dunning Jr., P. J. Hay and H. F. Schaefer III, *Modern Theoretical Chemistry*, **1977,** 3, 1-28.
16. G. A. Petersson, A. Bennett, T. G. Tensfeldt, M. A. Al-Laham, W. A. Shirley and J. Mantzaris, A complete basis set model chemistry. I. The total energies of closed-shell atoms and hydrides of the first-row atoms, *J. Chem. Phys.*, **1988,** 89, 2193-2218.
17. G. A. Petersson and M. A. Al-Laham, A complete basis set model chemistry. II. Open-shell systems and the total energies of the first-row atoms, *J. Chem. Phys.*, **1991,** 94, 6081-6090.
18. W. Kohn and L. J. Sham, Self-Consistent Equations Including Exchange and Correlation Effects, *Phys. Rev.*, **1965,** 140, 1133-1138.
19. P. Hohenberg and W. Kohn, Inhomogeneous Electron Gas, *Phys. Rev.*, **1964,** 136, 864-871.
20. M. J. Frisch, G. W. Trucks, H. B. Schlegel, G. E. Scuseria, M. A. Robb, J. R. Cheeseman, G. Scalmani, V. Barone, G. A. Petersson, H. Nakatsuji, X. Li, A. V. Marenich, J. Bloino, B. G. Janesko, R. Gomperts, B. Mennucci, H. P. Hratchian, J. V. Ortiz, A. F. Izmaylov, J. L. Sonnenberg, D. Williams-Young, F. Ding, F. Lipparini, J. Egidi, J. Goings, B. Peng, A. Petrone, T. Henderson, D. Ranasinghe, V. G. Zakrzewski, J. Gao, N. Rega, G. Zheng, W. Liang, M. Hada, M. Ehara, K. Toyota, R. Fukuda, J. Hasegawa, M. Ishida, T. Nakajima, Y. Honda, O. Kitao, H. Nakai, T. Vreven, K. Throssell, J. A. Montgomery Jr., J. E. Peralta, F. Ogliaro, M. J. Bearpark, J.

J. Heyd, E. N. Brothers, K. N. Kudin, V. N. Staroverov, T. A. Keith, R. Kobayashi, J. Normand, K. Raghavachari, A. P. Rendell, J. C. Burant, S. S. Iyengar, J. Tomasi, M. Cossi, J. M. Millam, M. Klene, C. Adamo, R. Cammi, J. W. Ochterski, R. L. Martin, K. Morokuma, O. Farkas, J. B. Foresman and D. J. Fox, Gaussian 16, Revision C.01, Gaussian, Inc.: Wallingford CT, 2016.

21. D. Andrae, U. Häußermann, M. Dolg, H. Stoll and H. Preu, Energy-adjusted ab initio pseudopotentials for the second and third row transition elements, *Theoret. Chim. Acta*, **1990,** 77, 123-141.
22. L. E. Roy, P. J. Hay and R. L. Martin, Revised basis sets for the LANL effective core potentials, *J. Chem. Theory Comput.*, **2008,** 4, 1029-1031.
23. A. V. Marenich, C. J. Cramer and D. G. Truhlar, Universal solvation model based on solute electron density and on a continuum model of the solvent defined by the bulk dielectric constant and atomic surface tensions, *J. Phys. Chem. B*, **2009,** 113, 6378-6396.
24. C. Y. Legault, CYLview, Version 1.0b, Université de Sherbrooke, **2009,** http://www.cylview.org.
25. A. Gilbert, IQmol molecular viewer, **2017,** http://www.iqmol.org.
26. T. Lu and F. Chen, Computational Chemistry, *J. Comput. Chem.*, **2012,** 33, 580-592.
